# Integrated cryopreservation-thawing-transplantation platform for neural stem cell-based spinal cord injury repair

**DOI:** 10.1016/j.bioactmat.2026.01.024

**Published:** 2026-01-30

**Authors:** Jie Ren, Junjin Li, Hongda Wang, Haiwen Feng, Huaying Hao, Junyu Chen, Yuanquan Li, Zhengyu Xu, Chuanhao Li, Wang Jiang, Yan Wang, Xiaoyang Zhang, Xiaomeng Song, Guangzhi Ning, Jun Liang, Shiqing Feng

**Affiliations:** a Tianjin Key Laboratory of Spine and Spinal Cord, International Science and Technology Cooperation Base of Spinal Cord Injury, Department of Orthopedics, International Chinese Musculoskeletal Research Society Collaborating Center for Spinal Cord Injury, Tianjin Medical University General Hospital, Tianjin, 300070, China; b State Key Laboratory of Food Nutrition and Safety, College of Light Industry Science and Engineering, Tianjin University of Science & Technology, Tianjin, 300457, China; c Department of Sports Medicine, Peking University Third Hospital, Institute of Sports Medicine of Peking University, Beijing Key Laboratory of Sports Injuries, Engineering Research Center of Sports Trauma Treatment Technology and Devices, Ministry of Education, Beijing, 100191, China; d Orthopedic Research Center of Shandong University and Department of Orthopedics, Qilu Hospital of Shandong University, Cheeloo College of Medicine, Shandong University, Jinan, 250012, China; e Department of Orthopaedics, The Second Hospital of Shandong University, No. 247 Beiyuan Street, Tianqiao District, Jinan, 250033, China; f Department of Histology and Developmental Biology, School of Basic Medical Sciences, Tianjin Medical University, Tianjin, China; g Department of Radiology, Tianjin Medical University General Hospital, State Key Laboratory of Experimental Hematology, National Clinical Research Center for Blood diseases, Haihe Laboratory of Cell Ecosystem, Institute of Hematology & Blood Diseases Hospital, Chinese Academy of Medical Sciences & Peking Union Medical College, Tianjin, 300020, China; h Department of Laboratory Animal Sciences, Tianjin Medical University, Tianjin, 300070, China

**Keywords:** Porous microspheres, hUCMSC-Exo, Neural stem cell, 3D culture, Spinal cord injury

## Abstract

Spinal cord injury (SCI) repair lacks clinically validated restorative therapies. Transplantation of exogenous neural stem cells (NSCs) offers significant potential for therapeutic applications; however, challenges remain, including substantial cell loss, uncontrolled differentiation, and limited tissue integration within inflammatory microenvironments. Furthermore, the workflow associated with traditional NSC transplantation—including cryopreservation, thawing, transportation, and injection—remains fragmented, resulting in systemic limitations. These issues manifest as reduced cell viability and stemness, an elevated risk of contamination, and dosing inaccuracies. All these significantly impede clinical translation. An integrated system for NSC preservation, transport, and transplantation is required to meet the following criteria: (i) maintenance of high cell viability and stemness post-cryopreservation and thawing; (ii) modulation of the acute-phase immune microenvironment; (iii) regulation of the differentiation fate of transplanted NSCs; (iv) injectable, standardized, and closed-system operation. To meet these requirements, we established a comprehensive cryopreservation, thawing, and transplant (CTT) integrated platform. Utilizing the bioactive material PM-BMH@Exo, this platform enables seamless end-to-end workflow integration through a mechanism that preserves bioactivity. It not only ensures high viability retention and directed differentiation of NSCs but also effectively mitigates the rapid viability decline of cells observed after traditional cryopreservation. Furthermore, the system enables closed-loop operations spanning cryopreservation, thawing, and minimally invasive injection. It breaks through systemic bottlenecks from multi-step procedures, comprehensively enhancing the timeliness and standardization of therapeutic interventions. We systematically evaluated the system's feasibility and efficacy via in vitro and in vivo experiments. This study presents a technologically viable and clinically compatible pathway with potential applications for SCI repair.

## Highlights

• BMH@Exo alters the inflammatory microenvironment associated with SCI by facilitating macrophage polarization towards a tissue-repair phenotype, thereby creating more favorable conditions for NSCs engraftment.
• HUCMSC-Exo, an essential element of the CTT platform, promotes the differentiation of NSCs into neurons through activation of the PI3K-Akt and calcium signaling pathways.
• The development of a three-dimensional culture system utilizing Poly (D, L-lactic acid) porous microspheres offers an optimal microenvironment to support the growth, proliferation, and differentiation of NSCs.
• Minimally invasive injection of the CTT platform rebuilds neural circuits through neurogenesis and remyelination, thereby restoring motor function in SCI rats.
• The end-to-end CTT workflow integrates cryostorage, thawing, transport, and injection, addressing the fragmentation of conventional NSC-based SCI therapies.

## Graphical abstract

**Figure:**
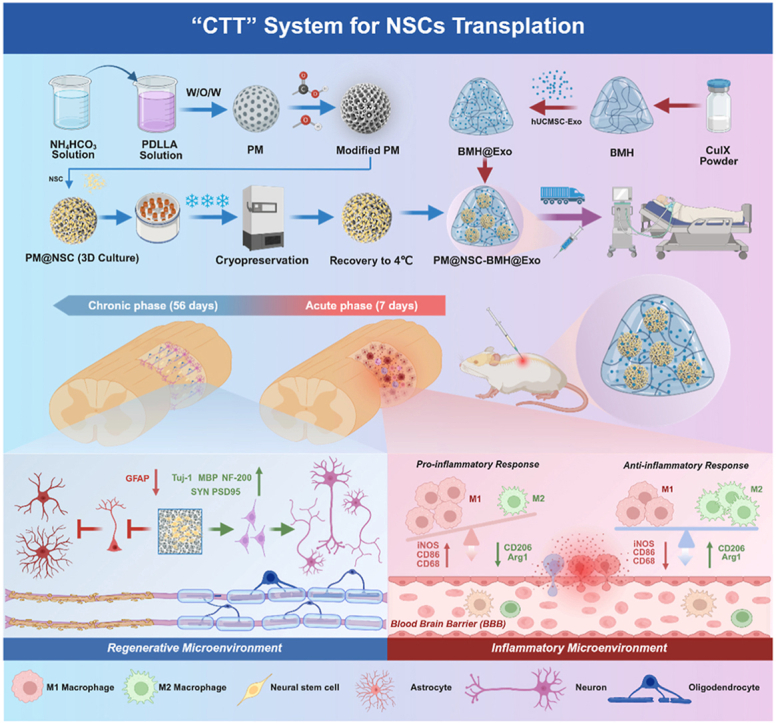

## 1 Introduction

Spinal Cord Injury (SCI) presents substantial physical and social challenges, and the development of effective clinical treatments remains hindered by the intricate cascade of secondary injuries that ensue following the primary trauma [1,2]. Although transplantation of exogenous neural stem cells (NSCs) offers potential for lesion repopulation and neural network reconstruction [3], its clinical translation is impeded by two principal obstacles: an adverse post-injury microenvironment and fragmented workflows in clinical cell processing. Initially, the pathological microenvironment present at the injury site serves as a significant barrier to successful engraftment. Inflammatory cascades, oxidative stress, and the formation of glial scars collectively establish a “hostile soil,” substantially restricting the survival, proliferation, and neuronal differentiation of transplanted NSCs [[4], [5], [6]]. Without appropriate modulation of this inflammatory milieu, transplanted cells frequently undergo apoptosis or differentiate into astrocytic lineages, thereby limiting their potential for meaningful functional repair [7,8]. Second, an equally important concern is the systemic challenge presented by the conventional “cryopreservation - thawing - transplantation” workflow. Standard protocols require a segmented, multi-stage process in which stem cells are cryopreserved, thawed, washed, and subsequently transplanted. Increasing evidence suggests that this fragmented approach contributes to a scenario of high attrition and low integration [9,10]. Mechanical and osmotic stresses encountered during freeze-thaw cycles, together with extended exposure resulting from manual transfers, contribute to a significant reduction in both cell viability and paracrine function prior to patient administration [[11], [12], [13]]. Furthermore, these repetitive manual interventions increase the risks of contamination and dosage inaccuracy, severely limiting clinical standardization [14]. Consequently, it is essential to implement a comprehensive strategy that both addresses the challenges of the adverse microenvironment and optimizes the delivery process in order to maintain cell potency.

To overcome these dual hurdles, we turned to functional biomaterials and bioactive components. Human umbilical cord mesenchymal stem cell-derived exosomes (hUCMSC-Exos) have been identified as effective modulators of the immune microenvironment, demonstrating the ability to suppress inflammation and facilitate neuroregeneration with greater stability and safety compared to whole-cell therapies [[15], [16], [17], [18], [19]]. Poly (D, L-lactic acid) (PDLLA) porous microspheres (PM) function as efficient three-dimensional carriers that facilitate stem cell adhesion and proliferation [[20], [21], [22]]. Biomimetic Matrix Hydrogels (BMH) provide an in situ supportive scaffold and serve as a minimally invasive delivery vehicle [23]. In this study, we developed a novel integrated Cryopreservation-Thawing-Transplantation (CTT) platform to overcome existing bottlenecks. By encapsulating neural stem cells (NSCs) within a composite (PM-BMH@Exo), our objective is to establish a comprehensive “closed-loop” solution. This design utilizes the porous microspheres for physical support and the exosome-enriched hydrogel for immunomodulation, protecting NSCs from the hostile host environment. Crucially, the platform is designed to sustain high cell viability throughout the storage and retrieval process, allowing for direct, minimally invasive injections without the need for detrimental washing or transfer steps. It is postulated that the combination of biomaterial functionalization with process integration will markedly improve neural stem cell survival and differentiation, providing a strong translational approach for spinal cord injury repair.

**Fig. 1:**
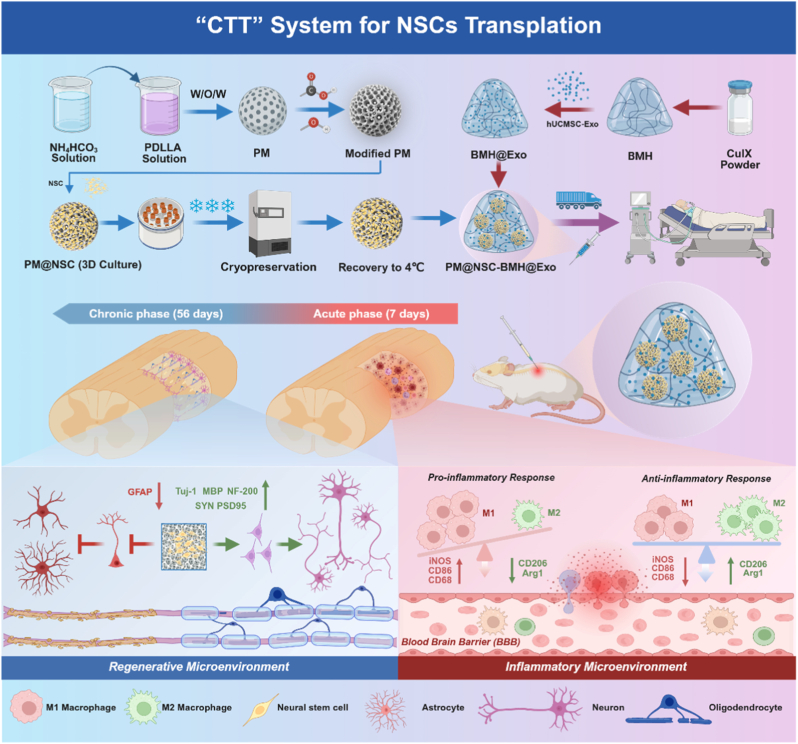
Schematic illustration of the Cryopreservation–Thawing–Transplantation (CTT) system for NSC transplantation. This schematic illustrates the construction and mechanism of the CTT system designed for SCI repair. The core components comprise: (i) PDLLA porous microspheres (PMs), (ii) biomimetic matrix hydrogel (BMH), (iii) NSCs, and (iv) hUCMSC-Exos. PDLLA solution was emulsified via a W/O/W method to synthesize PMs. Surface modification through hydrophilic crosslinking enabled NSC loading, forming PM@NSC complexes that facilitate high viability of NSCs post-cryopreservation. BMH encapsulating hUCMSC-Exos (denoted BMH@Exo) was prepared via self-assembly under physiological conditions (CulX). The final synergistic carrier, PM@NSC-BMH@Exo, integrates both complexes, allowing for direct, minimally invasive injection into SCI sites. Following transplantation, the system actively remodels the pathological microenvironment by attenuating inflammatory responses during the acute phase to promote the survival and differentiation of exogenous NSCs. In the chronic phase, it facilitates axonal regeneration and myelination, supporting neural network reconstruction and comprehensive functional recovery. Based on these features, this system enables spatiotemporally adaptable transplantation of NSCs with high efficiency and quality controllability. It not only mitigates the risk of cell viability loss during traditional cryopreservation and transport, but also eliminates cellular loss and contamination hazards through its integrated closed-loop workflow. Post-transplantation, the dual-phase synergistic regulation strategy further enhances NSC survival and directional differentiation, thereby establishing a standardized end-to-end therapeutic protocol from preservation and transport to implantation.

This integrated platform demonstrates the construction and mechanism of a cryopreservation-thawing-transplantation (CTT) system designed for SCI repair. The core components comprise: (i) PDLLA porous microspheres (PMs), (ii) biomimetic matrix hydrogel (BMH), (iii) NSCs, and (iv) hUCMSC-Exos. PDLLA solution was emulsified via a W/O/W method to synthesize PMs. Surface modification through hydrophilic crosslinking enabled NSC loading, forming PM@NSC (3D Culture) complexes that facilitate high viability of NSCs post-cryopreservation. BMH encapsulating hUCMSC-Exos (denoted BMH@Exo) was prepared via self-assembly under physiological conditions (CulX). The final synergistic carrier, PM@NSC-BMH@Exo, integrates both complexes, allowing for direct, minimally invasive injection into SCI sites. Following transplantation, the system actively remodels the pathological microenvironment by attenuating inflammatory responses during the acute phase to promote the survival and differentiation of exogenous neural stem cells. In the chronic phase, it facilitates axonal regeneration and myelination, supporting neural network reconstruction and comprehensive functional recovery. Based on the features, this system enables spatiotemporally adaptable transplantation of NSCs with high efficiency and quality controllability. It not only mitigates the risk of cell viability attenuation during traditional cryopreservation and transportation but also eliminates cellular loss and contamination hazards through its integrated closed-loop workflow. Post-transplantation, the dual-phase synergistic regulation strategy further enhances NSC survival and directional differentiation, thereby establishing a standardized end-to-end therapeutic protocol from preservation, transit to implantation (Fig. 1).

## 2 Results

### 2.1 Preparation, modification, and characterization of biomimetic matrix hydrogel (BMH) + porous microsphere (PM) composites

**Fig. 2:**
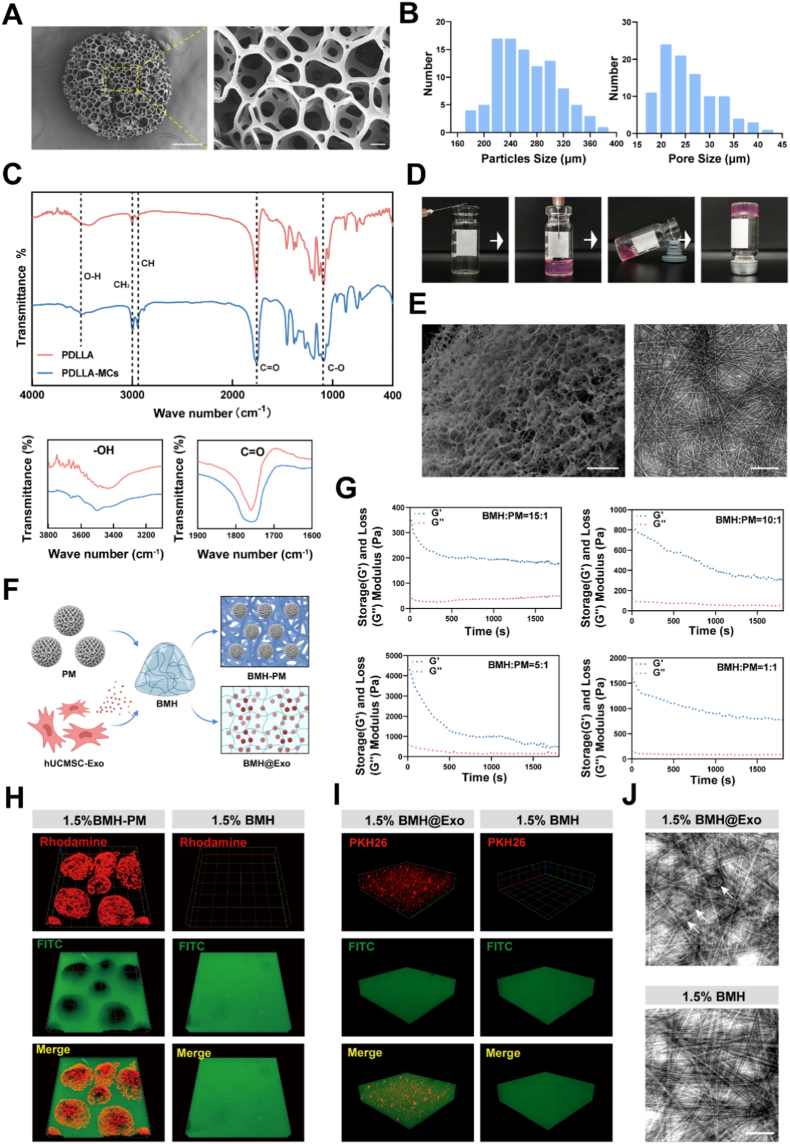
Preparation, modification, and characterization of PDLLA porous microspheres (PM), biomimetic matrix hydrogel (BMH), and PM-BMH Components. A) SEM images of PDLLA porous microspheres at different magnifications. Scale bar (left): 100 μm. Scale bar (right): 10 μm B) Particle size and pore size distribution of the microspheres. C) FTIR spectra of PDLLA and hydrolyzed PDLLA microspheres, with magnified views of -OH and C=O regions. D) Illustration of the injectable properties and self-healing capabilities of hydrogels. E) SEM and TEM images of the biomimetic matrix hydrogel. Scale bar (SEM): 50 μm. Scale bar (TEM): 200 nm F) Schematic representation of the preparation of BMH-PM and BMH@Exo composites. G) Rheological curves of BMH-PM composites at varying mass ratios. H) Confocal imaging of rhodamine-labeled PM embedded in FITC-labeled 1.5 wt% BMH. I) Confocal images of PKH26-labeled Exo in FITC-labeled BMH composites. J) TEM image of hUCMSC-Exo-loaded nanofiber networks in hydrogels. Scale bar: 200 nm.

To construct a biomimetic scaffold capable of supporting cell anchorage and nutrient transport, we first fabricated poly (DL-lactic acid) (PDLLA) porous microspheres via a double emulsion technique (Fig. S1A). These microspheres exhibit a uniform spherical structure (Fig. 2A) with uniformly distributed diameters of 270.73 ± 47.69 μm (mean ± SD, n = 50, Fig. 2B). A key feature of this structure is its interconnected pores, having an average pore size of 26.11 ± 5.82 μm (Fig. 2B), which facilitates cell adhesion and nutrient diffusion. PDLLA microspheres, both before and after hydrolysis, were prepared for contact angle measurements. The surface contact angle of the PDLLA porous microspheres was approximately 117.35°, indicating hydrophobic properties. However, after hydrolysis, the contact angle of the PDLLA porous microspheres decreased to 68.5°, indicating enhanced hydrophilicity due to NaOH hydrolysis modification (Fig. S2A). Under the hydrolysis of sodium hydroxide, the ester bonds in the macromolecules of PDLLA porous microspheres are cleaved, introducing hydroxyl and carboxyl groups on the microsphere surface (Fig. S1B). The enhanced peaks of hydroxyl and carboxyl groups in the Fourier-transform infrared spectroscopy (FTIR) spectrum were consistent with the contact angle results, corroborating the increased hydrophilicity after hydrolysis (Fig. 2C). To assess the in vitro degradation behavior of PDLLA porous microspheres, samples were incubated in phosphate-buffered saline (PBS, pH 7.4) for four weeks, and morphological alterations were examined by scanning electron microscopy (SEM). The findings indicated a progressive increase in surface micropores, deformation of the regular porous architecture, thinning of pore walls, disruption of spherical structure, and clear evidence of degradation over time (Fig. S2B). This controlled degradation profile is essential for tissue engineering, as it provides temporary mechanical support while gradually creating space for tissue regeneration.

Next, to mimic the extracellular matrix (ECM) of the spinal cord and provide a conducive microenvironment, the microstructure of the hydrogel was studied using transmission electron microscopy (TEM) and SEM. The microstructure of the hydrogel was studied using TEM and SEM. TEM observations revealed a porous, highly ordered, distributed network structure within the bio-mimetic matrix hydrogel solution. SEM images further confirmed the formation of a highly porous structure, consistent with TEM findings. The dense nanofiber network effectively retains moisture and forms a three-dimensional structure for support (Fig. 2E). To examine the characteristics of biomimetic matrix hydrogels across varying concentrations, precursor solutions were prepared at 0.5 wt%, 1.0 wt%, 1.5 wt%, and 2.0 wt%. Remarkably, these solutions spontaneously formed hydrogels within 5 min at room temperature. The 0.5 wt% sample yielded a liquid-like hydrogel, attributable to its relatively low concentration (Fig. S3A). Furthermore, the critical aggregation concentration was determined to be 84 μg/mL, suggesting that ordered self-assembly is initiated when this threshold is surpassed (Fig. S4). The secondary structure of the biomimetic matrix hydrogel was analyzed using FTIR. The second derivative spectrum clearly indicates the presence of α-helix, β-sheet, random coil, and β-turn structures in the hydrogel (Fig. S5). To confirm these FTIR findings, we used a Thioflavin T (THT) fluorescent probe, which specifically targets β-sheet structures. The experiments showed that the amount of β-sheet in the hydrogel increases as the sample concentration rises, indicating a concentration-dependent increase in β-sheet content (Fig. S6). All experiments were conducted at concentrations above the critical aggregation concentration, ensuring that the biomimetic matrix hydrogels had formed a stable and complete secondary structure. This stable secondary structure provides a solid mechanical foundation for the hydrogel's application in vivo.Rheological properties were subsequently evaluated to determine the optimal formulation for clinical injectability. Rheological test results indicated that the storage modulus (G′) of hydrogel scaffolds was positively correlated with their concentration. Furthermore, a broad linear viscoelastic region was consistently observed across all tested concentrations. The 1.0 wt% hydrogel exhibited a mechanical strength of approximately 207 Pa. The 1.5 wt% hydrogel was stable and had a mechanical strength of 404 Pa, forming a typical solid gel capable of smooth injection through a 25G syringe for deep tissue delivery (Fig. S3B). The self-assembly of the biomimetic matrix hydrogel occurs via intermolecular forces, enabling partial self-healing. For instance, when extracted and reinjected through a syringe, the hydrogel gradually recombines into a complete structure within a brief time (Fig. 2D). The outstanding ability of this hydrogel to recombine after damage suggests that its internal architecture consists of a dynamic, self-assembled network. Furthermore, in three consecutive cycles of shear thinning and recovery tests, the strength (G′) of the biomimetic matrix hydrogel was able to recover to its initial value, demonstrating outstanding fatigue resistance. Additionally, comparing experimental data across four different concentrations reveals that the properties of shear thinning and self-healing remain consistent regardless of concentration changes (Fig. S7). Controlling hydrogel swelling helps maintain gel strength and avoids undesirable compressive damage to surrounding tissues. The swelling behavior exhibited a concentration-dependent trend, with the swelling ratio of hydrogel decreased gradually as the hydrogel concentration increased. The 0.5 wt% hydrogel reached a swelling ratio of 46.42 ± 0.81 % by the sixth week, with hydrogels at concentrations of 1 wt% or higher exhibiting the least swelling during the observation period (Fig. S3C). Biodegradability is also crucial for the function of hydrogels as temporary scaffolds. Under the action of naturally occurring collagenase in vivo, the biomimetic matrix hydrogels degraded rapidly, with approximately 90 % degradation occurring within 12 days. However, the erosion process of the biomimetic matrix hydrogel was slow. After 15 days in PBS, the erosion rate approached 30 %, indicating its good stability in the absence of enzymes (Fig. S8). The characteristic of rapid enzyme-promoted degradation combined with slow physical erosion is a key factor that must be balanced when designing hydrogel scaffolds for clinical applications. Based on these comprehensive characterizations, the 1.5 wt% hydrogel was selected for composite construction due to its optimal balance of rheological properties, injectability, and stability to better mimic natural spinal cord tissue.

Having characterized the individual components, we integrated them to form the microsphere-hydrogel composite and evaluated its mechanical synergy (Fig. 2F). Results showed that the composite exhibited higher mechanical strength than the hydrogel alone, with mechanical strength increasing as the proportion of microspheres rose (Fig. 2G). To determine the optimal composite concentration, the effects of different mass ratios of hydrogel to porous microspheres on the stability and rheological properties of the composite were investigated (Figs. S9 and 2G). Based on these findings, a 10:1 mass ratio was chosen for subsequent experiments. In order to accurately examine the composite structure, a confocal scanning imaging system was employed to visualize the three-dimensional arrangement of microspheres within the hydrogel scaffold network. The analysis indicated that rhodamine-labeled PDLLA microspheres and FITC-labeled hydrogel scaffolds were integrated into a uniform, interpenetrating composite (Fig. 2H). This uniform integration ensures that the composite can provide consistent mechanical support and evenly distributed biological signals throughout the injury site.

Finally, to ensure the biological potency of the therapeutic cargo within the system, we validated the identity of the hUCMSCs and their derived exosomes. To assess the multipotent differentiation potential of hUCMSCs, Alcian Blue, Oil Red O, and Alizarin Red staining were employed to identify chondrogenic, adipogenic, and osteogenic differentiation, respectively (Fig. S11). Surface markers of hUCMSCs were identified via flow cytometry. Results showed that the stem cells used in this experiment highly expressed mesenchymal stem cell markers CD73, CD90, and CD105, while hematopoietic and immune phenotype markers CD11b, CD19, CD34, CD45, and HLA-DR were not detected (Fig. S12). Transmission electron microscopy (TEM) analysis demonstrated that exosomes derived from hUCMSCs displayed the characteristic cup-shaped morphology (Fig. S10A). Furthermore, nanoparticle tracking analysis (NTA) indicated that these exosomes exhibited an average diameter of 126.2 ± 43.3 nm, with a peak diameter measured at 101.6 nm (Fig. S10B). To further analyze the specific surface markers of exosomes, Western blot analysis was performed. Results indicated that hUCMSC-derived exosomes highly expressed the exosome-specific markers CD63, CD9, and TSG101, while GAPDH was not expressed (Fig. S10C). These results confirm the successful isolation of hUCMSC and hUCMSC-Exo. The three-dimensional distribution of exosomes within the hydrogel scaffold network was observed using a confocal scanning imaging system. PKH-26-labeled exosomes were uniformly distributed within the FITC-labeled hydrogel scaffold (Fig. 2I). Furthermore, the TEM results provided additional validation for the above findings (Fig. 2J). Collectively, these data confirm the successful fabrication of a multicomponent, injectable composite (BMH@Exo-PM) with optimized physicochemical properties and biological cargo, laying the groundwork for subsequent functional evaluations.

### 2.2 Biocompatibility and biodegradability of composite (BMH@Exo-PM)

Having successfully engineered the composite system, the next critical step was to verify its compatibility with NSCs before in vivo application. To evaluate the in vitro cytotoxicity of the composite, live/dead cell staining and CCK-8 assays were conducted. NSCs were treated with the composite and its individual components in transwell chambers for 1, 3, and 7 days, and the proliferation activity of NSCs in the lower chamber was assessed using the CCK-8 assay. Compared to the blank control group, cell viability in the BMH and PM groups showed no significant difference. In contrast, the Exo and BMH + PM + Exo groups exhibited significantly increased cell viability, with the most pronounced increase observed at 3 days of treatment (Fig. S13C–E). We selected the third day of treatment to evaluate the live/dead staining results of NSCs. The survival rates from the Control group to the BMH + PM + Exo group were 95.55 ± 6.37 %, 94.72 ± 8.86 %, 95.72 ± 6.2 %, 95.99 ± 4.35 %, and 97.47 ± 2.38 %, respectively (Fig. S13A and B). The experimental findings indicated that the materials exhibited excellent biocompatibility with NSCs across all groups. Notably, the Exo-containing groups displayed pronounced cell proliferation and robust spheroid formation (Fig. S13A). These results confirm that the composite system not only exhibits negligible cytotoxicity but also actively creates a supportive niche for NSC survival and expansion.

Beyond cellular compatibility, the degradation profile of the scaffold in a physiological environment is pivotal for preventing chronic foreign body reactions. Cy7-labeled porous microspheres were subcutaneously implanted into the dorsal region of rats to evaluate their in vivo degradation, with in vivo imaging conducted on days 3, 7, 14, and 21 post-implantation. Results showed that the porous microspheres were completely degraded by 3 weeks post-implantation, indicating excellent in vivo degradation performance (Fig. S25). This appropriate degradation rate ensures that the material provides temporary mechanical support during the acute phase of injury without causing long-term material accumulation. Finally, to ensure the clinical translational potential of our system, we assessed systemic biosafety to rule out any organ-level toxicity. Histopathological examination of major organs—including the heart, lungs, liver, spleen, and kidneys—in rats receiving the platform transplantation revealed no evidence of degeneration, atrophy, or necrosis (Fig. S26). To assess the long-term biosafety and potential immunological responses associated with the composite, this study conducted blood biochemical assays and complete blood count (CBC) analyses in rats two months following transplantation. The biochemical evaluations encompassed essential hepatic function markers—alanine transaminase (ALT), aspartate transaminase (AST), and alkaline phosphatase (ALP)—as well as principal renal function indicators, including blood urea nitrogen (BUN), creatinine (CREA), and uric acid (UA). CBC analyses principally examined immune-related cellular subsets, specifically white blood cells (WBC), lymphocytes (LYM), and neutrophils (NEU). Results showed that there were no statistically significant differences in the levels of liver and renal function indicators or the counts of immune-related cells between the experimental group and the control group (P > 0.05) (Figs. S27 and S28). Collectively, these data demonstrate that the composite exhibits excellent long-term biosafety and does not induce systemic immune rejection, confirming its safety profile for application in SCI treatment.

### 2.3 The mechanisms of the composite (BMH@Exo-PM) induced NSCs to differentiate into neurons in vitro

**Fig. 3:**
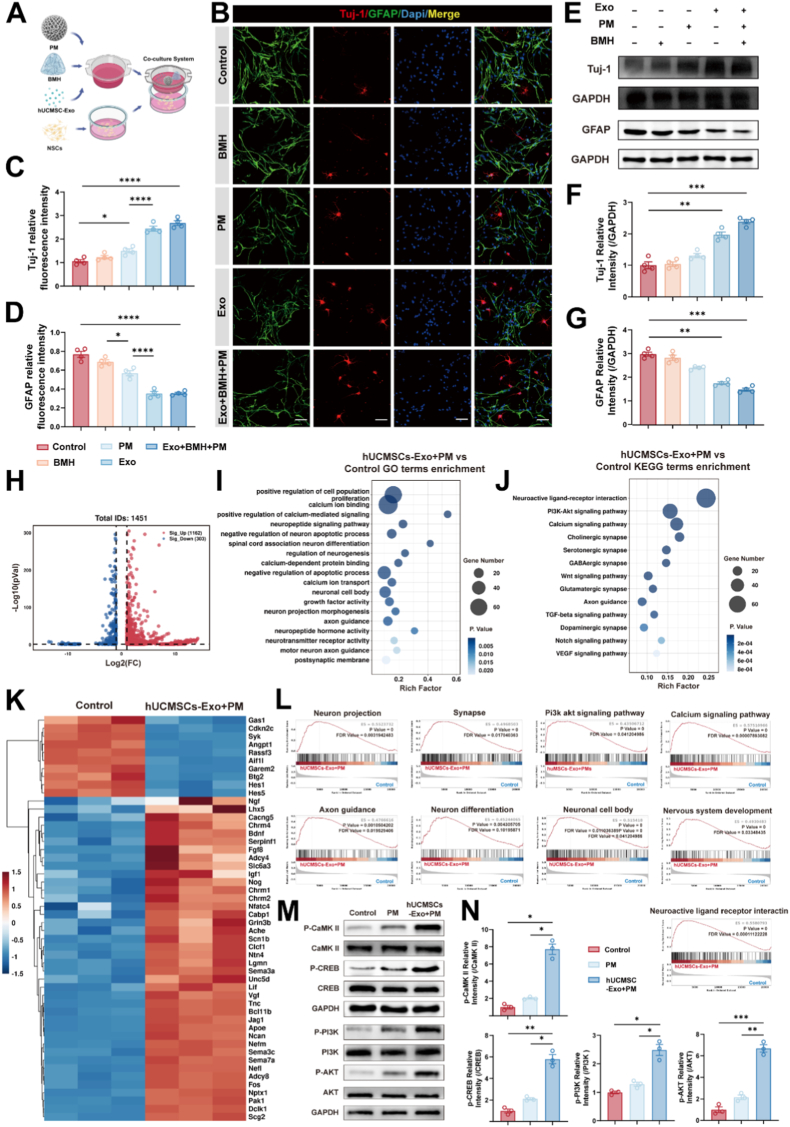
Mechanisms of NSC Differentiation into Neurons Promoted by Composite In Vitro. A) Schematic of the interactive system with NSCs and the composites. B) Representative confocal images of NSCs treated with distinct groups for 72 h. NSCs were stained with Tuj-1 (red), GFAP (green), and DAPI (blue). Scale bar: 50 μm. C-D) Quantitative analysis of Tuj-1 (C) and GFAP (D) fluorescence intensity in each group (n = 4). E) Western blot bands of Tuj-1 and GFAP protein expression in NSCs treated with separate groups. F-G) Quantitative analysis of Tuj-1/GAPDH (F) and GFAP/GAPDH (G) ratios in each group (n = 3). H) Volcano plots of DEGs in the hUCMSC-Exo + PM vs. control. DEGs are defined as |log2FC| ≥ 1 with q < 0.05. I-J) GO and KEGG pathway enrichment analysis of DEGs in NSCs after intervention with hUCMSC-Exo + PM. K) Heatmap showing the expression levels of significantly altered genes in the hUCMSC-Exo + PM and Control. L) GSEA showing pathways significantly positively correlated with DEGs in the hUCMSC-Exo + PM group. Enrichment scores (ES), P values, and false discovery rates (FDR) values are indicated for each pathway. M) Western blot bands of p-CaMK II, CaMK II, p-CREB, CREB, p-PI3K, PI3K, p-AKT, and AKT protein expression in NSCs treated with distinct groups. N) Quantitative analysis of p-CaMK II/CaMK II, p-CREB/CREB, p-PI3K/PI3K, and p-AKT/AKT ratios (n = 3). All data are presented as the mean ± SEM. ∗p < 0.05, ∗∗p < 0.01, ∗∗∗p < 0.001, ∗∗∗∗p < 0.0001.

To determine if the composite could direct NSC fate toward a specific lineage, we established an interactive system. The composite and its individual components were placed in the upper chamber, while NSCs were cultured in the lower chamber (Fig. 3A). This study focused on primary NSCs to observe the effects of the composite and its individual components on NSC differentiation in vitro. First, primary NSCs were extracted and identified (Fig. S14). Passage 2 (P2) NSC spheres were dissociated into single cells, seeded, and treated with the composite in differentiation-inducing medium for 3 days. Immunofluorescence results showed that, compared to the control group, the fluorescence intensity of Tuj1 significantly increased, while the fluorescence intensity of GFAP significantly decreased in the Exo group and the PM + BMH + Exo group. The PM group also exhibited a particular effect in promoting differentiation, due to the degradation of the porous microspheres (Fig. 3B, C, D). Western blot results indicated that, compared to the control group, the Exo group and the PM + BMH + Exo group exhibited significantly increased Tuj1 protein expression and decreased GFAP protein expression (Fig. 3E, F, G). These results suggest that the exosome-containing composite functions as a potent neurogenic inducer, effectively promoting neuronal commitment while suppressing astrocytic differentiation.

Having confirmed the phenotypic shift, we next sought to decode the underlying molecular landscape using RNA sequencing. RNA sequencing analysis was performed on NSC spheres treated with PM + hUCMSC-Exo or PM for 3 days. A total of 1465 differentially expressed genes (DEGs) were identified between the hUCMSC-Exo + PM group and the control group, including 1162 upregulated genes and 303 downregulated genes (|log2FC|≥1 & q < 0.05) (Fig. 3H). In parallel, 603 DEGs were found between the PM group and the control group (|log2FC|≥1 & q < 0.05) (Fig. S15A).

Analysis of the hUCMSC-Exo + PM group revealed a robust upregulation of genes critical for neural development and differentiation (Ngf, Lhx5, Fgf8, Jag1, Ntn4, Bdnf, Nefm, Nefl, Clcf1, Bcl11b), neural signal transduction (Adcy4, Adcy8, Chrm1, Chrm2, Chrm4, Scn1b, Cabp1, Ache, Nfatc4), neurotrophic support (Apoe, Vgf, Serpinf1, Tnc, Igf1, Lif), neural connectivity and guidance (Sema3a, Sema3c, Sema7a, Unc5d, Pak1), and synaptic function and plasticity (Grin3b, Nptx1, Ncan, Fos). Conversely, genes associated with maintaining the undifferentiated state of NSCs (Hes1, Hes5) were downregulated (Fig. 3K). By comparison, in the PM group, upregulated DEGs were primarily related to neural development and differentiation (Fezf1, Isl1, Bmp5), neural signal transduction and synaptic plasticity (Grin3b), neurotrophic support (Clcf1), and neural connectivity and guidance (Sema3c) (Fig. S15B). Gene Ontology (GO) enrichment analysis showed that, after hUCMSC-Exo + PM intervention, genes were significantly enriched in biological processes or functional categories such as positive regulation of cell population, spinal cord association neuron differentiation, positive regulation of calcium-mediated signaling, calcium ion binding, neuronal cell body, neuron projection morphogenesis, and axon guidance (Fig. 3I). After PM intervention, DEGs were enriched in biological processes such as positive regulation of cell population, negative regulation of neuron apoptotic process, and brain-derived neurotrophic factor binding (Fig. S15C). Kyoto Encyclopedia of Genes and Genomes (KEGG) pathway analysis further distinguished the groups: while PM intervention mainly influenced metabolic adaptation, the hUCMSC-Exo + PM group showed enrichment in the Calcium signaling pathway, PI3K-Akt signaling pathway, and various synaptic pathways (Cholinergic, Serotonergic, GABAergic) (Fig. 3J–S15D). These transcriptomic profiles reveal that hUCMSC-Exo acts as a master regulator, reprogramming the genetic network of NSCs by activating critical neurogenic and signaling pathways. Crucially, Gene set enrichment analysis (GSEA) confirmed that pathways such as Neuron projection, Synapse, and Axon guidance were significantly positively correlated only after hUCMSC-Exo intervention (Fig. 3L), whereas the PM alone group showed no such trend (Fig. S16). These transcriptomic profiles reveal a clear division of labor: while PM degradation provides metabolic and structural cues, hUCMSC-Exo acts as the master regulator, reprogramming the genetic network of NSCs to activate critical neurogenic and signaling pathways. Guided by the KEGG pathway and GSEA analysis which highlighted the Calcium and PI3K-Akt signaling pathways, we proceeded to validate these targets at the protein level. This study assessed the phosphorylation levels and total protein levels of key proteins (CaMKⅡ, CREB, PI3K, and AKT) involved in these pathways across the control group, PM-alone treatment group, and PM + hUCMSC-Exo combined treatment group. GAPDH served as the loading control for normalizing band intensity. Western blot results demonstrated that, compared with the control group and PM-alone treatment group, the expression levels of phosphorylated CaMKⅡ (p-CaMKⅡ), phosphorylated CREB (p-CREB), phosphorylated PI3K (p-PI3K), and phosphorylated AKT (p-AKT) were significantly upregulated in the PM + hUCMSC-Exo treatment group. In contrast, the total protein levels of these proteins (CaMKⅡ, CREB, PI3K, and AKT) exhibited no significant differences across all groups (Fig. 3M and N). These findings provide evidence that the combined treatment of PM + hUCMSC-Exo effectively activates the PI3K-Akt and calcium signaling cascades via phosphorylation, validating the mechanistic predictions from our transcriptomic screen.

Beyond cellular differentiation and signaling activation, the ultimate functional endpoint of neural repair is the formation of integrated synaptic networks. We found that pathways related to glutamatergic synapse, cholinergic synapse, GABAergic synapse, dopaminergic synapse, and serotonergic synapse were significantly upregulated in the hUCMSC-Exo + PM group (Fig. S17). To further clarify the regulatory effect of the composite on the expression of synapse-related proteins in NSCs at the protein level, this study detected the expression levels of the postsynaptic marker protein PSD95 and presynaptic marker protein SYN in NSCs from different intervention groups using immunofluorescence staining. The results showed that the mean fluorescence intensities of PSD95 and SYN in NSCs were significantly higher in the hUCMSC-Exo group and the Exo + BMH + PM composite group than in the control group; the PM alone group only exhibited a slight increase in mean fluorescence signal, while the fluorescence signal in the BMH alone group showed no significant difference from that in the control group (Fig. S18). These results indicate that the composite does not merely induce neuronal phenotype expression but actively promotes the synthesis of key synaptic machinery (SYN, PSD95). The enrichment of these pathways, combined with immunofluorescence data, further confirms the core role of hUCMSC-Exo in regulating synaptic function and functionally remodeling the neural network. The establishment of these synaptic connections provides the necessary molecular foundation for restoring neural circuitry, offering a promising explanation for the functional recovery observed in spinal cord injury models.

### 2.4 Characterization and molecular mechanisms of the NSC 3D culture system based on PDLLA porous microspheres

**Fig. 4:**
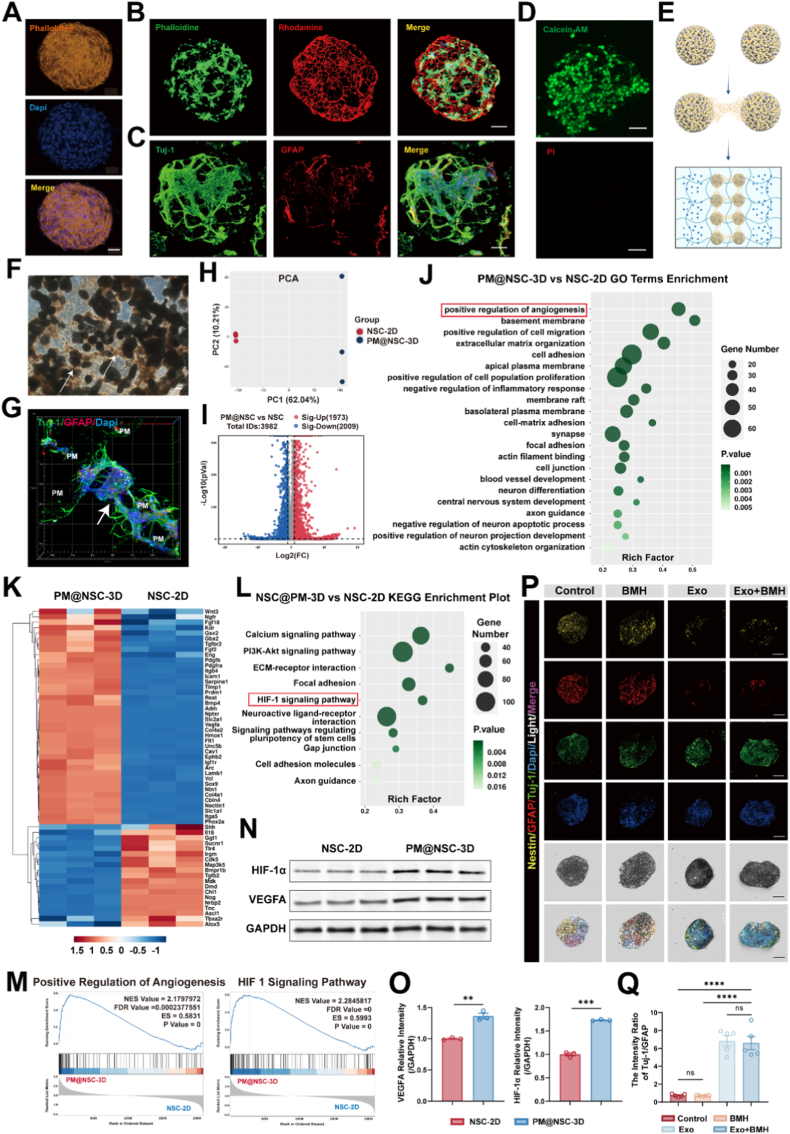
3D neural network formation and underlying molecular mechanisms in PM@NSC microsphere culture system A) Representative confocal images showing phalloidin-stained F-actin (orange) and DAPI-stained nuclei (blue) in hUCMSCs cultured on porous microspheres, demonstrating cytoskeletal alignment and cell distribution. Scale bar: 50 μm B) Representative confocal images of rhodamine-labeled microspheres (red) with phalloidin-stained NSCs (green), highlighting uniform cell distribution and attachment in the 3D microenvironment. Scale bar: 50 μm C) Representative confocal images of Tuj-1 (green) and GFAP (red) in NSCs cultured in neuronal differentiation medium for 3 days. Scale bar: 50 μm. D) Calcein-AM (green: live cells) and PI (red: dead cells) staining of NSCs cultured on porous microspheres, indicating high cell viability. Scale bar: 50 μm E) Schematic illustration demonstrating neural network formation in 3D culture. Tuj1-positive neuronal projections extend from NSCs-laden microspheres to bridge adjacent microspheres, forming neural interconnect structures. F) Brightfield micrograph of PM@NSC culture: Black spherical microcarriers interconnected by brownish-yellow NSC clusters (white arrows) forming bridging networks. Scale bar: 200 μm G) Representative confocal image demonstrating Tuj-1^+^ neurites (green) assembling into a neural bridge structure connecting isolated PM-labeled microspheres. The white arrow designates the bridge, illustrating how interconnections between microspheres lead to the formation of a neural network. H) Principal component analysis (PCA) showing the clustering of gene expression profiles in the PM@NSC-3D and NSC-2D groups. I) Volcano plots of DEGs in the PM@NSC-3D vs. NSC-2D. DEGs are defined as |log2FC| ≥ 1 with q < 0.05. J) GO enrichment analysis of DEGs in NSCs after intervention with PM@NSC-3D and NSC-2D. K) Heatmap showing the expression levels of significantly altered genes in the PM@NSC-3D and NSC-2D group. L) KEGG pathway enrichment analysis of DEGs in NSCs after intervention with PM@NSC-3D and NSC-2D. M) GSEA showing pathways significantly positively correlated with differentially expressed genes in the PM@NSC-3D and NSC-2D. Normalized Enrichment Score (NES), Enrichment scores (ES), P values and FDR values are indicated for each pathway. N) Western blot bands of HIF-1α and VEGFA expression in each group. O) Quantitative analysis of HIF-1α/GAPDH and VEGFA/GAPDH ratios in each group (n = 3). P) Representative confocal images of NSCs cultured on porous microspheres treated with separate groups for 72 h. NSCs were stained with Nestin (yellow), Tuj-1 (green), GFAP (red), and DAPI (blue). Scale bar: 100 μm. Q) Quantitative analysis of Tuj-1/GFAP intensity ratio (n = 5). All data are presented as the mean ± SEM. ∗∗p < 0.01, ∗∗∗p < 0.001, ∗∗∗∗p < 0.0001.

To overcome the spatial limitations of traditional monolayer cultures and better simulate the in vivo niche, we evaluated the potential of the “microsphere-hydrogel” component as a 3D carrier for NSC transplantation. Under 3D culture, phalloidin staining revealed a uniform F-actin filament network formed by hUCMSCs inside the microspheres, demonstrating favorable cell morphology (Fig. 4A). Furthermore, using phalloidin to label NSCs and rhodamine to label the porous microspheres, immunofluorescence imaging clearly showed that the cytoskeleton was orderly arranged along the surface and internal pores of the microspheres, indicating adequate adhesion and expansion of NSCs in the 3D microenvironment (Fig. 4B). After culturing PM@NSCs in neuronal induction differentiation medium for 3 days, immunofluorescence staining was performed. The results showed that neurospheres extended protrusions along the surface and internal pores of the porous microspheres, forming complex neural network structures. Critically, unlike the flat morphology seen in 2D cultures, the 3D environment facilitated extensive extension and networking of cellular protrusions, indicating a superior capacity for neuronal morphogenesis (Fig. 4C). Live/dead cell staining further confirmed the excellent biocompatibility of the culture system (Fig. 4D). Notably, under 3D conditions, initially dispersed PM@NSCs were able to establish cellular connections with adjacent NSC-loaded microspheres, ultimately forming an interconnected neural network spanning multiple microspheres (Fig. 4E). Light microscopy observations revealed that the porous microspheres loaded with NSCs, which were initially discrete and independent, gradually formed fused sheet-like growth structures through intercellular connections, demonstrating clear network expansion and collective growth characteristics (Fig. 4F). Confocal 3D reconstruction images further showed that adjacent microspheres were interconnected via numerous Tuj-1-positive cellular structures, forming a spatially continuous network system (Fig. 4G). This ability to form spatially continuous, inter-microsphere networks suggests that the porous architecture provides not just a surface for adhesion, but a functional scaffold for tissue-level integration.

To elucidate the molecular mechanisms driving these morphological enhancements, we performed whole-transcriptome RNA sequencing comparing PM@NSC-3D with NSC-2D. Principal Component Analysis (PCA) revealed distinct separation and clustering of the PM@NSC-3D and NSC-2D groups along the Principal Component 1 (PC1) and Principal Component 2 (PC2) dimensions (Fig. 4H). This indicates that the PDLLA porous microsphere-based 3D culture system significantly altered the global transcriptomic profile of NSCs, resulting in markedly different gene expression patterns between the two groups. Volcano plot analysis identified a total of 3982 DEGs (|log2FC|≥1 & q < 0.05)) between the PM@NSC-3D and NSC-2D groups, including 1973 significantly upregulated and 2009 significantly downregulated genes (Fig. 4I). These results suggest that the 3D culture system broadly regulates the gene expression network of NSCs, providing a transcriptional basis for the observed changes in their biological properties. Based on the DEG dataset, and by integrating literature reports on key genes regulating NSC fate with core genes from pathway enrichment analyses, we identified 59 representative core DEGs highly associated with 3D culture. These genes are involved in crucial biological processes, including the positive regulation of angiogenesis (Vegfa, Adm, Tgfbr2, Serpine1, Kdr, Flt1, Fgf2, Eng, Fgf18, Hmox1), basement membrane (Col4a1, Col4a2, Lamb1, Itgb4), cell adhesion (Itga5, Vcl, Icam1, Cav1), synapse (Ntn1, Nectin1, Cbln4, Arc, Nptxr, Ephb2), positive regulation of cell population proliferation (Sox9, Timp1, Pdgfb, Igf1r, Pdgfra), axon guidance (Unc5b, Gbx2), central nervous system development (Ngfr, Gsx2, Slc2a1, Slc1a1), neuron differentiation (Phox2a, Rest, Bmp4, Prdm1, Wnt3), astrocyte differentiation (Shh, Nog), inflammatory response (Tnc, Dmd, Mdk, Bmpr1b, Irgm, Ggt1, Sucnr1, Il18, Tlr4, Tbxa2r, Alox5), and neuron apoptotic process (Ascl1, Tgfb2, Cdk5, Map3k5, Chl1, Nrbp2). This broad transcriptional shift suggests that the 3D microenvironment pre-conditions NSCs towards a more regenerative and resilient phenotype (Fig. 4K). To elucidate the functional implications of these core DEGs, GO functional enrichment analysis indicated that they were primarily enriched in biological processes and functional categories such as positive regulation of angiogenesis, cell adhesion, positive regulation of cell population proliferation, synapse, focal adhesion, extracellular matrix organization, negative regulation of inflammatory response, neuron differentiation, axon guidance, negative regulation of neuron apoptotic process, and cell junction (Fig. 4J). Building on the functional classification, KEGG pathway analysis allowed us to pinpoint the specific signaling networks responsible for this shift. The results showed that the DEGs were enriched in ECM-receptor interaction, the HIF-1 signaling pathway, the Calcium signaling pathway, the PI3K-Akt signaling pathway, Focal adhesion, Neuroactive Ligand-receptor interaction, and Signaling pathways regulating pluripotency of stem cells (Fig. 4L). Among these, the marked enrichment of the HIF-1 signaling pathway—a master regulator of cellular adaptation to microenvironments—stood out as a potential primary candidate mechanism through which the 3D culture system modulates NSC fate, thereby providing a focused trajectory for subsequent in-depth investigation. Based on the GO and KEGG enrichment results, we further focused on two core candidate pathways: “positive regulation of angiogenesis” and the “HIF-1 signaling pathway.” GSEA was employed for validation to identify the core pathways governing NSC fate in the 3D culture system. The results demonstrated significant positive enrichment of “positive regulation of angiogenesis” (NES = 2.1797972; FDR = 0.0002377551; P = 0) and the “HIF-1 signaling pathway” (NES = 2.2845817; FDR = 0; P = 0) in the PM@NSC-3D group (Fig. 4M). These findings suggest that the 3D porous structure creates a unique microenvironment that activates the HIF-1 axis, thereby enhancing the proangiogenic and survival potential of the loaded NSCs. To further corroborate the transcriptomic findings, we examined the protein expression levels of the HIF-1 signaling pathway core molecule, HIF-1α, and the key angiogenic factor, VEGFA, using Western blot. The results showed that the protein expression levels of both HIF-1α and VEGFA were significantly higher in the PM@NSC-3D group compared to the NSC-2D group (Fig. 4N and O). This upregulation aligns with our sequencing data, confirming that the 3D system actively promotes a pro-angiogenic phenotype via the HIF-1 signaling pathway. Finally, having validated the superior structural and molecular properties of the PM@NSC 3D system, we sought to integrate it with the immunomodulatory BMH@Exo component to create a comprehensive therapeutic platform. We then verified the regulatory function of this integrated platform on NSC differentiation fate under 3D culture conditions. Specifically, immunofluorescence staining was performed to detect the expression of Nestin, Tuj-1, and GFAP. The neuronal differentiation efficiency was quantified using the Tuj-1/GFAP fluorescence intensity ratio. The results showed that the ratios in the Control and BMH groups were low, with no significant difference between them. In contrast, the ratios in the Exo and Exo + BMH groups were significantly higher than those in the former two groups (Fig. 4P and Q). This confirms that the composite (BMH@Exo-PM) combines the physical advantages of 3D scaffolding with the biological potency of exosomes, maximizing the potential for neuronal differentiation even in complex environments.

### 2.5 BMH@Exo reprograms the inflammatory microenvironment

**Fig. 5:**
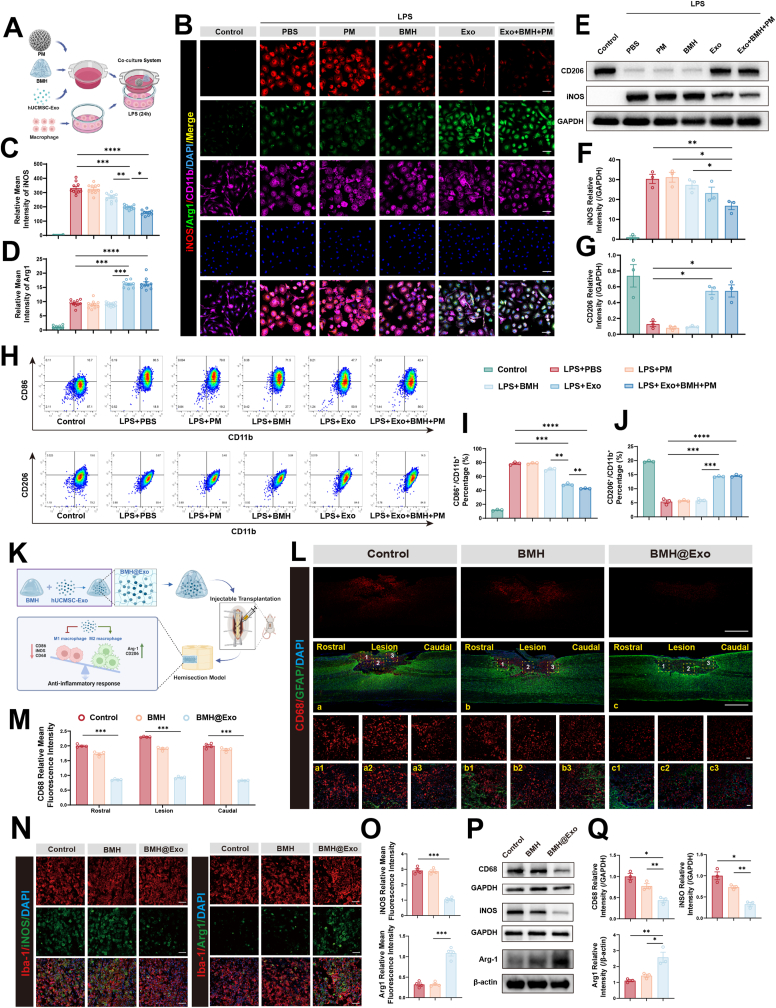
Composites attenuated the inflammatory microenvironment in vitro and in vivo. A) Schematic of the interactive system showing the interaction between the composite and BMDMs stimulated with LPS. B) Representative confocal images of BMDMs treated with different groups. BMDMs were stained with iNOS (red), Arg1 (green), CD11b (purple), and DAPI (blue). Scale bar: 50 μm. C-D) Quantitative analysis of iNOS (C) and Arg1 (D)mean fluorescence intensity (n = 10). E) Western blot bands of iNOS and CD206 expression in BMDMs. F-G) Quantitative analysis of iNOS/GAPDH (F) and CD206/GAPDH (G) ratio (n = 3). H) Flow cytometry showed reduced M1 (CD86^+^/CD11b^+^) and preserved M2 (CD206^+^/CD11b^+^) phenotypes in LPS-stimulated BMDMs treated with a composite. I-J) Quantitative analysis of the percentage of CD86^+^/CD11b^+^ cells and CD206^+^CD11b^+^ cells (n = 3). K) Schematic diagram illustrating the preparation, transplantation, and anti-inflammatory mechanism of the BMH@Exo composite in the spinal cord hemisection model. L) Representative confocal images of CD68 (red) and GFAP (green) in each group at the injured site 7 days after SCI. Scale bar: 1000 μm. Areas 1, 2, and 3 represent immunofluorescence images of the rostral, lesion, and caudal regions of the spinal cord. Scale bar: 50 μm M) Quantitative analysis of CD68 means fluorescence intensity in rostral, lesion, and caudal regions of the spinal cord (n = 3). N) Representative confocal images of iNOS (green, M1 marker) and Arg1 (green, M2 marker) expression of macrophage/microglia (Iba-1, red) 7 days after SCI. Scale bar: 50 μm. O) Quantitative analysis of Inos nd Arg1 mean fluorescence intensity (n = 4). P) Western blot bands of CD68, iNOS, and Arg1 expression in each group. Q) Quantitative analysis of CD68/GAPDH, iNOS/GAPDH, and Arg1/β-actin ratios in each group (n = 3). All data are presented as the mean ± SEM. ∗p < 0.05, ∗∗p < 0.01, ∗∗∗p < 0.001, ∗∗∗∗p < 0.0001.

Since the post-injury microenvironment is characterized by severe inflammation, which hinders regeneration, we first established an in vitro Transwell system to evaluate if our composite could modulate macrophage behavior (Fig. 5A). This study focused on bone marrow-derived macrophages (BMDMs) to investigate the effects of the composite and its components on macrophage polarization in vitro. Primary macrophages were first extracted from the femurs of rats and matured using M-CSF induction for 7 days. Subsequently, an inflammation model of BMDMs was established by activating the cells with LPS. Activated M1-type BMDMs exhibited shortened protrusions, enlarged cell bodies, and amoeboid morphology. Immunofluorescence analysis demonstrated that, relative to the LPS-treated control group, both the Exo group and the PM + BMH + Exo group exhibited a marked decrease in average iNOS fluorescence intensity and an increase in Arg1 fluorescence intensity. The BMH group also showed a slight reduction in iNOS intensity (Fig. 5B, C, D). Flow cytometry results demonstrated that the Exo group and PM + BMH + Exo group exhibited a significant decrease in the proportion of CD86+/CD11b + cells and a significant increase in CD206+/CD11b + cells. The BMH group also showed a reduction in CD86+/CD11b + cells (Fig. 5H, I, J). These results demonstrate that the exosome-containing component modulates inflammatory responses by suppressing macrophage polarization toward the M1 phenotype and enhancing polarization toward the M2 phenotype. This confirms that the composite acts as an immunomodulatory switch, actively reverting the pro-inflammatory state largely driven by the bioactive cargo of hUCMSC-Exo. Western blotting revealed comparable results, further confirming that the component reduces iNOS protein expression in activated macrophages under inflammatory conditions and promotes CD206 protein expression (Fig. 5E, F, G). To ensure this immunomodulatory effect extends to the central nervous system's resident immune cells, we replicated the assay using the microglial cell line BV2. Mirroring the observations in BMDMs, immunofluorescence staining confirmed that the composite exerts a similar immunomodulatory effect on microglia. When compared to the LPS-treated control, both the Exo and Exo + BMH + PM groups had significantly diminished iNOS fluorescence area and enhanced Arg1 fluorescence area. Conversely, the PM group showed negligible change (Fig. S19). These parallel findings confirm that the therapeutic efficacy covers both infiltrating peripheral macrophages and resident microglia, ensuring comprehensive immune regulation.

To quantify the downstream consequences of this polarization shift, we detected the mRNA expression levels of the pro-inflammatory cytokines tumor necrosis factor-α (TNF-α) and interleukin-1β (IL-1β) in BMDMs via quantitative real-time polymerase chain reaction (RT-qPCR). The experimental findings indicated that mRNA expression levels of TNF-α and IL-1β in the BMH + Exo + PM treatment group were markedly reduced compared to those observed in the control group. These results provide further confirmation of the underlying mechanism by which this system enhances SCI repair through effectively dampening the cytokine storm (Fig. S20). Crucially, the ultimate test of microenvironmental remodeling is whether it can protect vulnerable stem cells. Therefore, we verified if the system could maintain its neurogenic guidance under inflammatory stress. Firstly, BMDMs were stimulated with 1000 ng/mL LPS for 24 h to induce their activation. Subsequently, a Transwell co-culture system was established by co-incubating the activated BMDMs with NSCs treated under different conditions, with BMDMs seeded in the upper chamber and NSCs in the lower chamber. After 7 days of co-culture, the expression profiles of the neuron-specific marker Tuj-1 and the astrocyte marker GFAP were detected via immunofluorescence staining. The results showed that even under inflammatory stress, the differentiation ratio of NSCs toward the neuronal lineage in the BMH@Exo-PM group was significantly higher than that in the control group, demonstrating that the system can still effectively induce NSCs to differentiate into neurons under inflammatory stress conditions (Fig. S21). This demonstrates that the system creates a “safe haven” enabling NSCs to maintain their differentiation potential even amidst a hostile inflammatory milieu.

Having validated the potent anti-inflammatory effects in vitro, we proceeded to verify if BMH@Exo could remodel the hostile microenvironment in the acute phase of rat SCI. hUCMSC-Exo was loaded into BMH to form a BMH@Exo component, which was delivered via injection to the hemisected SCI site in rats to verify its anti-inflammatory effects (Fig. 5K). On the seventh day after SCI, samples were collected, and the level of inflammatory infiltration at the injury site was observed through immunofluorescence staining. In the injury control group, CD68-positive inflammatory cells at the injury site showed a prominent level of infiltration. After BMH injection, the number of inflammatory cells at the injury site slightly decreased, but the difference was not statistically significant. However, when BMH loaded with hUCMSC-Exo was injected, the number of inflammatory cell infiltrations at the injury site was significantly reduced (Fig. 5L and M). To investigate the effect of the BMH@Exo component on macrophage/microglial polarization at the injury site, immunofluorescence staining was used to observe their polarization state. Results showed that compared to the control group, the BMH + Exo group had significantly reduced iNOS fluorescence intensity and increased Arg1 fluorescence intensity in Iba-1-positive cells (macrophages/microglia) at the injury center (Fig. 5N and O). Western blot analysis further confirmed these findings, showing that the BMH@Exo component reduced CD68 and iNOS expression, and increased Arg1 expression at the injury center (Fig. 5P and Q). Collectively, these in vivo data demonstrate that the BMH@Exo component effectively suppresses early-stage inflammation and reprograms macrophage polarization. This orchestration of a permissive microenvironment is the prerequisite for the successful neuroregeneration observed in our subsequent long-term studies.

### 2.6 Implantation of composite delivery system (BMH@Exo-PM@NSC) enhanced neurogenesis, axonal regeneration, and remyelination in vivo

**Fig. 6:**
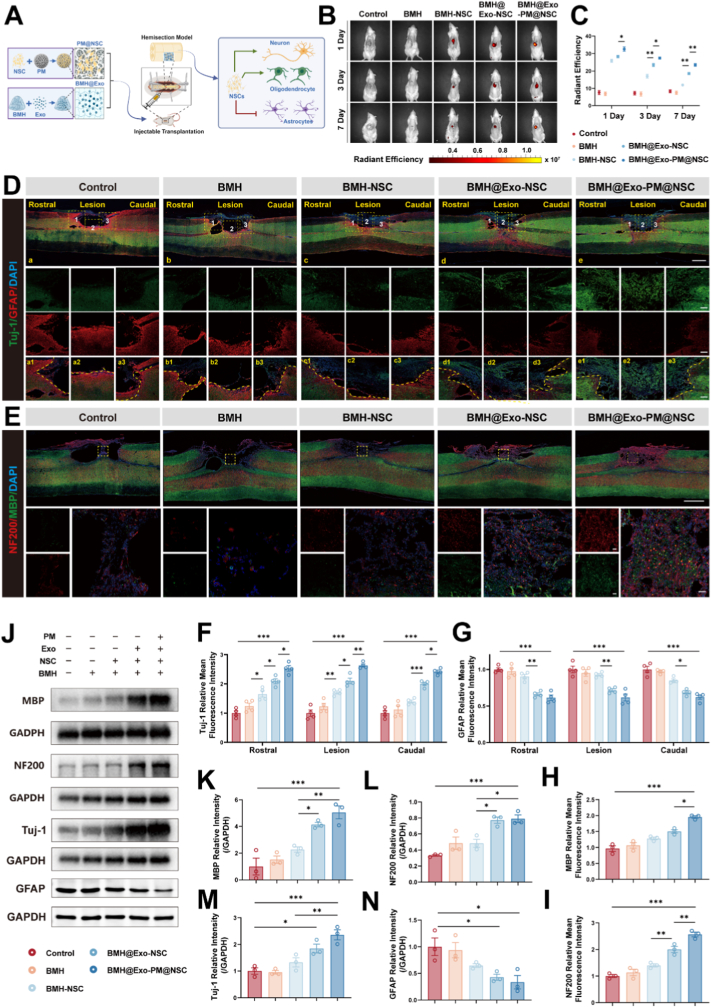
Implantation of the Composite Delivery System promoted axonal regeneration and remyelination 56 days after SCI. A) Schematic representation of the injectable transplantation of composite delivery system, illustrating the promotion of neuronal and oligodendrocyte differentiation while reducing astrocytic activation. B) In vivo fluorescence imaging of Cy7-labeled NSCs at 1, 3, and 7 days post-transplantation, showing sustained localization at the lesion site in the BMH@Exo-PM@NSC group. C) Quantitative analysis of radiant efficiency from fluorescence imaging in each group (n = 5). D) Representative confocal images of Tuj-1 (green) and GFAP (red) in each group at the injured site 56 days after SCI. Scale bar: 1000 μm. Areas 1, 2, and 3 represent immunofluorescence images of the rostral, lesion, and caudal regions of the spinal cord. Scale bar: 200 μm E) Representative confocal images of NF200 (red) and MBP (green) in each group at the injured site 56 days after SCI. Scale bar: 1000 μm. Scale bars of magnified image: 50 μm. F-G) Quantitative analysis of Tuj-1 (F) and GFAP (G) mean fluorescence intensities in rostral, lesion, and caudal regions of the spinal cord (n = 4). H-I) Quantitative analysis of MBP (H) and NF200 (I) mean fluorescence intensity in each group (n = 3). J) Western blot bands of MBP, NF200, Tuj-1, and GFAP expression in each group. K-N) Quantitative analysis of MBP/GAPDH (K), NF200/GAPDH (L), Tuj-1/GAPDH (M), and GFAP/GAPDH (N) ratios in each group (n = 3). All data are presented as the mean ± SEM. ∗p < 0.05, ∗∗p < 0.01, ∗∗∗p < 0.001.

Encouraged by the potent anti-inflammatory and neurogenic effects observed in vitro, we proceeded to evaluate the therapeutic potential of the composite delivery system in a rat model of spinal cord hemisection (Fig. 6A). First, in vivo imaging was used to evaluate the survival of transplanted NSCs in separate groups and at various time points. The results demonstrated that, across all time points, both the injury control group and the BMH group exhibited minimal fluorescence signals, consistent with the absence of transplanted cells. In contrast, the BMH + NSCs group displayed a marked reduction in fluorescence signals on days 1, 3, and 7, indicating decreased cell viability.

This rapid signal loss suggests that NSCs transplanted alone are vulnerable to the harsh post-injury microenvironment, leading to partial apoptosis or immune-mediated clearance. The BMH@Exo-NSC group demonstrated notably improved cell survival, exhibiting a less pronounced reduction in fluorescence signals during treatment compared to the BMH-NSC group. In the BMH@Exo-PM@NSC group, fluorescence signals did not significantly decrease on days 1, 3, and 7, significantly exceeding those of the other transplantation groups (Fig. 6B and C). This superior retention demonstrates that the composite delivery system effectively shields transplanted NSCs from the harsh host environment during the acute phase, addressing a major bottleneck in cell therapy. To determine if this enhanced survival translated into long-term structural repair, we performed histological analysis at 8 weeks post-injury. Immunofluorescence results showed that in the injury control group and BMH group, the average fluorescence intensity of Tuj1 was weak. Conversely, GFAP intensity was notably elevated, suggesting limited neuronal repair and marked astrocyte activation. Across the groups, from BMH-NSC to BMH@Exo-PM@NSC, the mean fluorescence intensity of Tuj1 demonstrated a progressive increase, peaking in the BMH@Exo-PM@NSC group. Simultaneously, GFAP levels gradually decreased across treatment groups, with significant reductions in astrocyte activation, particularly in the BMH@Exo-NSC and BMH@Exo-PM@NSC groups. This dual effect—promoting neurogenesis while suppressing astrogliosis—indicates that the composite successfully reshapes the regenerative niche. Notably, the BMH@Exo-PM@NSC group provided a 3D carrier for NSCs through porous microspheres, resulting in an increased number of Tuj1-positive cells, indicating that porous microspheres can function as a 3D scaffold to promote greater NSC differentiation into neurons (Fig. 6D–F, G). Western blot results showed similar findings, further validating that the composite delivery system promoted NSC differentiation into neurons at the injury site and reduced their tendency to differentiate into astrocytes (Fig. 6J–M, N). Beyond neuronal differentiation, the re-establishment of neural circuitry requires the regeneration of axons and their subsequent remyelination. From the BMH group to the BMH@Exo-PM@NSC group, we observed a progressive increase in the mean fluorescence intensity of MBP and NF-200, with the highest levels observed in the BMH@Exo-PM@NSC group, indicating optimal myelin formation and neurofilament repair (Fig. 6E–H, I). Western blot results further validated the above conclusions (Fig. 6J, K, L). Furthermore, the BMH@Exo-PM@NSC group exhibited the highest expression of synaptic markers (PSD95 and SYN) at 56 days post-injury (Fig. S22), confirming that the system promotes synaptogenesis essential for network reconstruction. These data confirm that the composite system not only replaces lost neurons but also facilitates the structural rebuilding of myelinated nerve fibers, laying the physical foundation for functional recovery.

### 2.7 Implantation of the composite delivery system promotes motor functional recovery after SCI

**Fig. 7:**
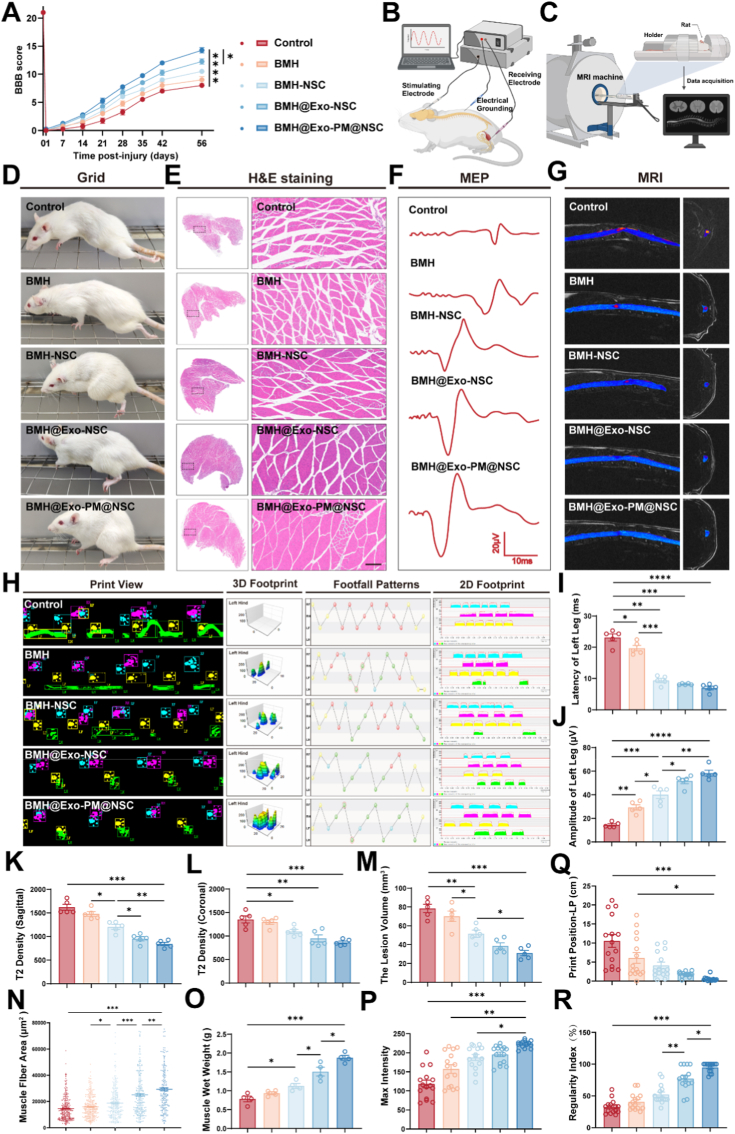
Evaluation of functional recovery and structural repair 56 days after SCI. A) The BBB score of spinal cord hemisection rats treated with several different methods (n = 5). B) Schematic illustration of the MEP recording system for evaluating motor signal conduction. C) Schematic illustration of the MRI scanning setup used to assess structural recovery in spinal cord lesions. D) Representative photos of rats crawling on the grid, showing the recovery of left hindlimb function in each group 8 weeks after SCI. E) H&E staining of gastrocnemius muscles indicated variations in muscle fiber morphology among the groups 56 days after SCI. Scale bar: 200 μm F) MEP results show variations in latency and amplitude, reflecting differences in motor signal conduction in the left hind leg of each group 56 days after SCI. G) Sagittal and axial T2-weighted MRI images of rats in each group 56 days after SCI. H) Footprint analysis with print views, footfall patterns, 3D footprints, and 2D footprints revealing differences in gait patterns among distinct groups. I, J) Quantitative analysis of MEP latency and amplitude in the left hind leg in each group (n = 5). K, L) Quantitative analysis of T2 density in sagittal and coronal planes, highlighting structural differences in spinal cord lesions among groups (n = 5). M) Quantitative analysis of lesion volume of the spinal cord in each group (n = 5). N) Quantitative analysis of gastrocnemius muscle fiber area in each group (n = 5). O) Quantitative analysis of wet weight of hind limb gastrocnemius muscles indicating muscle atrophy levels in each group (n = 4). P, Q, R) Quantitative analysis of catwalk at day 56 post-injury, including maximum footprint intensity, footprint positioning, and regularity index of left hindlimb (n = 15). All data are presented as the mean ± SEM. ∗p < 0.05, ∗∗p < 0.01, ∗∗∗p < 0.001, ∗∗∗∗p < 0.0001.

To quantitatively evaluate the functional restoration of the paralyzed limb, we first monitored the Basso, Beattie, and Bresnahan (BBB) scores over an 8-week period. After establishing the spinal cord hemisection model, rats immediately exhibited complete paralysis in the left hindlimb (BBB score of 0). In contrast, the motor function of the right hindlimb was unaffected, indicating successful modeling. Over several weeks of follow-up, the injury control group had the lowest BBB scores throughout the experiment, with the most limited recovery. The remaining groups demonstrated varying levels of improvement in BBB scores, with the BMH@Exo-PM@NSC group achieving the highest performance. This trajectory confirms that while spontaneous recovery is limited, the composite system actively accelerates motor function recovery (Fig. 7A and D). Since denervation leads to rapid muscle wasting, we investigated whether the neural repair observed histologically translated into the preservation of downstream muscle tissue. The gastrocnemius muscle wet weight in the injury control group was significantly lower than in other groups, indicating severe muscle atrophy caused by SCI. Compared to the control group, the gastrocnemius muscle wet weight increased in all other groups, with the BMH@Exo-PM@NSC group showing the highest wet weight (Figure S23, Fig. 7O). Histological H&E staining further confirmed this macroscopic observation. The results showed that gastrocnemius muscle fibers in the left hindlimb of rats in the injury control group exhibited significant atrophy with a markedly reduced average cross-sectional area. Some interfibrillar spaces were enlarged, and the morphology appeared irregular. In contrast, the extent of gastrocnemius muscle fiber atrophy was significantly reduced in the BMH@Exo-PM@NSC group, with a significant increase in average cross-sectional area. The gastrocnemius muscle fibers in this group were more tightly arranged, with reduced gaps between fibers and a more regular morphology. These results indicate that the restored neural innervation provided by the composite system successfully prevents neurogenic muscle atrophy, preserving the structural integrity required for locomotion (Fig. 7E–N). To complement the subjective BBB scores with objective gait parameters, we utilized the Catwalk gait analysis system on day 56. Results showed that all experimental groups experienced improvements in left hindlimb footprint characteristics, footprint intensity, and footfall distribution when compared to the injury control group. Notably, the BMH@Exo-PM@NSC cohort demonstrated the most significant recovery among all groups. Gait scores across all treatment groups exhibited comparable patterns in various parameters; notably, the BMH@Exo-PM@NSC group demonstrated significant improvements over the injury control group in Print Position, Max Intensity, and Regularity Index. These findings highlight that the treatment improves not just gross movement, but also complex motor coordination and weight-bearing capacity (Fig. 7H–P, Q, R, Fig S24). To verify the functional integrity of the neural pathways conducting signals across the injury site, neurophysiological assessments were conducted (Fig. 7B). The injury control group exhibited increased MEP latency and markedly reduced amplitude, indicative of substantial nerve conduction impairment. In contrast, all treatment groups demonstrated improvements in both amplitude and latency compared to controls. Notably, the BMH@Exo-PM@NSC group achieved the most favorable results, with significantly reduced MEP latency and amplitudes approaching baseline values (Fig. 7F–I, J). This restoration of signal conduction provides the electrophysiological basis for the observed motor recovery, confirming that the regenerated axons are functionally active. Finally, to visualize the macroscopic tissue repair non-invasively, MRI scans were performed 2 months post-injury (Fig. 7C). The results showed that spinal cord MRI in the injury-only group revealed the highest T2 signal density and defect volume in the coronal and sagittal views, indicating severe edema and tissue loss after SCI. The T2 signal density and defect volume gradually decreased in other groups, with the BMH@Exo-PM@NSC group showing the most significant reduction (Fig. 7K, L, M). This significant reduction in cavity volume confirms that the composite delivery system effectively bridges the lesion gap, reducing secondary tissue loss and providing a physical substrate for regeneration.

### 2.8 Engineered CTT platform circumvents adverse effects of NSC cryopreservation and transportation

**Fig. 8:**
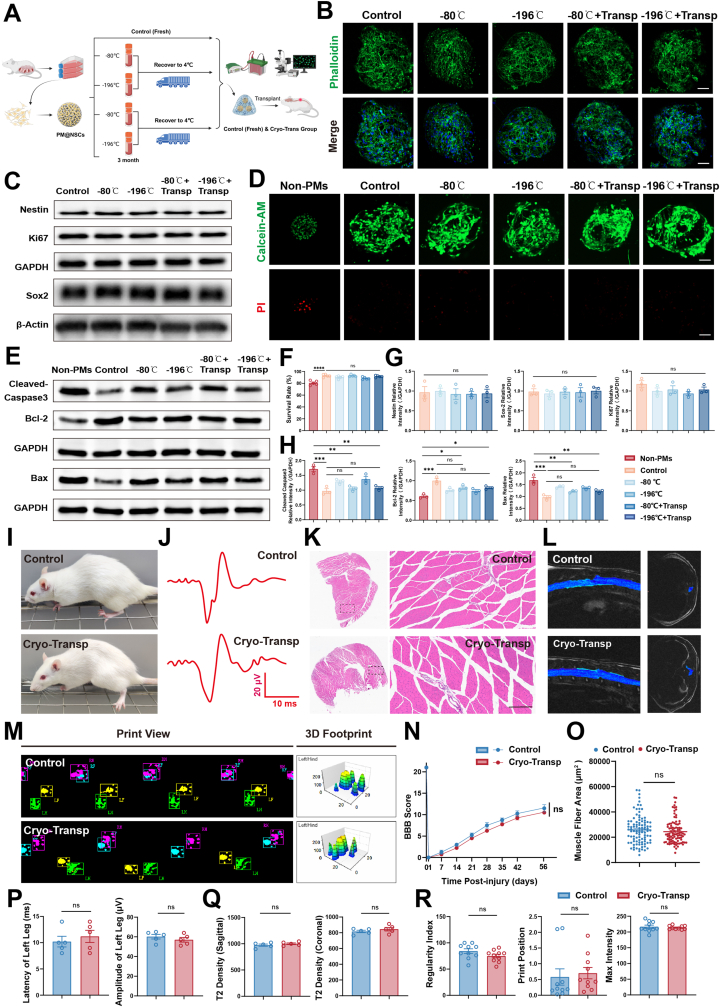
Comprehensive functional validation of CTT Platform after cryopreservation and simulated transport. A) Schematic illustration of the experimental workflow. Fresh or cryopreserved PM@NSC (at −80 °C or −196 °C for 3 months) were thawed and subjected to a 4-h simulated transport at 4 °C prior to in vitro analysis or in vivo transplantation for SCI repair. B) Representative confocal microscopy images assessing post-thaw cell cytoskeletal integrity of NSCs loaded onto PM. Phalloidin (green) for F-actin; DAPI (blue) for nucleus. Scale bar: 50 μm. C) Western blot bands of Nestin, Sox2, and Ki67 show no significant differences in protein expression levels among control (fresh), cryopreserved (−80 °C/-196 °C), and cryopreservation (−80 °C/-196 °C)-transport groups. D) Representative confocal microscopy images assessing post-thaw cell viability of NSCs loaded onto PM. Calcein AM (green) for live cells; PI (red) for dead cells. Scale bar: 50 μm. E) Western blot bands of Cleaved-Caspase3, Bcl-2, and Bax protein expression in protein expression levels among control (fresh), cryopreserved (−80 °C/-196 °C), and cryopreservation (−80 °C/-196 °C)-transport groups. F) Quantitative analysis of cell survival rate of NSC in each group (n = 5). G) Quantitative analysis of Nestin/GAPDH, Ki67/GAPDH, and Sox2/β-Actin ratios in each group (n = 3). H) Quantitative analysis of Cleaved-Caspase3/GAPDH, Bcl-2/GAPDH and Bax/GAPDH ratios in each group (n = 3). I) Representative photographs of rat hindlimb motor functions in each group, 8 weeks after SCI. J) MEP results show variations in latency and amplitude in the left hind leg of each group 56 days after SCI K) H&E staining of gastrocnemius muscles indicated variations in muscle fiber morphology among the groups 56 days after SCI. Scale bar: 200 μm L) Sagittal and axial T2-weighted MRI images of rats in each group 56 days after SCI. M) Footprint analysis with print views, footfall patterns, 3D footprints, and 2D footprints revealing differences in gait patterns among separate groups. N) BBB scores demonstrate comparable locomotor functional recovery across all groups, including the Control group and the Cryopreserved-Transport treated group (n = 5). O) Quantitative analysis of gastrocnemius muscle fiber cross-sectional area, indicating similar muscle functional recovery (n = 5). P) Quantitative analysis of MEP latency and amplitude in the left hind leg in each group (n = 5). Q) Quantitative analysis of T2 density in sagittal and coronal planes in spinal cord lesions among groups (n = 5). R) Quantitative footprint analysis on day 56 post-injury included the maximum footprint intensity, footprint positioning, and the regularity index of the left hindlimb (n = 10). All data are presented as the mean ± SEM. Statistical analysis showed no significant differences (n.s.) among the experimental groups. ∗p < 0.05, ∗∗p < 0.01, ∗∗∗p < 0.001.

A critical barrier to the wide-scale clinical application of stem cell therapy is the logistical challenge of storage and transportation. To address this, we developed an engineered CTT platform and sought to determine if it could effectively reduce cell damage typically associated with freeze-thaw cycles. Specifically, samples cryopreserved for three months were thawed and subjected to a simulated 4-h transportation at 4 °C. Specifically, samples cryopreserved for three months were thawed and subjected to a simulated 4-h transportation at 4 °C. The in vitro experiments were divided into five groups: control group (non-cryopreserved/non-transported), −196 °C cryopreservation group, −80 °C cryopreservation group, −196 °C cryopreservation + transportation group, and −80 °C cryopreservation + transportation group (Fig. 8A). Phalloidin staining demonstrated that cell morphology in both cryopreserved and transportation groups was comparable to that of the control group, indicating preservation of cytoskeletal integrity and function (Fig. 8B). Additionally, Western blot analysis showed no statistically significant differences in the expression levels of Nestin and Sox-2 (neural stem cell markers) or Ki67 (a proliferation marker) across all groups (Fig. 8C–G). These results confirm that the platform creates a robust physical shield that preserves cellular identity and proliferative potential even under extreme thermal stress. To quantify the specific protective benefit of microsphere anchorage compared to traditional cell suspension, we introduced a “non-PMs” control group (free NSC spheroids). Live/dead staining analysis demonstrated that the viable cell ratio in all NSC@PM cryopreservation groups, including the cryopreservation plus transportation subgroup, was not significantly different from the NSC@PM control group, and was notably higher than that observed in the Non-PM group. This stark contrast indicates that the “anchorage” provided by PMs is crucial for sustaining cell viability during the freeze-thaw process (Fig. 8D–F). To understand the molecular basis of this resilience, we revisited our sequencing data.

Sequencing analysis comparing PM@NSC-3D with NSC-2D indicated that PM@NSC markedly upregulated genes involved in the “negative regulation of neuron apoptotic process” (Fig. 4J). To further validate this mechanism at the protein level, we employed Western Blot analysis to detect the expression levels of pro-apoptotic proteins (cleaved-Caspase-3 and Bax) and the anti-apoptotic protein (Bcl-2) in NSCs from each group. The results showed that after cryopreservation and thawing, compared with the Non-PMs group, PM@NSC exhibited significantly downregulated expression of cleaved-Caspase3 and Bax, while Bcl-2 expression was significantly upregulated (Fig. 8E–H). This indicates that PM@NSC can remarkably enhance the anti-apoptotic and anti-stress capabilities of NSCs after freeze-thaw cycles. The criterion for a translatable product is whether the stored product performs therapeutically as well as the fresh one. To validate this, the cryopreserved-transported PM@NSC constructs were transplanted into the SCI rat model. Evaluation at 8 weeks post-transplantation revealed no significant differences between the cryopreservation-transportation group and the control group in terms of BBB scores, hindlimb grip strength, motor evoked potential (MEP) latency/amplitude, MRI of the spinal cord, gastrocnemius muscle fiber cross-sectional area, and Catwalk gait parameters (maximum footprint intensity, footprint positioning, and regularity index) (Fig. 8I–R). These results demonstrate that the CTT platform maintains full therapeutic potency after long-term storage and transportation. This “off-the-shelf” capability eliminates the need for on-site cell culture, significantly lowering the barrier for clinical translation.

## 3 Discussion

NSC transplantation for SCI has encountered significant challenges, such as limited cell survival, unpredictable differentiation pathways, and marked functional deterioration after cryopreservation and subsequent thawing. To address these critical bottlenecks, this study moved beyond single-modality treatments to develop an integrated CTT platform. The core of this platform is the BMH@Exo-PM composite. This carrier physically constructs a 3D biomimetic scaffold using microspheres while biologically integrating the immunomodulatory and neurotrophic functions of exosomes. This integrated approach enables comprehensive, closed-loop management, encompassing standardized in vitro storage and precise in vivo delivery. As a result, it substantially enhances transplantation protocols for exogenous neural stem cells and offers a systematic strategy to address the various challenges associated with spinal cord injury repair.

As a key functional component of the CTT platform, hUCMSC-Exo plays a dual role in SCI repair: anti-inflammation and neuroprotection [24,25]. However, in clinical practice, simple local injections often fail to maintain long-term effects due to the “washout effect.” To mitigate this, we constructed the “Exo-BMH” synergistic delivery composite. unlike Fan et al. who used conductive hydrogels to load exosomes for microglial polarization, our BMH carrier effectively improved the in vivo stability of exosomes. Furthermore, its biomimetic matrix and sustained-release capabilities enhanced the ability of hUCMSC-Exo to regulate the polarization balance of macrophages and microglia [26]. This “Exo-BMH” synergistic mode effectively regulated the disordered immune microenvironment following SCI. It reduced the expression of pro-inflammatory factors and elevated anti-inflammatory factors, thereby reshaping a suitable “immune-privileged” niche for the engraftment and survival of exogenous NSCs [27,28]. Beyond improving the immune microenvironment, the precise induction of NSC differentiation into functional neurons is critical for repair. We found that hUCMSC-Exo combined with PM not only supported NSC survival but also significantly promoted differentiation into the neuronal lineage, even under inflammatory stress. Transcriptome sequencing revealed the underlying mechanism: the composite activated the neuroactive ligand-receptor interaction pathway, simulating signals favorable to neural differentiation. Specifically, hUCMSC-Exo + PM regulated cell fate through the synergistic action of the PI3K-Akt signaling pathway and calcium signaling pathways. Western Blot results were consistent with the sequencing data. These findings align with current frontier research. Wang et al. found that piezoelectric nanomaterials promoted synapse maturation by activating voltage-gated calcium channels (VGCC) in NSCs [29], while Zhang et al. confirmed that magnetic nanoparticles directed NSC differentiation via the PI3K-Akt-mTOR pathway [30]. Our results suggest that the PI3K-Akt pathway primarily regulates the NSC survival microenvironment and differentiation direction, while calcium signaling focuses on neuronal maturation and the establishment of synaptic function. Together, they constitute the core regulatory network for targeted NSC differentiation. Importantly, the composite specifically induced enrichment of the cholinergic synapse pathway, which is closely related to motor function, and synergistically upregulated glutamatergic and GABAergic pathways. This provides a molecular basis for rebalancing network excitation and inhibition. The high expression of synaptic proteins observed in both in vitro and in vivo experiments further confirmed functionally that the composite effectively promotes NSC synapse formation and integration.

In addition to the microenvironmental regulation provided by BMH@Exo, PDLLA-PM served as the composite skeleton and a core element for the NSC 3D culture microenvironment. Together, they enabled precise regulation of the platform's microenvironment. Compared to similar existing studies, PDLLA-PM demonstrates distinct advantages. While the Lam-pGelMA microspheres prepared by Wang et al. enhanced adhesion via laminin, they tended to aggregate and lacked specific interventions for inflammation. Our “BMH-PM” system achieved uniform dispersion through self-assembly and innovatively introduced exosomes to form an “anti-inflammation + differentiation” loop. Although slightly less specialized regarding specific adhesion sites, the overall therapeutic strategy is more systematic [31,32]. Furthermore, while the DLP technology used by Qian et al. offers manufacturing precision and versatility, our study represents a “pathology-oriented functional composite” approach, focusing on deep intervention into SCI pathology [33]. The porous structure of PDLLA-PM not only supported excellent NSC alignment and viability but, crucially, allowed NSCs on the microspheres to extend Tuj1-positive neurites across physical gaps. This drove multiple “PM@NSC” units to self-organize into complex 3D neural network structures. This “cross-regional interconnection” overcomes the spatial limits of single microspheres and theoretically mimics the interconnectivity of in vivo neural tissue. This demonstrates the advantage of a “pathology-oriented” strategy over a purely “technology-enabled” strategy for deep tissue repair. In contrast to earlier research primarily addressing cell adhesion or proliferation [34], our whole-transcriptome sequencing analysis demonstrated that the PM@NSC-3D system robustly activates the HIF-1 signaling pathway, as well as other pathways that positively regulate angiogenesis. Subsequent Western blot analyses corroborated these findings at the protein level. Activation of the HIF-1 pathway accounts for the improved adaptability of neural stem cells (NSCs) to hypoxic conditions and indicates that the platform may facilitate angiogenesis within the injured region [[35], [36], [37]]. This provides a key mechanistic basis for the advantages of 3D culture strategies in ameliorating ischemic and hypoxic microenvironments.

The clinical translation of stem cell therapy relies heavily on cryopreservation technology, yet the loss of cell viability post-thaw remains a bottleneck limiting efficacy [38,39]. The CTT platform in this study significantly reduced ice crystal damage and DMSO toxicity during freezing through the physical protection of microspheres and the activation of anti-apoptotic pathways [40]. In vitro and in vivo validation proved that the platform maintained high NSC viability, proliferation, and anti-apoptotic capacity after recovery. This integrated strategy ensures the effective dosage of transplanted cells. By integrating cell extraction, 3D culture, frozen transport, and transplantation into a seamless workflow, it reduces risks of contamination and loss. This offers a feasible technical framework for standardized, scalable nerve regeneration therapies. Additionally, the favorable biosafety profile of all components lays a solid foundation for future clinical application.

Although this study preliminarily validates the effectiveness of the CTT system, several limitations require objective consideration. First, we employed a rat spinal cord hemisection model. While this model is highly reproducible for quantifying axonal regeneration and evaluating materials, its “clean cut” nature differs pathologically from the contusion or compression injuries common in the clinic, which involve severe disruption of the blood-spinal cord barrier and oxidative stress [[41], [42], [43]]. While both share core mechanisms such as glial scarring and inflammatory infiltration, and the hemisection model allows for self-control to reduce individual variance, future strategies must be verified in large animal contusion models that better mimic clinical reality [44]. Second, batch-to-batch variation in exosomes presents a hurdle for clinical translation. Differences in parental cell status and isolation techniques can lead to fluctuations in composition and function [45,46]. To ensure reproducibility, future research must strictly adhere to MISEV2023 guidelines to establish a standardized quality control system [47]. In conclusion, this study primarily assessed the reparative effectiveness of exogenous NSCs but did not employ lineage tracing techniques to accurately differentiate between the contributions of exogenous and endogenous NSCs. This will be a priority in subsequent research to map a more complete diagram of the repair mechanism.

## 4 Conclusion

In summary, our integrated “Cryopreservation-Thawing-Transplantation” platform, utilizing the PM-BMH@Exo composite, significantly enhances the therapeutic potential of NSCs for SCI repair. In vitro assays demonstrated that the composite maintains post-thaw NSC viability and facilitates fate commitment toward the neuronal lineage. Mechanistic studies revealed two functional modules: the hUCMSC-Exo component, which modulates the inflammatory and trophic microenvironment, and the PDLLA porous microsphere scaffold, which provides three-dimensional structural support. Specifically, exosomes effectively regulate the local inflammatory niche and activate critical NSC signaling pathways—including Calcium and PI3K-Akt signaling—to drive neuronal differentiation and synaptic maturation. Synergistically, the microsphere scaffold mimics the in vivo physiological milieu, establishing a biomimetic niche that supports NSC proliferation. Notably, NSCs seeded within the porous microspheres extended neurites across inter-microsphere gaps, facilitating the self-assembly of complex 3D neural networks. Furthermore, the PM@NSC-3D system activated HIF-1 and pro-angiogenic pathways, indicating a mechanism that improves local blood supply and host-graft integration under hypoxic conditions. In vivo evaluations in a rat hemisection model confirmed that the platform promoted neurological recovery and reduced tissue damage through three integrated mechanisms [1]: potent immunomodulation [2]; enhanced NSC engraftment; and [3] promotion of neurogenesis, axonal regeneration, and myelination. Crucially, the platform overcomes a major translational bottleneck by maintaining high stemness and viability even after prolonged cryopreservation, thereby achieving functional recovery comparable to fresh NSC transplantation. The pre-packaged, closed-loop workflow—integrating storage, transport, and delivery—minimizes contamination risks and viability loss. Collectively, this study establishes our CTT system as a robust, clinically compatible platform for SCI therapy. Future work will focus on elucidating the long-term integration mechanisms between the graft and host spinal cord tissue.

## 5 Experimental section

### 5.1 Preparation and hydrolysis modification of porous microsphere

Poly (DL-lactic acid) porous microspheres were fabricated using the water/oil/water double emulsion method. In brief, 280 mg of PDLLA was dissolved in 8 mL of dichloromethane (DCM), serving as the oil phase, while an NH4HCO3 aqueous solution served as the inner aqueous phase. Under ice water bath conditions, 5 mL of 15 % ammonium bicarbonate solution was emulsified into 8 mL of 3.5 % PDLLA solution using an ultrasonic dispersing device at a set power for 3 min to form a water-in-oil (W/O) emulsion. The prepared emulsion was then rapidly transferred into 300 mL of a 0.1 % (w/v) polyvinyl alcohol (PVA) solution and stirred at 650 rpm at room temperature for a minimum of 4 h, allowing the organic solvent to evaporate completely and the solid to solidify into spheres. After stirring, the microspheres were collected and washed several times with distilled water. Following collection, the microspheres were separated using standard sieves to obtain porous microspheres with diameters ranging from 150 to 300 μm. The porous microspheres were immersed in 0.1 M sodium hydroxide solution at room temperature for 30 min. Finally, the porous microspheres were washed 3 to 5 times with sterile water and lyophilized for further use.

### 5.2 Characterization of porous microsphere physical properties

#### 5.2.1 Particle and pore size analysis

The basic morphology of the microspheres was examined with an optical microscope. The vacuum freeze-dried microspheres were fixed to a conductive adhesive, gold-coated, and their microstructure was observed using a scanning electron microscope (SEM). The particle and pore sizes of the microspheres were measured using ImageJ software, and distribution graphs were plotted with Origin software.

#### 5.2.2 Measurement of contact angle

The contact angles of the microspheres before and after hydrolysis were measured using a VCA Optima dynamic contact angle measuring instrument. The experiment was conducted at room temperature, with 5 μL of distilled water dropped onto the sample surface. Data were recorded throughout the experiment.

#### 5.2.3 Fourier Transform Infrared Spectroscopy (FTIR)

Fourier Transform Infrared Spectroscopy (FTIR) was used to confirm that the PDLLA microspheres retained their structure after hydrolysis. The sample was prepared by pressing a potassium bromide pellet, with a wavenumber range of 4000–400 cm^−1^ and a resolution of 4 cm^−1^.

#### 5.2.4 Degradation properties

Degradation analysis of the porous microspheres was performed under simulated physiological pH and temperature conditions. Approximately 20 mg of PDLLA microspheres were placed in 10 mL of PBS solution (pH 7.4) and incubated at 37 °C. At 1, 2, 3, and 4 weeks, the microcarriers were washed with PBS, and the microscopic morphological changes of the microspheres during degradation were observed using an electron microscope.

### 5.3 Preparation of biomimetic matrix hydrogel

The Biomimetic Matrix Hydrogel (BMH) was purchased from the CulX series of Matrix (Tianjin) Biotechnology Co., Ltd., and samples were prepared according to the manufacturer's guidelines. The procedure involves mixing the lyophilized powder at the required concentration with complete cell culture medium (DMEM/F12, 10 % fetal bovine serum) or PBS by vortexing. The mixed solution is incubated at room temperature for 5–10 min to form a stable hydrogel.

### 5.4 Characterization of biomimetic matrix hydrogel physical properties

#### 5.4.1 Critical aggregation concentration detection

The critical aggregation concentration (CAC) was determined using Nile Red (Nile Red, 98 %, Heowns) as a solvent-based fluorescent probe. This method relies on the change in the fluorescence characteristics of Nile Red in a hydrophobic microenvironment, which indicates the aggregation behavior of the biomimetic matrix hydrogel. Prepare 2 mL of hydrogel solutions with concentrations of 75, 200, 300, and 400 μg/mL. A 1 mM Nile Red stock solution was prepared by dissolving Nile Red in ethanol, and 2 μL of this stock solution was added to each sample. In brief, the concentration of Nile Red in each sample was maintained at approximately 1 μM, while the concentration of the hydrogel solution was gradually increased. All samples were incubated at 37 °C for 30 min. Turn on the fluorescence spectrophotometer 30 min before measurement, selecting an excitation wavelength of 580 nm, an emission wavelength of 320–700 nm, and a voltage of 600 V for testing. Each sample is repeated three times, and the average fluorescence intensity is measured. The CAC was determined by the intersection of two linear fits from different concentration regions.

#### 5.4.2 The secondary structure of hydrogels

To characterize the secondary structure of the biomimetic matrix hydrogel, a Fourier transform infrared spectrometer (FTIR, Nicolet iS5, Tianjin, China) was used for analysis. The hydrogel samples (100 μL) were freeze-dried, and the test samples were prepared using the potassium bromide (KBr) pellet method. The wavenumber range for spectrum collection was from 1750 to 1450 cm^−1^, with a resolution of 4 cm^−1^. Each sample was scanned a total of 32 times, and a background spectrum was recorded prior to analysis. Finally, all infrared data were normalized using Origin software.

#### 5.4.3 Thioflavin T (THT) staining assay

ThT is a fluorescent dye that binds explicitly to β-sheet structures in amyloid fibers, making it widely used for qualitative and quantitative detection of these structures. Prepare 2 mL of aqueous solutions containing 0.3 %, 0.5 %, and 1.0 % hydrogels in advance. The solutions were incubated at 80 °C for 30 min and subsequently cooled to room temperature. Next, prepare a 1 mM ThT solution using ultra-pure water and mix it thoroughly with the hydrogel solutions at a volume ratio of 1:1000 (ThT solution to hydrogel solution). The mixtures were then incubated in a 37 °C oven for 30 min before testing. A quartz cuvette with a path length of 0.5 cm was used. Measurements were performed using a fluorescence spectrophotometer (Nicolet iS5, Thermo Fisher Scientific, Tianjin, China) with the excitation wavelength set to 450 nm, the emission range from 320 to 600 nm, and the voltage set to 700 V. Each sample group was measured in five replicates.

#### 5.4.4 Swelling properties

The swelling properties of the hydrogel scaffold were characterized using the volumetric method in PBS. Briefly, hydrogels of different concentrations were placed in 0.5 mL graduated sample vials and stabilized at room temperature. Then, 0.5 mL of PBS (37 °C, pH 7.4) was gently added along the sidewall of each vial. The hydrogel volumes were measured at predetermined intervals until no further increase was observed, indicating that equilibrium had been reached.

#### 5.4.5 Rheological properties

The mechanical properties of the Biomimetic Matrix Hydrogel were evaluated with a rheometer equipped with a 20 mm parallel plate (Mars 60, HAAKE, Germany). At the beginning of the experiment, the gap between the upper plate and sample stage was set to 30 mm, and measurements were taken when the plate was lowered to 0.5 mm. A 200 μL sample was loaded onto the stage with a pipette, and a thin layer of silicone oil was applied around the sample to prevent evaporation. The system was then equilibrated at 37 °C for 1800 s before testing. Time sweep curves for the storage modulus (G′) and loss modulus (G″) were measured at a fixed frequency of 1 Hz and an amplitude strain of γ = 1 %. Subsequently, a step-strain measurement was performed after the plateau in the storage modulus was reached in the time sweep process. After a 100 % strain was applied for 30 s, the hydrogels were left to recover for 15 min while measuring at 1 % strain (f = 1.0 Hz), during which the storage modulus returned to its original plateau. Such a measurement was repeated for three cycles.

#### 5.4.6 In vitro degradation

The biomimetic matrix hydrogel samples were incubated in PBS buffer (pH 7.4) and a 5 U/mL collagenase solution at 37 °C. At each predetermined time point, the old solution was removed and replaced with an equal volume of fresh PBS or preheated collagenase solution, respectively. Subsequently, the weight of the remaining hydrogel was measured. The degradation ratio was calculated using the following formula:

Degradationratio(%)=(Mo−Md)/Mo×100%

where Mo and Md represent the initial weight of the hydrogel and the weight after degradation, respectively.

#### 5.4.7 TEM analysis

Transmission electron microscopy (TEM) was utilized to analyze pre-incubated hydrogel samples (0.01 wt%). A 10 μL droplet of the hydrogel was deposited on a carbon-coated copper grid (300 mesh, Beijing XinXing BaiRui Technology Co., Ltd.) and air-dried. The sample was stained with 2 % phosphotungstic acid and incubated for 60 s. Excess water was removed from the grid using filter paper, and the sample was re-dried. Images were captured with a Talos G2 200X transmission electron microscope.

#### *5.4.8* SEM analysis

The nanofiber network of the BM hydrogel scaffold was observed with scanning electron microscopy (SEM). The hydrogel solution was frozen at −80 °C in an ultra-low-temperature freezer, rapidly frozen in liquid nitrogen for 15 min to minimize water crystallization, and then freeze-dried for 24 h using a vacuum freeze dryer. The freeze-dried sample was imaged with SEM (JSM-IT300LV) at an accelerating voltage of 10 kV.

### 5.5 Preparation and characterization of the biomimetic matrix hydrogel + PM composites

A biomimetic matrix hydrogel was combined with porous polylactic acid microspheres at mass ratios of 15:1, 10:1, 5:1, and 1:1 to prepare Matrix Hydrogel + PM composite scaffolds. The composite materials were then loaded into 2.5 mL EP tubes, and microsphere sedimentation was recorded at the following time points: 0, 10 min, 30 min, 12 h, 24 h, 72 h, and 120 h. Rheological measurements of Matrix Hydrogel + PM composite scaffolds at varying mass ratios were performed using a rheometer at 37 °C. A 20 mm plate was used to measure the storage modulus (G′) and loss modulus (G″), with a shear strain of 1 % and a shear frequency of 1 Hz.

### 5.6 Preparation and characterization of hUCMSCs

The hUCMSCs used in this study were purchased from Changhe Biotechnology Inc., Tianjin, China. The identification and functional verification of human umbilical cord mesenchymal stem cells (hUC-MSCs) are primarily accomplished through the phenotypic analysis of cell surface markers and the evaluation of their multipotent differentiation potential in vitro. For phenotypic identification via flow cytometry, cells were collected after trypsinization, first stained with Zombie UV™ Fixable Viability Dye to distinguish viable from non-viable cells and ensure analytical accuracy, then treated with blocking buffer to reduce non-specific binding, followed by incubation with fluorescent-conjugated antibodies against MSC positive markers (CD73, CD90, CD105) and negative markers (CD11b, CD19, CD34, CD45, HLA-DR) at 4 °C in the dark; after washing with buffer, cells were fixed in 1 % paraformaldehyde, detected using a BD LSRFortessa flow cytometer, and data were analyzed with FlowJo V10 software, thereby quantitatively verifying whether the cells conformed to internationally recognized MSC phenotypic criteria. The multipotent differentiation capacity of hUC-MSCs was confirmed via in vitro trilineage differentiation induction assays, where commercially available directed differentiation kits (Sigma-Aldrich, USA) were used to induce hUC-MSCs into osteogenic, adipogenic, and chondrogenic lineages, respectively: Alizarin Red S (ARS) staining was performed post-osteogenic induction, as ARS binds explicitly to calcium ions in cell-secreted calcium nodules to form orange-red precipitates, confirming the formation of mineralized matrix; Oil Red O staining was used for adipogenic differentiation, which stains explicitly intracellular accumulated neutral lipids red to indicate lipid droplet formation; Alcian Blue staining was conducted for chondrogenic differentiation under acidic conditions, and the dye binds to glycosaminoglycans (GAGs) enriched in the extracellular matrix to produce a blue color, verifying the successful synthesis of cartilaginous matrix. These three specific staining methods provided intuitive and specific validation of the osteogenic, adipogenic, and chondrogenic differentiation potentials of hUC-MSCs at the functional level.

### 5.7 Isolation and characterization of hUCMSC-Exo

Exosome-free Fetal Bovine Serum (FBS) was prepared with a high-speed centrifuge (Beckman, Germany). When the cell density in the five-layer culture flasks reached 80 %, the exosome-free medium was replaced with the standard culture medium. After three days of hUCMSC culture, the culture medium was collected and subjected to high-speed centrifugation to extract exosomes. The enriched exosomes were resuspended in PBS for further experiments. The morphology and structure of the exosomes were assessed using a high-resolution transmission electron microscope (TEM, Hitachi HT7700, Japan). The diameter distribution of the exosomes was measured with a nanoparticle tracking analyzer (Malvern, NS300, UK). Total protein was extracted from hUCMSC-Exos and hUCMSC, and surface protein markers were identified using Western blotting. The primary antibodies used included TSG101 (Abmart, China), CD63 (Abmart, China), CD9 (Abmart, China), and GAPDH (Proteintech, IL, USA).

### 5.8 Isolation and culture of NSCs

Under sterile conditions, embryos are obtained from Wistar female rats on days 14–16 of pregnancy. The cerebral cortex is then isolated and cut into small pieces. Subsequently, DNase I (Sigma, USA) and papain (Worthington Biochemical, USA) are used to digest the tissue at 37 °C for 10–15 min, resulting in a single-cell suspension. The NSCs are then transferred to a basic culture medium and incubated at 37 °C with 5 % CO2. The basic culture medium contains DMEM/F12 (Gibco, USA), 2 % B-27 supplement without vitamin A (Gibco, USA), 1 % Glutamine MAX™ (Gibco, USA), 20 ng/mL bFGF (Peprotech, USA), 20 ng/mL EGF (Peprotech, USA), and 1 % penicillin/streptomycin (Gibco, USA). Cells that have been cultured up to the 3rd to 5th generation are used for subsequent experiments.

### 5.9 NSCs differentiation

To investigate the effect of composites on NSC differentiation, we utilized the composites to treat NSCs in a Transwell system (Corning, USA) with the two compartments placed separately. First, we treated the NSC spheres with Accutase (Sigma, USA) to obtain single cells. Then, we seeded the cells at an appropriate density in the lower chamber of a 24-well plate that had been treated with poly-L-lysine (Sigma, USA) and Laminin (Sigma, USA), and cultured them with differentiation medium. The differentiation medium was formulated with the following components: DMEM/F12, 2 % B-27 supplement (with vitamin A), 1 % fetal bovine serum (FBS), 1 % Glutamine MAX™ (Gibco, USA), and 1 % penicillin/streptomycin. After the NSCs adhered to the lower chamber, we added the composites and their individual components to the upper chamber of the Transwells. These components included: 200 μL DMEM/F12 basal medium as a blank control, 200 μL BMH, 0.2 mg PM, 200 μg/mL Exo, and 200 μL of BMH containing PM (0.2 mg) + Exo (200 μg/mL). After seven days, the samples will be further analyzed using Western Blot and immunofluorescence.

### 5.10 Isolation and culture of bone marrow-derived macrophages (BMDMs)

Under sterile conditions, the femurs and tibias of male Wistar rats (130–150 g) were isolated. PBS was used to wash the marrow, and a single-cell suspension was generated by filtering it through a cell filter. The collected cells underwent erythrocyte lysis and were then transferred to the induction medium for culture at 37 °C with 5 % CO2. The induction medium consists of DMEM (Gibco, USA), 10 % FBS (Gibco, USA), 10 % penicillin/streptomycin (Gibco, USA), and 20 ng/mL M-CSF (Sigma-Aldrich, USA). After 7 days of induction, BMDMs can be used for subsequent in vitro experiments.

### 5.11 Bone marrow-derived macrophages (BMDMs) polarization

To investigate the effects of the composite material on the polarization of BMDMs, the composites and BMDMs were treated in a Transwell interactive system (Corning, USA). Cells were seeded in the lower chamber of 24-well plates at an appropriate density, while the composites and their components were added to the upper chamber of the transwell. Lipopolysaccharide (LPS, 1000 ng/mL, Sigma-Aldrich) was added to the medium to induce an inflammatory response. Following 24 h of treatment, the samples underwent Western blotting, flow cytometry, and immunofluorescence analysis. The experimental groups were as follows: 200 μL of DMEM basal medium (blank control, without LPS), 200 μL of DMEM basal medium (positive control), 200 μL of BMH, 0.2 mg PM, 3 μg/mL Exo, and 200 μL of BMH combined with 0.2 mg PM and 3 μg/mL Exo.

### 5.12 Biocompatibility

#### *5.12.1* CCK-8 cell viability assay

NSCs were treated with composite materials at various time points and assessed for viability using the CCK-8 assay kit (Dojindo, Japan). Cell viability and proliferation were quantified by measuring absorbance (OD value) at 450 nm using a microplate reader.

#### 5.12.2 Live/dead cell staining

After three days of co-culturing, NSCs were stained with Calcein-AM/PI dual-staining reagents (Beyotime, China). The cells were incubated at 37 °C in the dark for 30 min. Following staining, images were captured using a confocal fluorescence microscope. Viable cells exhibited green fluorescence in the cytoplasm (Calcein-AM staining), while dead cells showed red fluorescence in the nuclei (PI staining).

#### *5.12.3* Cytoskeleton staining

The distribution of the cytoskeleton in 3D-cultured NSCs on microspheres was investigated by staining F-actin with Phalloidin conjugated to Alexa Fluor 488 (UElandy, China). The cells were fixed with 4 % paraformaldehyde and incubated at 37 °C in the dark for 30 min. The cytoskeletal structure was visualized using a confocal fluorescence microscope.

### 5.13 Immunofluorescence staining

Cells cultured on confocal dishes were fixed, permeabilized, and blocked. After overnight incubation with the primary antibody at 4 °C, the secondary antibody was added and incubated at room temperature for 1 h. The samples were imaged using a ZEISS LSM 900 confocal microscope (Germany). For spinal cord tissues, rats were perfused with 4 % paraformaldehyde (PFA), and the spinal cords were collected and incubated overnight in 4 % PFA. The spinal cord tissues were dehydrated in a gradient of sucrose solutions, and 10 μm-thick frozen sections were prepared. The sections were permeabilized (0.25 % Triton X-100) and blocked (5 % BSA), incubated overnight with the primary antibody at 4 °C, and then incubated with the secondary antibody at room temperature for 1 h. The sections were mounted with DAPI-containing mounting medium (Abcam, Cambridge, UK) and imaged using a ZEISS LSM 900 confocal microscope (Germany). The primary antibodies used include Tuj-1 (Abcam, Cambridge, UK), GFAP (Cell Signaling Technology, USA), Iba-1 (Abcam, Cambridge, UK), iNOS (Abcam, Cambridge, UK), GFAP (Abcam, Cambridge, UK), CD68 (Abcam, Cambridge, UK), NF-200 (Invitrogen, CA, USA), MBP (Abcam, Cambridge, UK), CD11b (Abcam, Cambridge, UK), Arg-1 (Abcam, Cambridge, UK),

PSD-95 (Abcam, Cambridge, UK), Synaptophysin (Abcam, Cambridge, UK), Nestin (Abcam, Cambridge, UK) and SOX-2 (Abcam, Cambridge, UK). The secondary antibodies used are IgG H&L Alexa Fluor 488 (Abcam, MA, USA), IgG H&L Cy3 (Abcam, MA, USA), IgG H&L Alexa Fluor 594, and IgG H&L Alexa Fluor 647 (Abcam, MA, USA).

### 5.14 Hematoxylin and eosin (H&E) staining

Muscle tissue samples were fixed overnight in a 4 % paraformaldehyde solution, then dehydrated using graded ethanol and cleared with xylene. The tissue was subsequently embedded in paraffin and sectioned into 5 μm-thick slices. The sections were deparaffinized, rehydrated through a graded ethanol series, and stained with hematoxylin and eosin (1 %). After dehydration with absolute ethanol and clearing in xylene, the sections were mounted.

### 5.15 Flow cytometry

After collecting BMDMs from different treatment groups, the cells were subjected to blocking. Cells were incubated at 4 °C in the dark with CD11b antibody (BioLegend, USA) and CD86 antibody (BioLegend, USA). Subsequently, cells were permeabilized using the Cyto-Fast™ Fix/Perm Buffer Set (BioLegend, USA) and then incubated at room temperature in the dark with CD206 antibody (Santa Cruz, USA). After washing away excess antibodies, the cells were incubated with the secondary antibody Alexa Fluor®647/APC (Abcam, MA, USA). The cells were fixed with 1 % paraformaldehyde and then analyzed. Data were collected using the BD LSRFortessa Cell Analyzer (BD Biosciences, USA) and analyzed with FlowJo 10.8.1 software.

### 5.16 Magnetic resonance imaging (MRI)

Magnetic resonance imaging (MRI) was performed 8 weeks after SCI in rats using a 3.0 T spectrometer (Discovery MR750, GE Healthcare, USA). Rats were anesthetized and positioned in a supine orientation within the radio frequency coil. Fast relaxation fast spin echo (FRFSE) pulse sequences were used to acquire sagittal and coronal T2-weighted images of the spinal cord. Specifically, the parameters for T2-weighted imaging were as follows: repetition time/echo time (TR/TE) = 3000/110 msec, image matrix = 320 × 224, field of view (FOV) = 6 mm.

### 5.17 Animals

This study utilized adult female Wistar rats (weight 200–230 g, n = 300) provided by Beijing Vital River Laboratory Animal Technology Co., Ltd. (Beijing, China, Permission Number: SCXK (Jing)-2016-0011). They were kept under SPF conditions in a controlled environment with a 12-h light/dark cycle, a temperature range of 22–24 °C, and 60–80 % humidity. All animal experiments were approved by the Tianjin Medical University General Hospital Experimental Animal Welfare Ethics Committee (Tianjin, China, Approval Number: IRB2024-DW-44). In the acute phase, the experimental animals were randomly divided into the following groups: control, BMH, and BMH@Exo. In the chronic phase, the experimental animals were randomly divided into the following groups: Control, BMH, BMH-NSC, BMH@Exo-NSC, and BMH@Exo-PM@NSC.

### 5.18 Spinal cord hemisection model

Under the assistance of a multichannel small-animal anesthesia machine (RWD Life Sciences Co., Ltd., China), each rat was anesthetized by inhalation of isoflurane (5 %). A longitudinal incision was made along the midline of the back under sterile conditions to expose the spinal cord through a dorsal laminectomy above the T10 vertebra. A 2 mm long longitudinal left-side hemisection was performed at the T10 thoracic level. Then, the composite was transplanted into the lesion site, and the muscles and skin at the incision were sutured. The rats' bladders were manually emptied twice daily until spontaneous urination was restored.

### 5.19 Behavioral assessment

We investigated whether the CTT platform enhances left hind-limb locomotor performance by conducting weekly assessments over eight weeks using the 21-point Basso–Beattie–Bresnahan (BBB) scoring system after SCI. Two observers who had no involvement in the surgical procedures related to SCI and were unaware of the animal model group assignments conducted these evaluations. The experiment took place in an open-field setting, where the observers recorded and analyzed hind-limb locomotor performance on video. A BBB score of 0 indicates complete paralysis of the left hind limbs, whereas a score of 21 signifies normal function.

### 5.20 Cat-walk gait analysis

The CatWalk XT automatic gait analysis system (version 10.6, Noldus IT, The Netherlands) is a comprehensive system for quantitatively evaluating the footsteps and movements of rats. This system employs real-time high-resolution cameras and footprint light refraction technology to detect subtle changes in size, position, and timing during movement. We conducted CatWalk gait analysis eight weeks after SCI surgery. Prior to the test, the rats were trained to walk from left to right in a dark environment without stopping or turning around. The software, CatWalk XT 10.6, serves as a reliable tool for evaluating gait after SCI. We collected relevant experimental data, with a specific focus on the gait parameters of the left hindlimb, and conducted subsequent targeted analysis based on the data from this limb.

### 5.21 Neuro-electrophysiological analysis

Neuro-electrophysiological examination detects the electrical signals and conductivity of the spinal cord using an electrophysiological instrument (YRKJ-G2008; Zhuhai Yiruikeji Co., Ltd., Guangdong, China) at 8 weeks post-SCI. To analyze motor evoked potentials (MEPs) in the left hind limb of rats after SCI, we positioned stimulation electrodes on their heads. We inserted receiving electrodes into the gastrocnemius muscle of the left hind limb. Additionally, electrical electrodes were placed under the dorsal skin after the animal received anesthesia. We assessed nerve function recovery by measuring amplitude and latency.

### 5.22 Western blot

RIPA buffer (Beyotime, China), containing phosphatase and protease inhibitors, was used to extract total protein from spinal cord tissue surrounding the lesion center, as well as from BMDMs and NSCs. The protein was separated using 10 % SDS-PAGE and subsequently transferred onto a polyvinylidene fluoride (PVDF) membrane. After blocking with 5 % skim milk (BD Biosciences, USA), the PVDF membrane was incubated overnight with the primary antibody and subsequently treated with the secondary antibody. The PVDF membrane was covered with ECL reagents (Sigma-Aldrich, USA) and exposed to chemiluminescence to visualize the protein bands. ImageJ software was utilized to quantify and analyze the bands. The primary antibodies used in this research are listed below: CD68 (Abcam, Cambridge, UK), CD206 (Abcam, Cambridge, UK), GFAP (Bioss, Beijing, China), iNOS (Abcam, Cambridge, UK), Tuj-1 (Abcam, Cambridge, UK), NF-200 (Invitrogen, CA, USA), MBP (Abcam, Cambridge, UK), HIF-1α (Abcam, Cambridge, UK), VEGFA (Abcam, Cambridge, UK), P-CaMKⅡ (Abcam, Cambridge, UK), CaMKⅡ (Abcam, Cambridge, UK), P-CREB (Cell Signaling Technology, USA), CREB (Cell Signaling Technology, USA), P-PI3K (Cell Signaling Technology, USA), PI3K (Cell Signaling Technology, USA), P-AKT (Cell Signaling Technology, USA), AKT (Cell Signaling Technology, USA), Cleaved-Caspase3 (Cell Signaling Technology, USA), Bcl-2 (Cell Signaling Technology, USA), Bax (Cell Signaling Technology, USA), GAPDH (Proteintech, IL, USA). The secondary antibodies used were HRP-conjugated Goat Anti-Mouse IgG (H + L) (Beyotime, China) and HRP-conjugated Goat Anti-Rabbit IgG (H + L) (Beyotime, China).

### 5.23 Real-time polymerase chain reaction (RT-qPCR)

Total RNA was extracted from the tissues using an extraction buffer (Trizol/phenol/chloroform). The purified RNA was reverse-transcribed into complementary DNA (cDNA) using the HiFiScript gDNA Removal RT MasterMix (CWBio, China). Subsequently, RT-qPCR assays were performed with UltraSYBR Mixture (CWBio, China). All PCR reactions were carried out on a Roche LightCycler®96 real-time PCR system, and all procedures were strictly performed in accordance with the manufacturer's instructions. The sequences of primers used in this experiment are listed in Table 1.

**Table 1:** Sequences of primers for RT-PCR.

Gene	Forward primer sequence (5′–3′)	Reverse primer sequence (5′–3′)
IL-1β	GCT TCC TTG TGC AAG TGT CT	TCT GGA CAG CCC AAG TCA AG
TNF-α	ATG GGC TCC CTC TCA TCA GT	GCT TGG TGG TTT GCT ACG AC
GAPDH	AGT GCC AGC CTC GTC TCA TA	AGC CCT GTA TTC CGT CTC CT

### 5.24 Cryopreservation of NSCs and PM@NSCs

The prepared NSCs and PM@NSCs were resuspended in cryoprotective solution. The cryoprotective solution was prepared in advance with the specific formula: 10 % dimethyl sulfoxide (DMSO, Sigma-Aldrich, USA), 20 % fetal bovine serum (FBS, Gibco, USA), and 70 % complete medium (DMEM/F12 + Glucose + penicillin/streptomycin). Cryovials were prepared and pre-labeled with the cell name, passage number, cell count, the initials of the operator, and the cryopreservation date. The cell pellet was resuspended in the prepared cryoprotective solution, and 1 mL of the cell suspension was aliquoted into each cryovial. The aliquoted cryovials were placed in a cryopreservation container filled with isopropanol, stored in a −80 °C refrigerator overnight (1 day), and then transferred to a liquid nitrogen tank for long-term storage.

### 5.25 Statistical analysis

Statistical analyses were performed using GraphPad Prism 9.4.1 software (GraphPad Software, Inc., La Jolla, CA, USA) and G∗Power 3.1 software (Heinrich-Heine-Universität Düsseldorf, Germany). All quantitative data are presented as mean ± standard error of mean (SEM). Prior to formal statistical analysis, all datasets were first evaluated for normality via the Shapiro-Wilk test and for homogeneity of variance using the Brown-Forsythe test. For data that met the assumptions of normality and homogeneous variance, parametric tests were applied: one-way or two-way analysis of variance (ANOVA) was used for multiple group comparisons, followed by Tukey's post hoc test for pairwise comparisons. For data that failed to meet the parametric test assumptions, nonparametric tests were employed. The Kruskal-Wallis test was used for multiple group comparisons, with Dunn's post hoc test used for subsequent pairwise analyses. The sample size was determined via a priori power analysis using G∗Power 3.1 software, and all data in this study met the statistical power requirements. Statistical significance was defined as follows: ∗p < 0.05, ∗∗p < 0.01, ∗∗∗p < 0.001, ∗∗∗∗p < 0.0001.

## CRediT authorship contribution statement

**Jie Ren:** Writing – review & editing, Writing – original draft, Visualization, Validation, Software, Resources, Methodology, Investigation, Formal analysis, Data curation, Conceptualization. **Junjin Li:** Writing – review & editing, Writing – original draft, Visualization, Validation, Software, Methodology. **Hongda Wang:** Visualization, Validation, Software, Methodology, Data curation. **Haiwen Feng:** Software, Methodology. **Huaying Hao:** Writing – original draft, Visualization, Software, Methodology. **Junyu Chen:** Methodology, Investigation, Data curation. **Yuanquan Li:** Methodology. **Zhengyu Xu:** Methodology. **Chuanhao Li:** Methodology. **Wang Jiang:** Resources. **Yan Wang:** Methodology. **Xiaoyang Zhang:** Methodology. **Xiaomeng Song:** Writing – original draft, Validation, Supervision. **Guangzhi Ning:** Writing – original draft, Validation, Supervision. **Jun Liang:** Writing – original draft, Project administration, Funding acquisition. **Shiqing Feng:** Writing – original draft, Resources, Project administration, Investigation, Funding acquisition.

## Ethics approval and consent to participate

A thorough evaluation was conducted for all procedures involving animal experimentation, which received official endorsement from the Animal Care and Use Committee of Tianjin Medical University General Hospital (IRB2024-DW-44). This ensured complete adherence to ethical regulations.

## Data availability statement

The data that support the findings of this study are available on request from the corresponding author. The data are not publicly available due to privacy or ethical restrictions.

## Declaration of competing interest

The authors declare no conflict of interest.
