# Supplementary material for: Integrated cryopreservation-thawing-transplantation platform for neural stem cell-based spinal cord injury repair
Source: Bioact Mater. 2026 Jan 30;60:401–24. doi: 10.1016/j.bioactmat.2026.01.024 (PMC12874286; doi:10.1016/j.bioactmat.2026.01.024)
Supplement: Multimedia component 1 [file mmc1.docx]

**Integrated Cryopreservation-Thawing-Transplantation Platform for Neural Stem Cell-Based Spinal Cord Injury Repair**


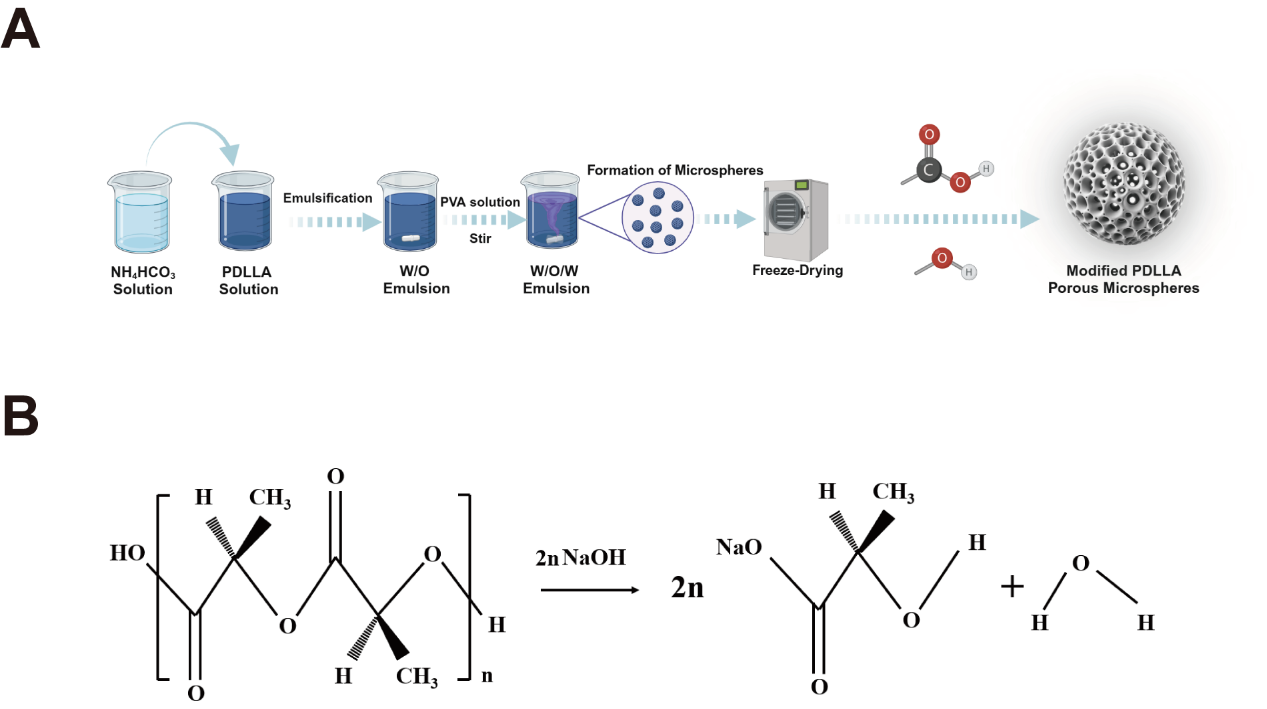


**Figure S1. Preparation and modification of PDLLA porous microspheres.**

A) Schematic diagram of the preparation process of PDLLA porous microspheres.

B) Hydrolysis mechanism of PDLLA porous microspheres cleavage of ester bonds and introduction of hydroxyl and carboxyl groups.

**
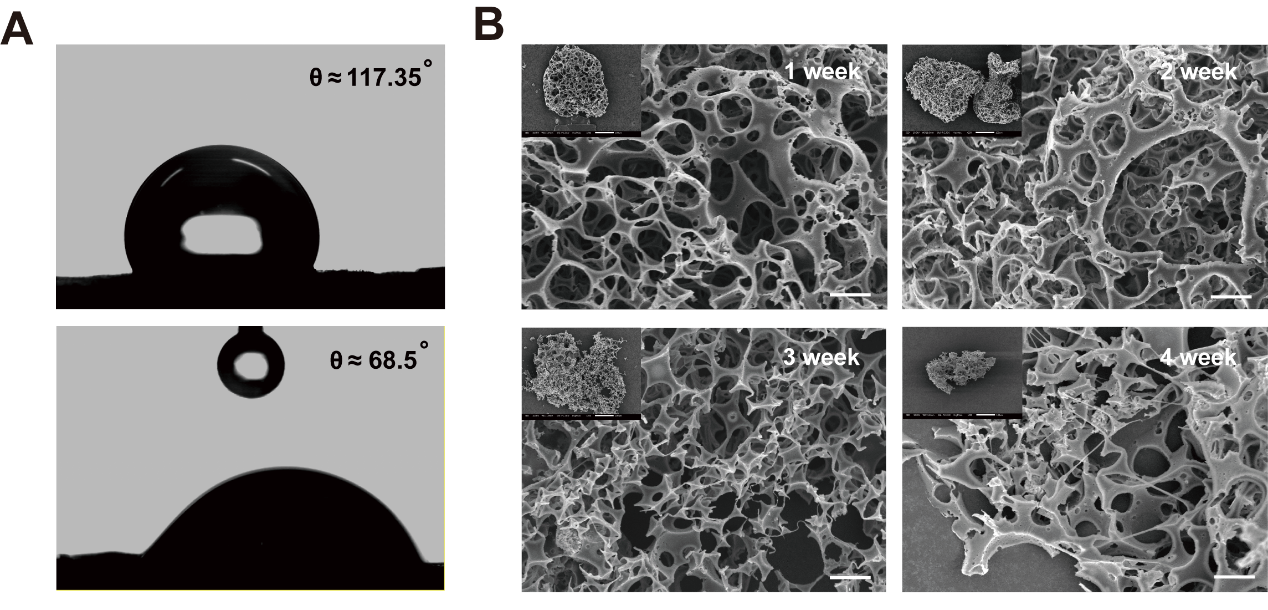
**

**Figure S2. Characterization of PDLLA porous microspheres (PM).**

A) Contact angle measurements of PDLLA porous microspheres before and after hydrolysis.

B) Degradation of PDLLA porous microspheres in PBS (pH 7.4) at 37°C over four weeks, as observed by SEM. Scale bar: 20 μm.

**
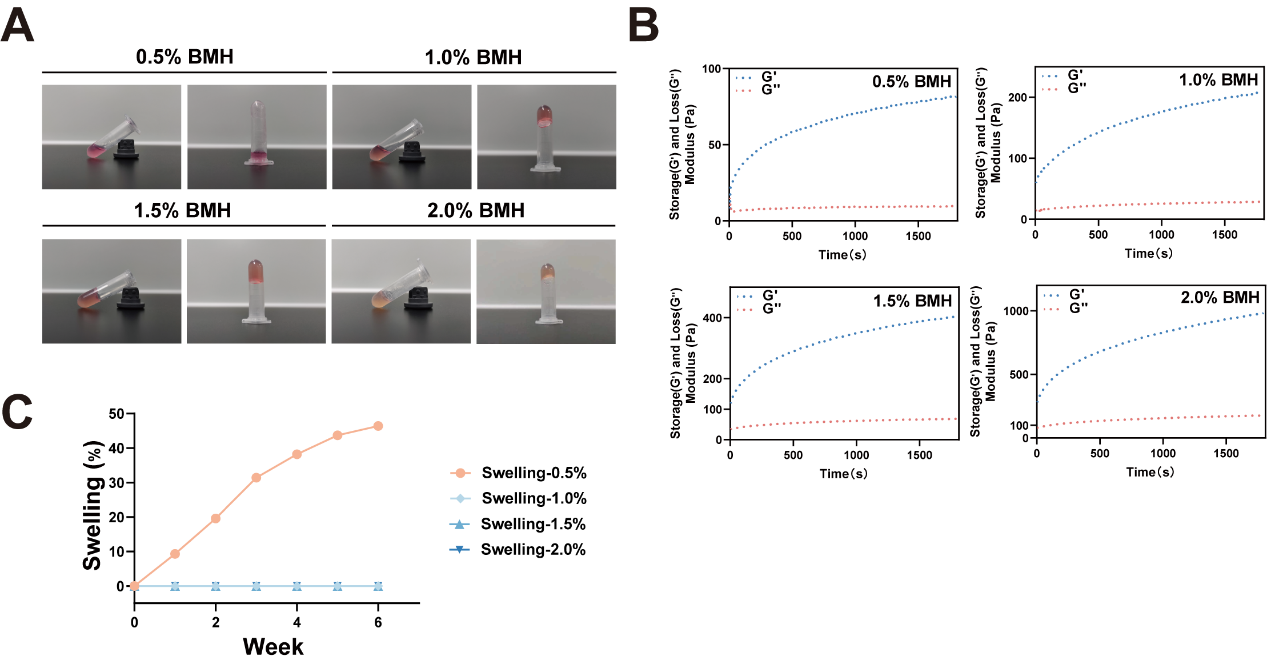
**

**Figure S3. Characterization of biomimetic matrix hydrogel (BMH).**

A) Macroscopic appearance of hydrogels formed at different concentrations.

B) Rheological analysis of biomimetic matrix hydrogel at different concentrations (0.5 wt.%, 1.0 wt.%, 1.5 wt.%, and 2.0 wt.%).

C) Swelling properties of the hydrogel at various concentrations over time in PBS (pH 7.4).

**
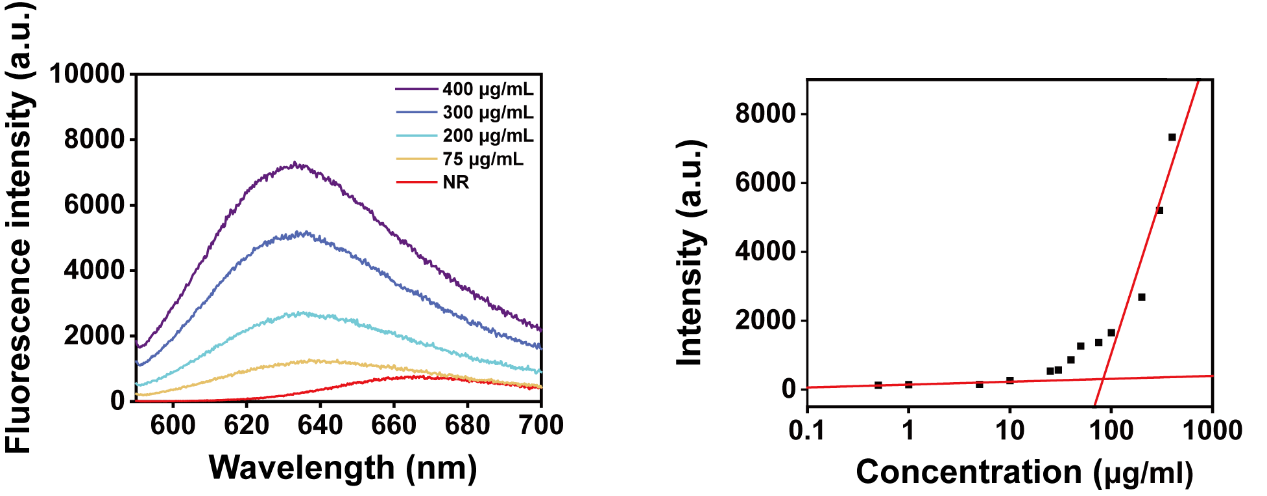
**

**Figure S4. The critical aggregation concentration of the biomimetic matrix hydrogel. The left panel shows the Nile Red fluorescence spectra of the biomimetic matrix hydrogel at various concentrations. The right panel displays the Logarithmic plot of hydrogel concentration versus fluorescence intensity.**

**
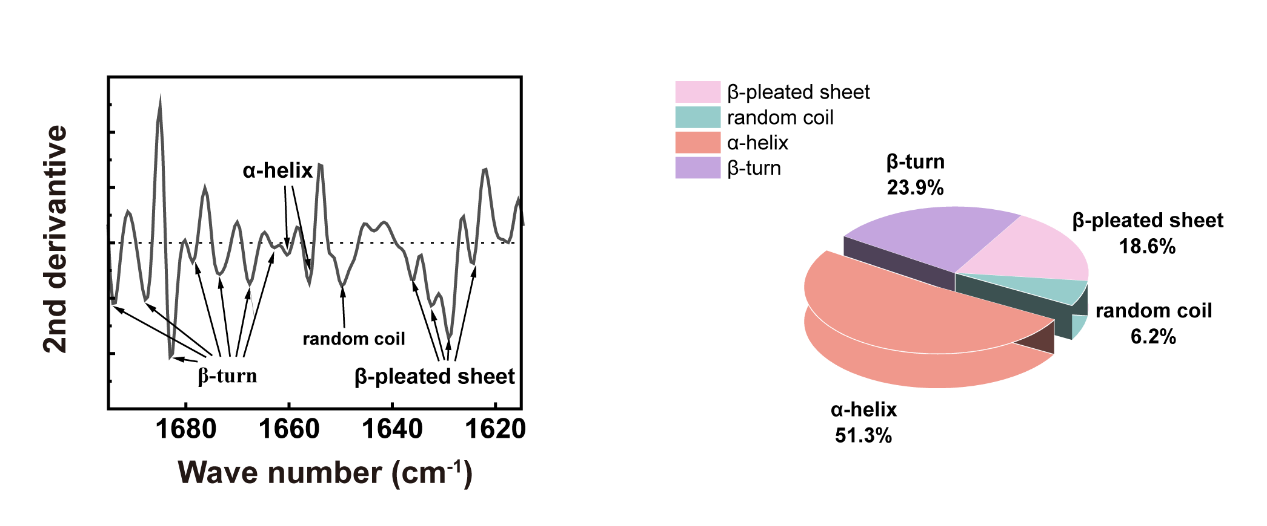
**

**Figure S5. The second derivative plot of the FTIR for the biomimetic matrix hydrogels and the corresponding percentage of secondary structures in the assembled system.**

**
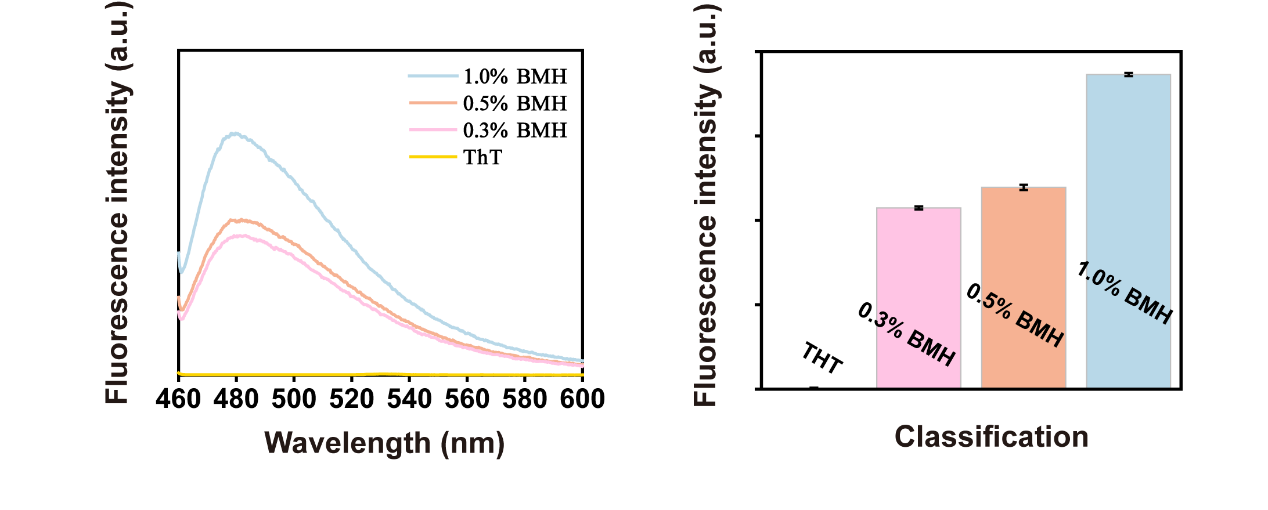
**

**Figure S6. ThT binding fluorescence spectra of the hydrogel at concentrations of 0.3%, 0.5%, and 1.0%.**

**
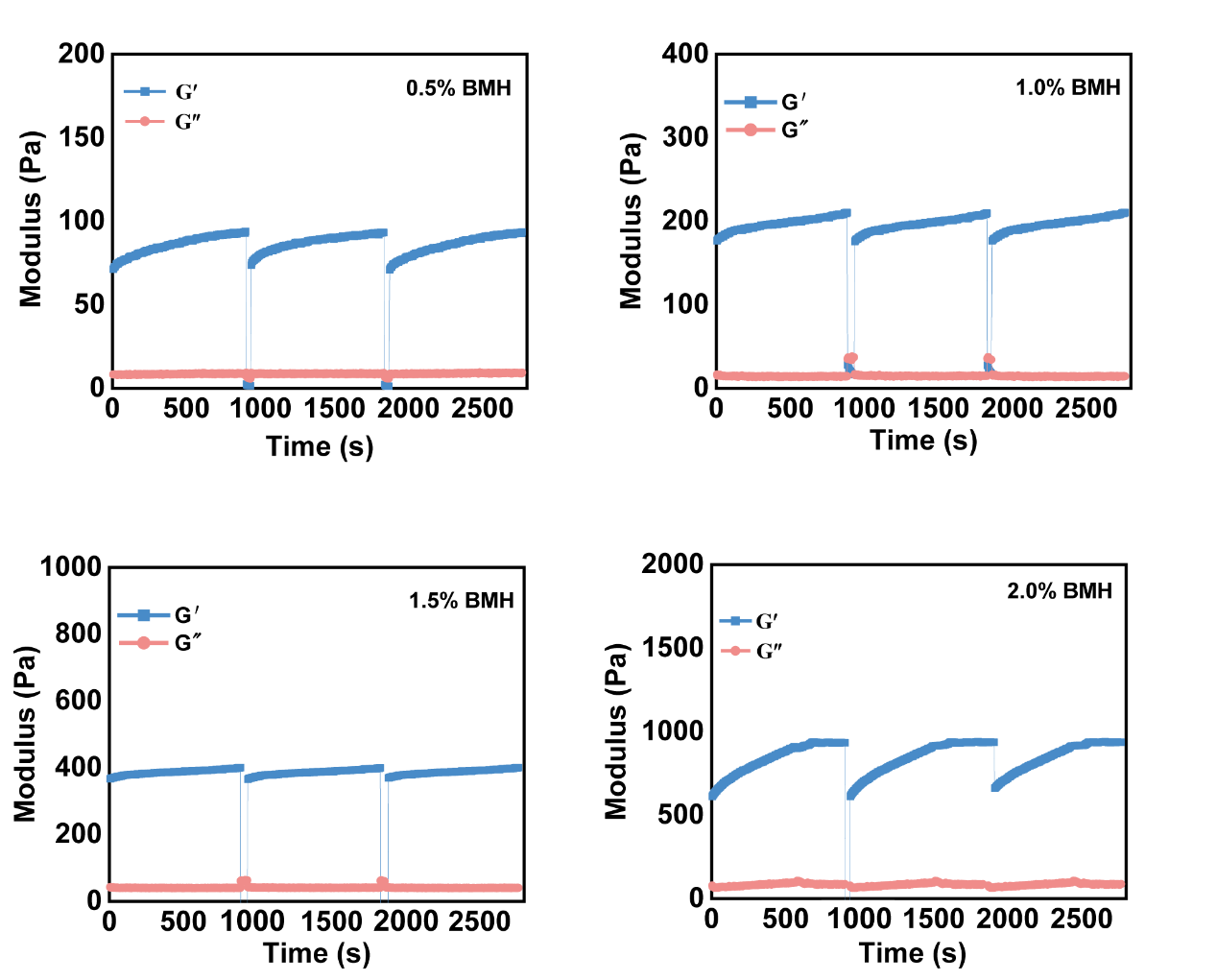
**

**Figure S7. Assessment of recovery characteristics of 0.5%, 1.0%, 1.5%, and 2.0% biomimetic matrix hydrogels after shear thinning.**

**
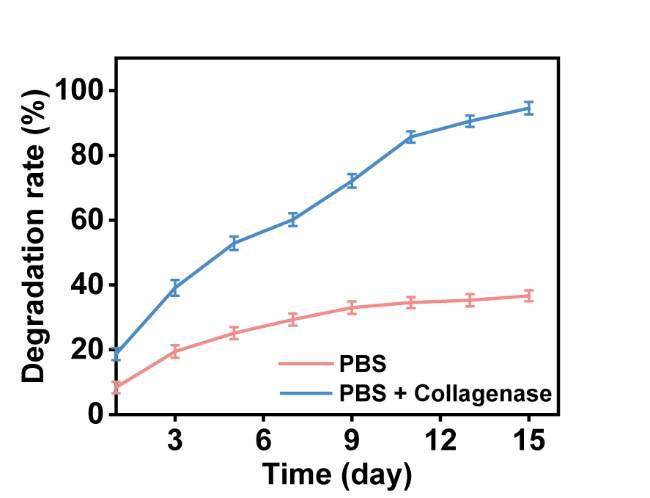
**

**Figure S8. In vitro degradation of the biomimetic matrix hydrogel.**

**
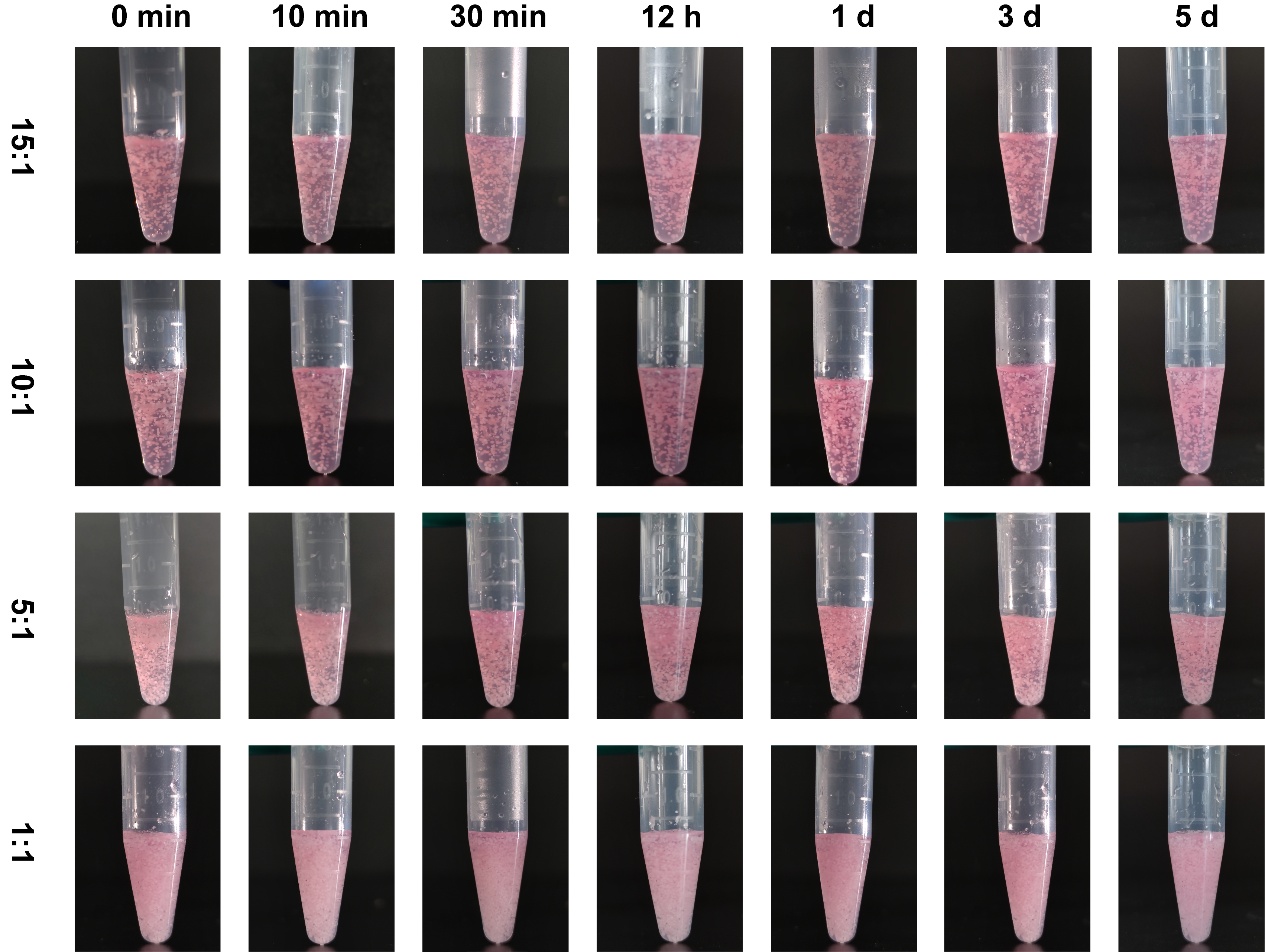
**

**Figure S9. Stability of microsphere suspensions at different mass ratios over time.** Representative images showing the sedimentation behavior of microsphere suspensions at mass ratios of 15:1, 10:1, 5:1, and 1:1 (microspheres to hydrogel) at time points of 0 min, 10 min, 30 min, 12 h, 1 d, 3 d, and 5 d.

**
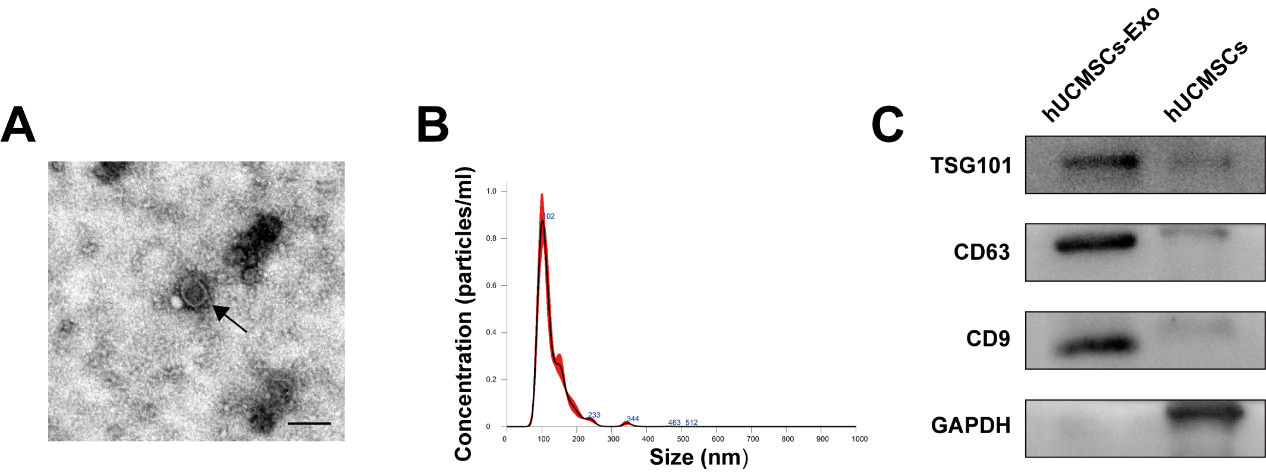
**

**Figure S10. Characterization of** **hUCMSC-Exo.**

A) TEM image of hUCMSC-Exo, showing characteristic cup-shaped morphology. Scale bar: 100 nm.

B) Size distribution of hUCMSC-Exo decided by nanoparticle tracking analysis (NTA).

C) Western blot analysis of exosome-specific markers (TSG101, CD9 and CD63) in hUCMSC-Exo, with GAPDH as a control.

**
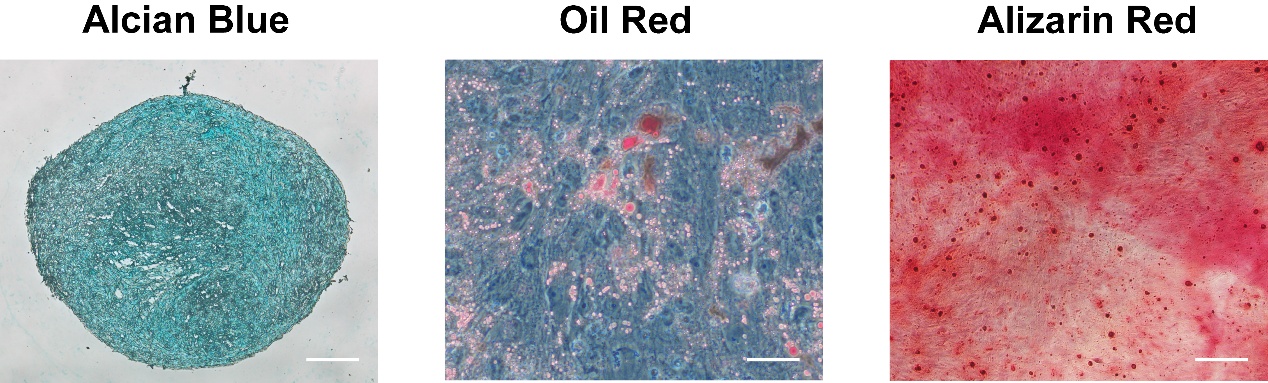
**

**Figure S11. Trilineage differentiation potential of hUCMSCs.**

Representative images of Alcian Blue staining for chondrogenic differentiation (left), Oil Red O staining for adipogenic differentiation (middle), and Alizarin Red staining for osteogenic differentiation (right), showing the trilineage differentiation potential of hUCMSCs. Scale bars: 50 μm.

**
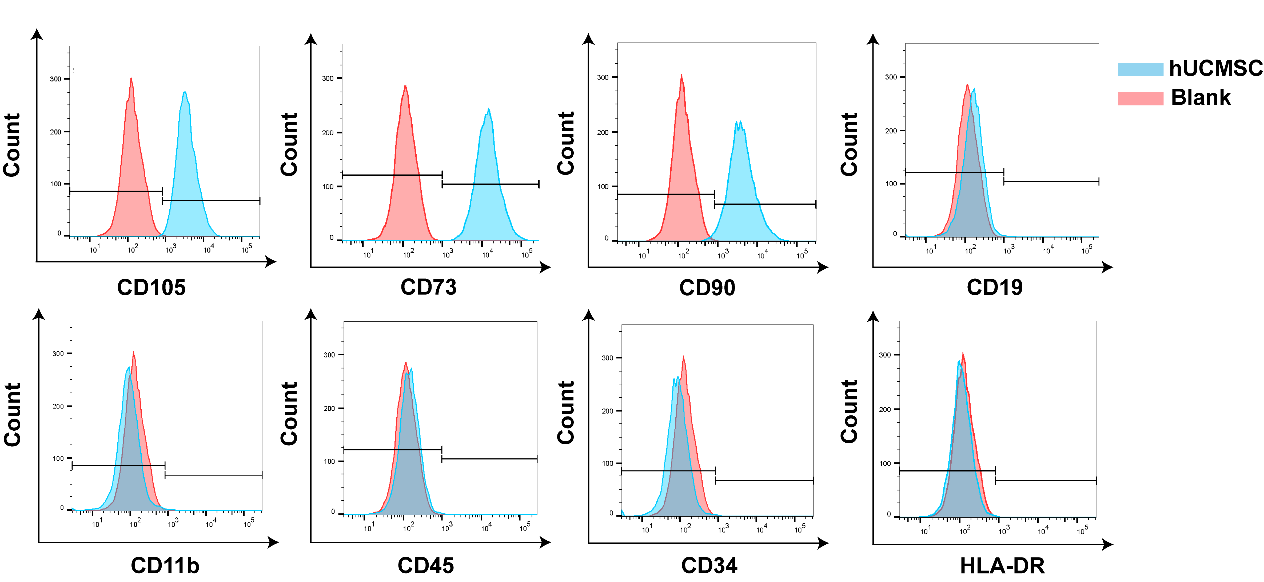
**

**Figure S12. Flow cytometric analysis of surface markers in hUCMSCs.**

Flow cytometry histograms showing positive expression of hUCMSC markers (CD105, CD73, and CD90) and negative expression of hematopoietic or immune markers (CD19, CD11b, CD45, CD34, and HLA-DR) in hUCMSCs (blue) compared to blank controls (red).

**
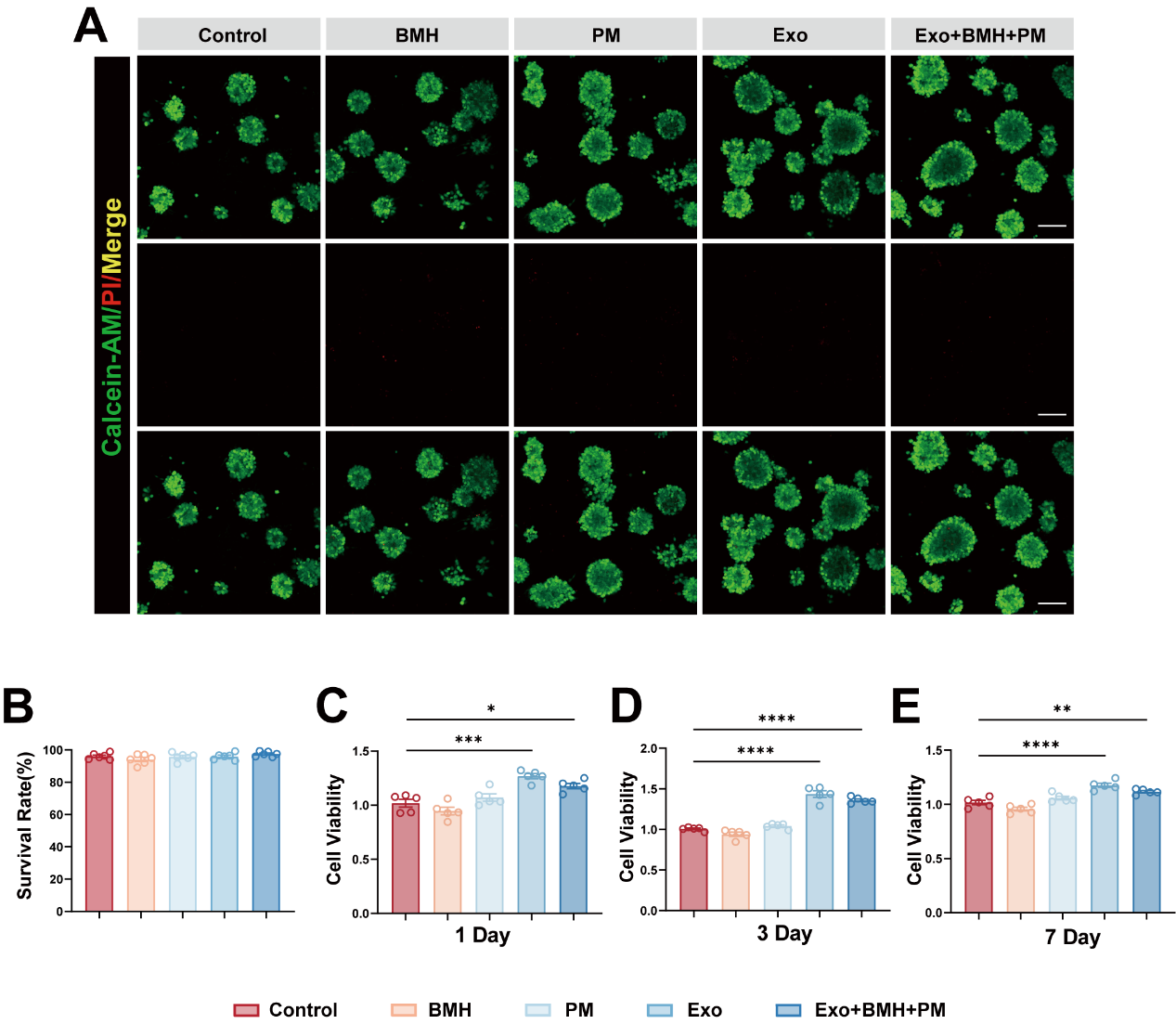
**

**Figure S13. In vitro biocompatibility of the composite delivery system.**

A) Live/Dead staining of neural stem cells (NSCs) co-cultured with different groups for three days. Green fluorescence shows live cells (Calcein-AM), and red fluorescence shows dead cells (PI). Scale bar: 100 μm.

B) Quantitative analysis of survival rates showing high cell viability in all groups (n = 6).

C-E) CCK-8 assay results for NSC viability at 1, 3, and 7 days (n = 5), with Exo and BMH+PM+Exo groups showing significant increases.

All data are presented as the mean ± SEM. *p < 0.05, **p < 0.01, ***p < 0.001, ****p < 0.0001.

**
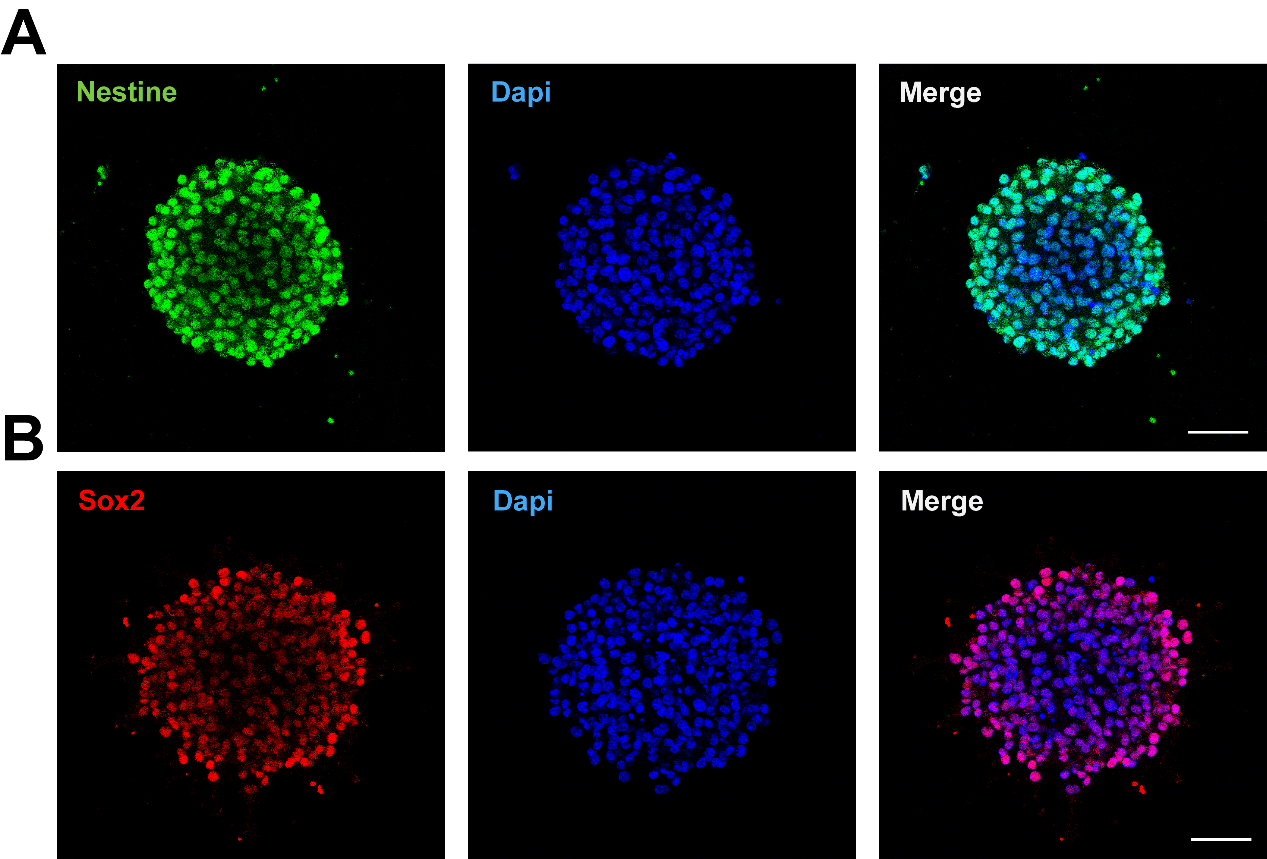
**

**Figure S14. Characterization of neural stem cell spheroids.**

A) Representative confocal images of Nestin (green) and DAPI (blue) in NSC spheroids. Scale bar: 50 μm.

B) Representative confocal images of Sox2 (red) and DAPI (blue) in NSC spheroids. Scale bar: 50 μm.

**
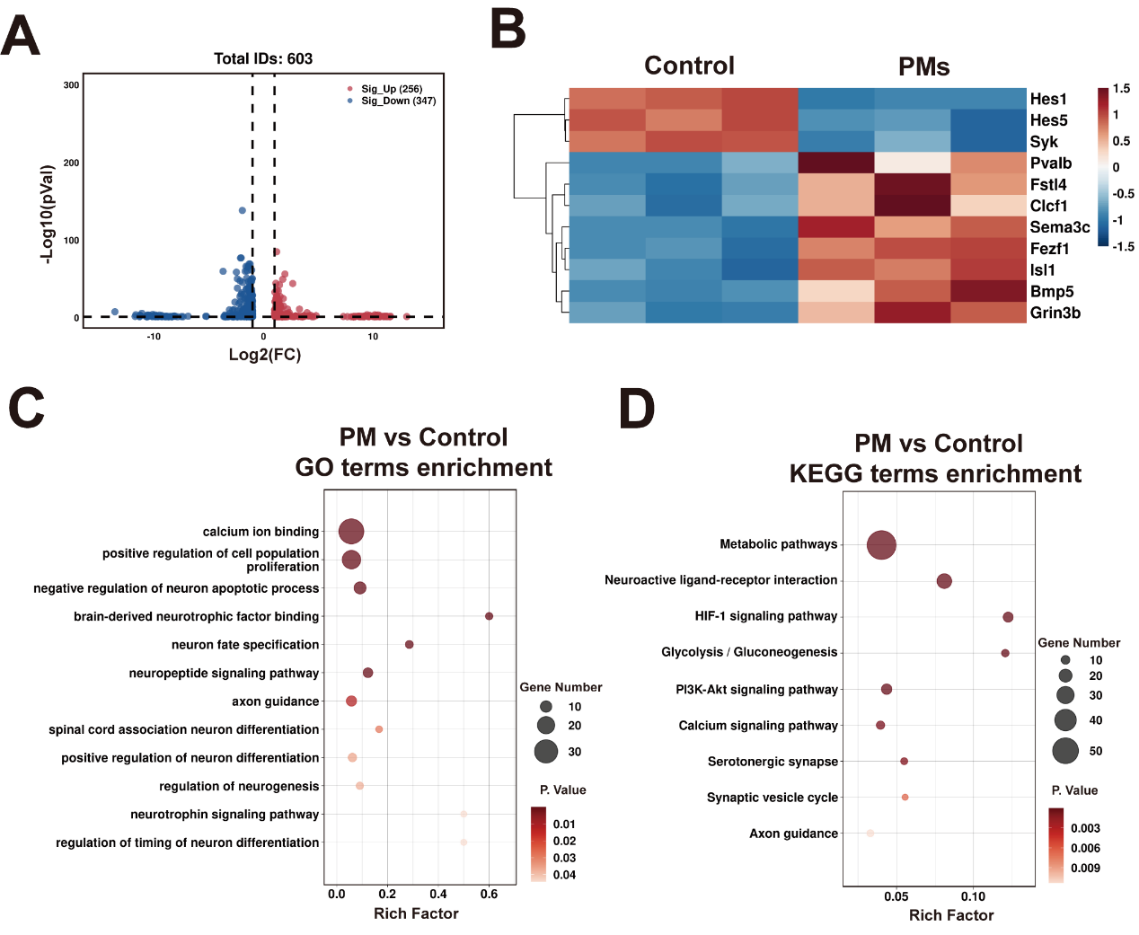
**

**Figure S15. RNA sequencing analysis of gene expression in neural stem cells (NSCs) co-cultured with PM.**

A) Volcano plots of differentially expressed genes (DEGs) in the PM vs. control. DEGs are defined as |log2FC| ≥ 1 with q < 0.05.

B) Heatmaps of DEGs for PM vs. Control.

C, D) GO (C) and KEGG (D) pathway enrichment analysis of differentially expressed genes in NSCs after intervention with + PM.

**
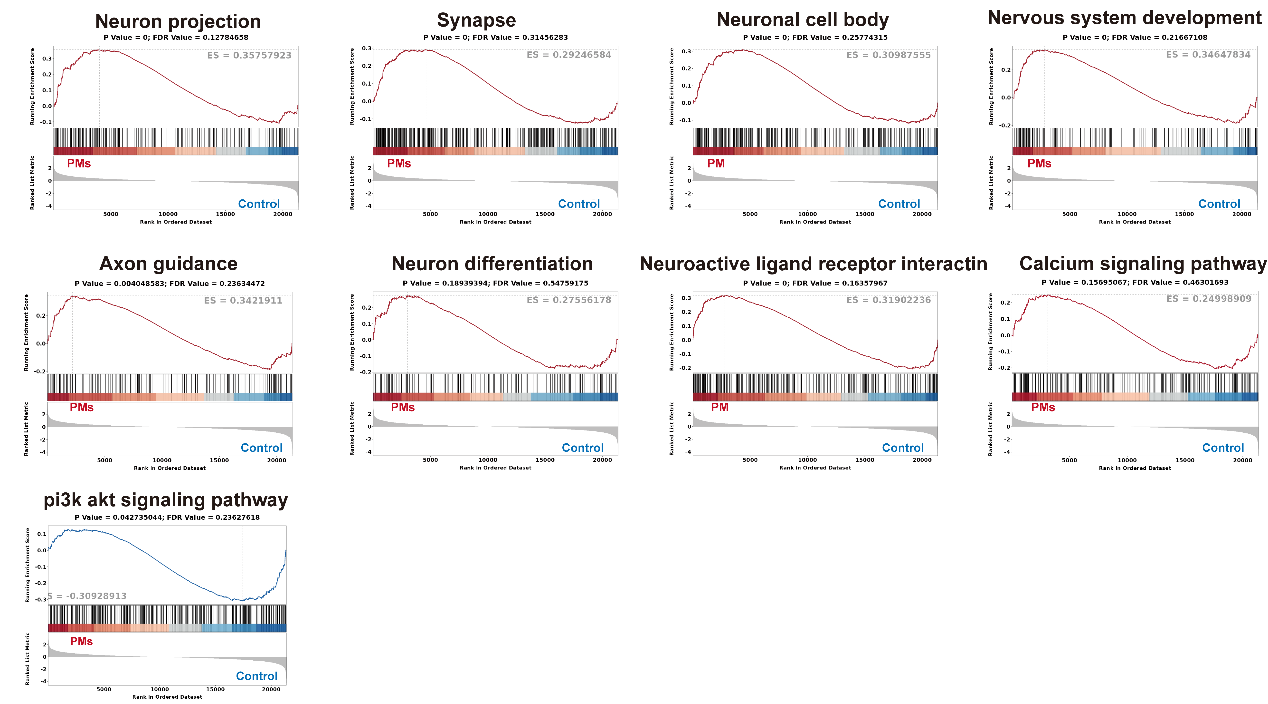
**

**Figure S16. Gene Set Enrichment Analysis (GSEA) for NSCs treated with PM.** GSEA showing pathways significantly positively correlated with differentially expressed genes in the PM group. Enrichment scores (ES), p-values, and false discovery rates (FDR) are shown for each pathway.

**
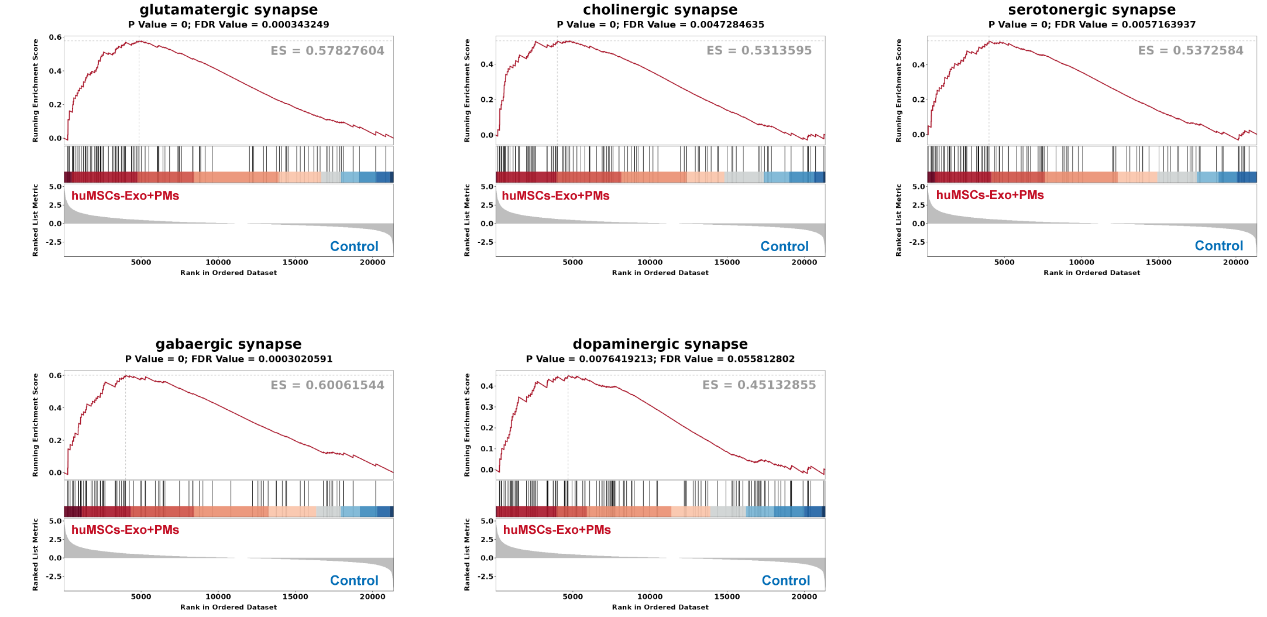
**

**Figure S17. Gene Set Enrichment Analysis (GSEA) of synapse-related in transcriptomic data.** Enrichment plots for glutamatergic, cholinergic, GABAergic, dopaminergic, and serotonergic synapse pathways, showing significant upregulation in huMSCs-Exo+PM-treated groups compared to controls. Enrichment scores (ES), p-values, and false discovery rates (FDR) are shown for each pathway.

**
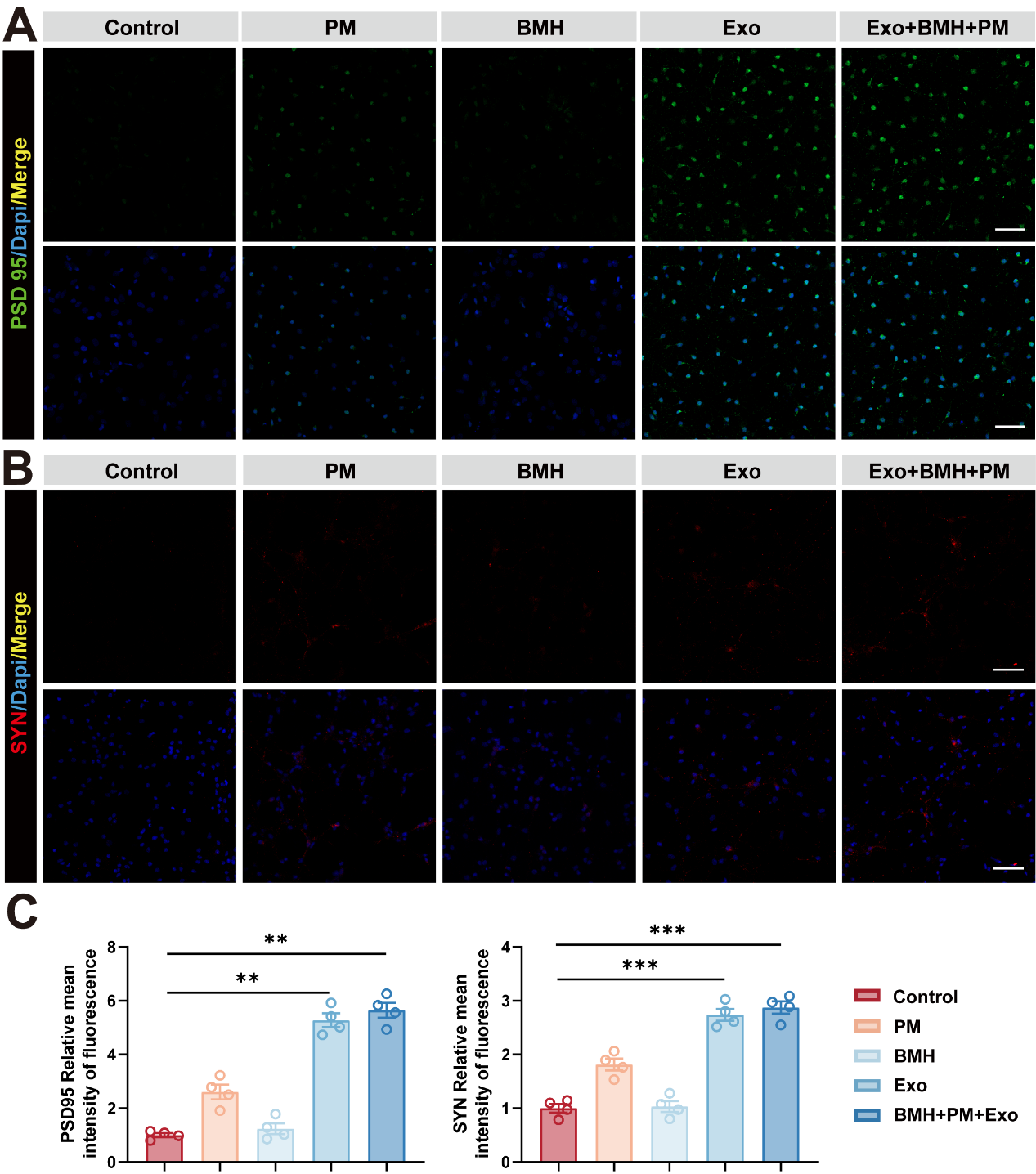
**

**Figure S18. Composites (BMH@Exo-PM) Promote the Synaptic-Related Protein Expression in NSCs.**

A-B) Representative confocal images of NSCs in different treatment groups for 7 days. NSCs were stained with PSD95 (green), SYN (red), and DAPI (blue). Scale bar: 50 μm.

C) Quantitative analysis of PSD95 and SYN relative mean intensity of fluorescence area in each group (n = 4).

All data are presented as the mean ± SEM. **p < 0.01, ***p < 0.001.

**
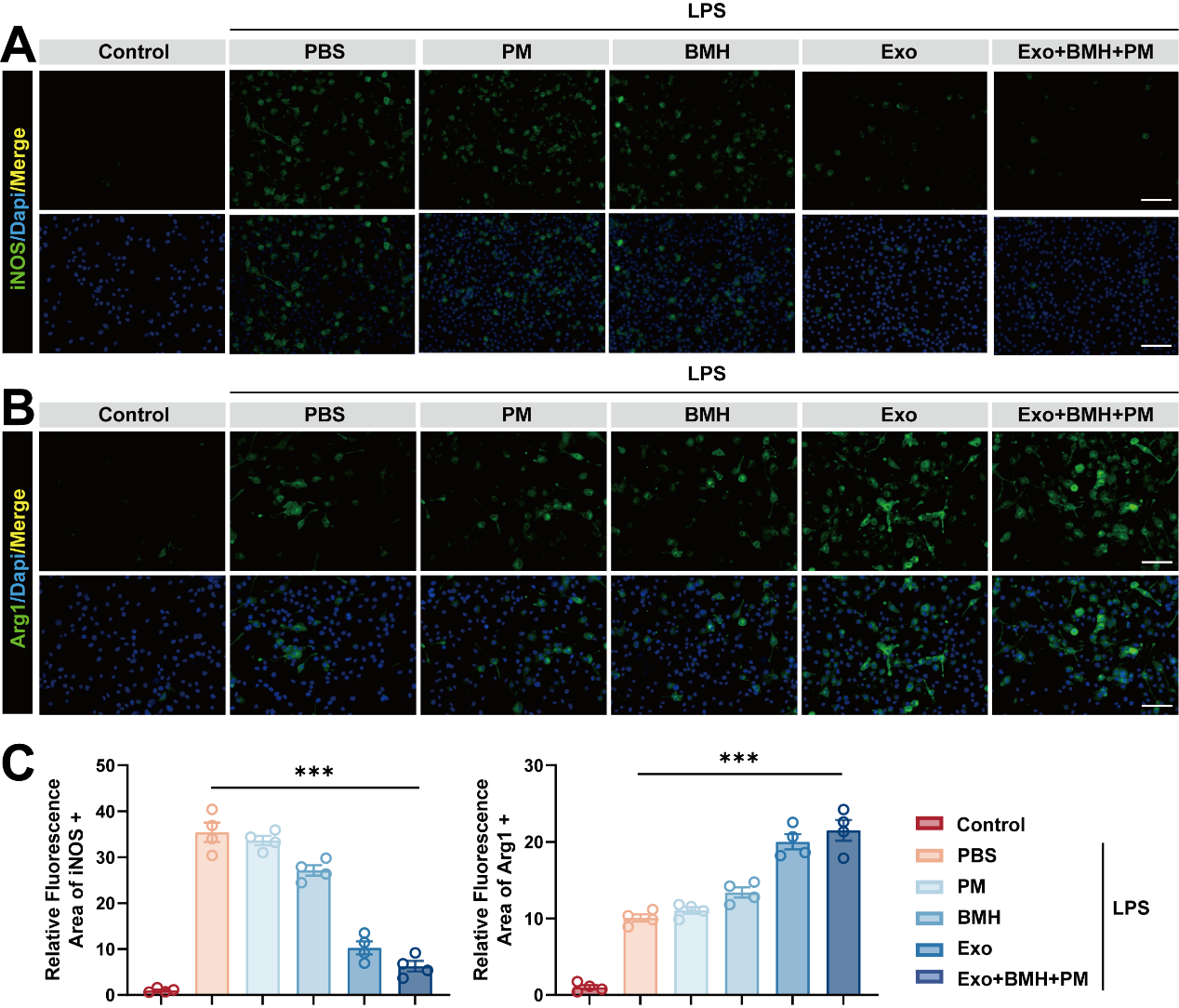
**

**Figure S19. Composites (BMH@Exo-PM) Modulate the Polarization of Microglial Cell Line BV2 in Vitro to Suppress Inflammatory Responses**

A) Representative immunofluorescence images of BV2s (LPS for 24h) treated with different groups. BV2s were stained with iNOS (green). Scale bar: 100 μm.

B) Representative immunofluorescence images of BV2s (LPS for 24h) treated with different groups. BV2s were stained with Arg1 (green). Scale bar: 100 μm.

C) Quantitative analysis of iNOS and Arg1 relative fluorescence area in each group (n = 4).

All data are presented as the mean ± SEM. ***p < 0.001.

**
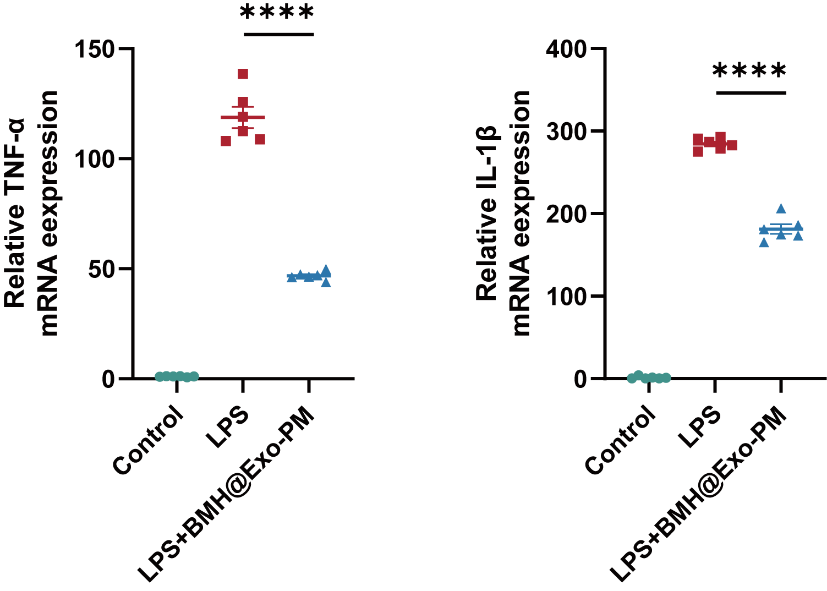
**

**Figure S20. Quantitative RT-qPCR analysis of BMDMs showing mRNA expression of pro-inflammatory cytokines IL-1β and TNF-α following 12-hour treatment.**

GAPDH was employed as the internal reference gene (loading control) for data normalization. Data are presented as fold changes relative to the untreated control group (n = 6).

**
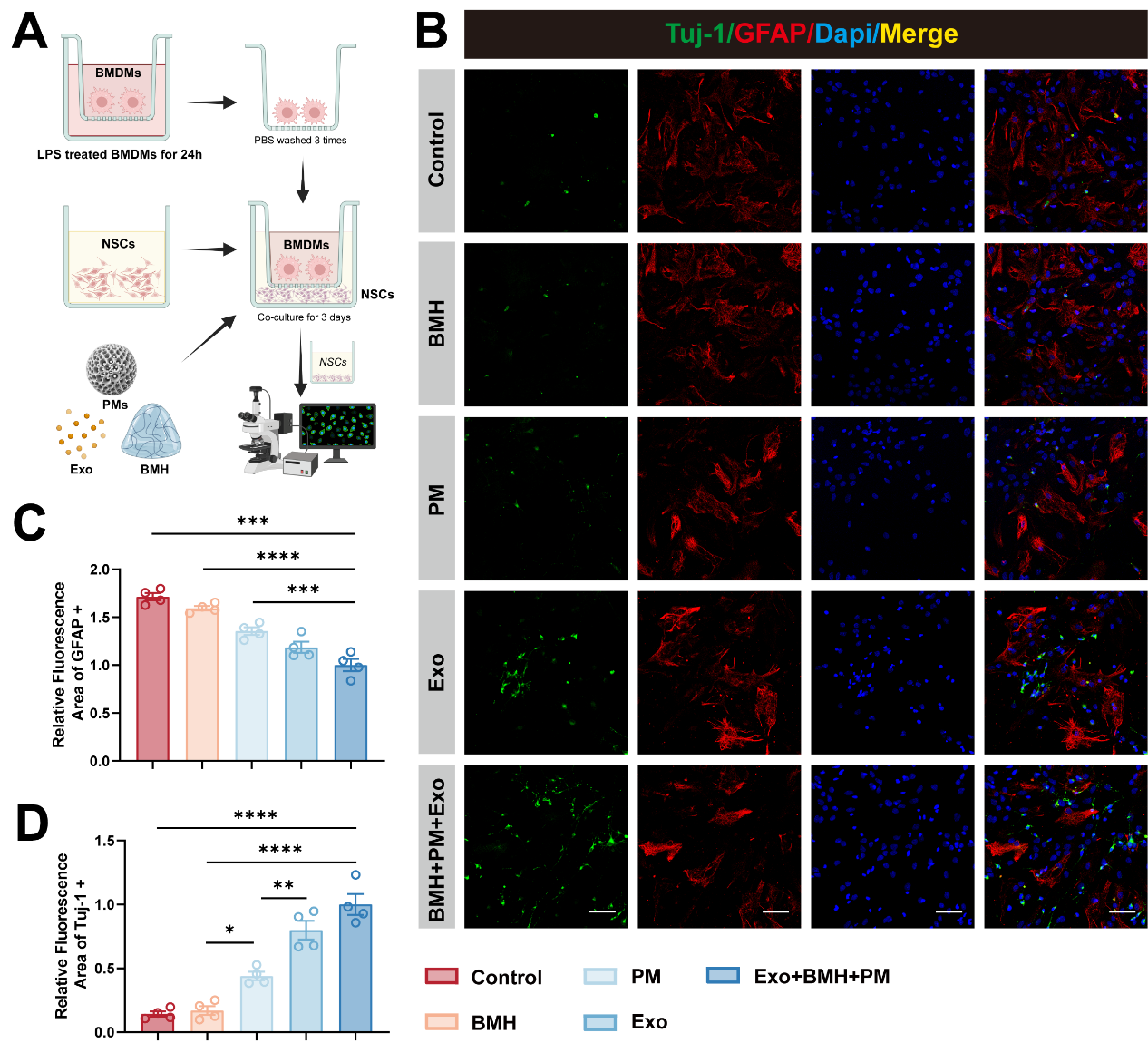
**

**Figure S21. Composite (BMH@Exo-PM) Promotes Neural Stem Cell (NSC) Differentiation under Simulated Inflammatory Microenvironment In Vitro.**

A) Schematic of the co-culture system of NSCs with LPS-treated BMDMs and composite intervention.

B) Representative confocal images of NSCs in different treatment groups for 7 days. NSCs were stained with Tuj-1 (green), GFAP (red), and DAPI (blue). Scale bar: 50 μm.

C-D) Quantitative analysis of GFAP (C) and Tuj-1 (D) relative fluorescence area in each group (n = 4).

All data are presented as the mean ± SEM. *p < 0.05, **p < 0.01, ***p < 0.001, ****p < 0.0001.

**
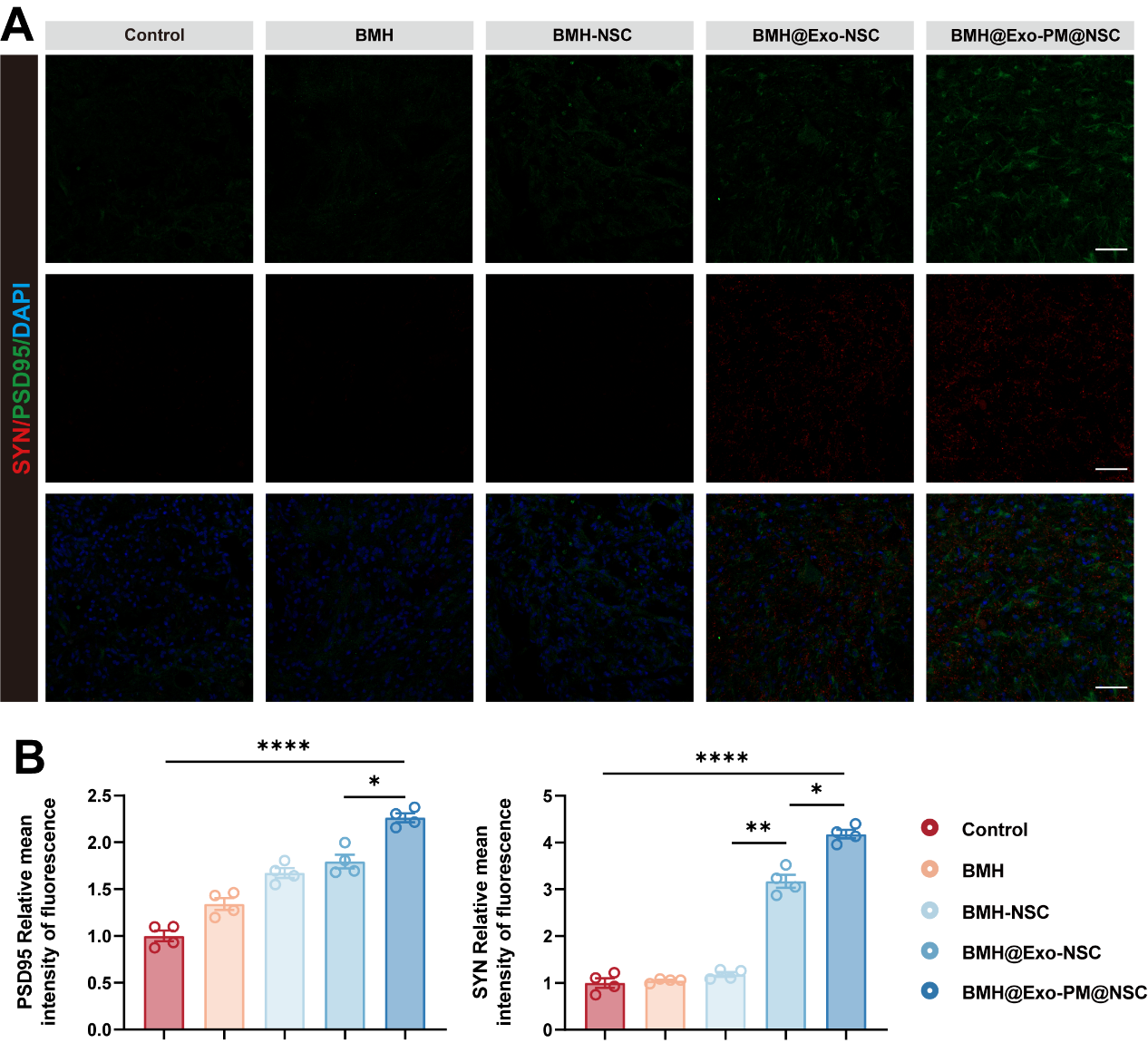
**

**Figure S22. Upregulation of Synaptic Function-Associated Proteins via Implantation of the “CTT” System (BMH@Exo-PM@NSC) at 56 Days Post-Spinal Cord Injury.**

A) Representative confocal images of PSD95 (green) and SYN (red) in each group at the injured site 56 days after SCI. Scale bar = 50 μm.

B) Quantitative analysis of PSD95 (green) and SYN (red) mean fluorescence intensities in each group (n = 4).

**
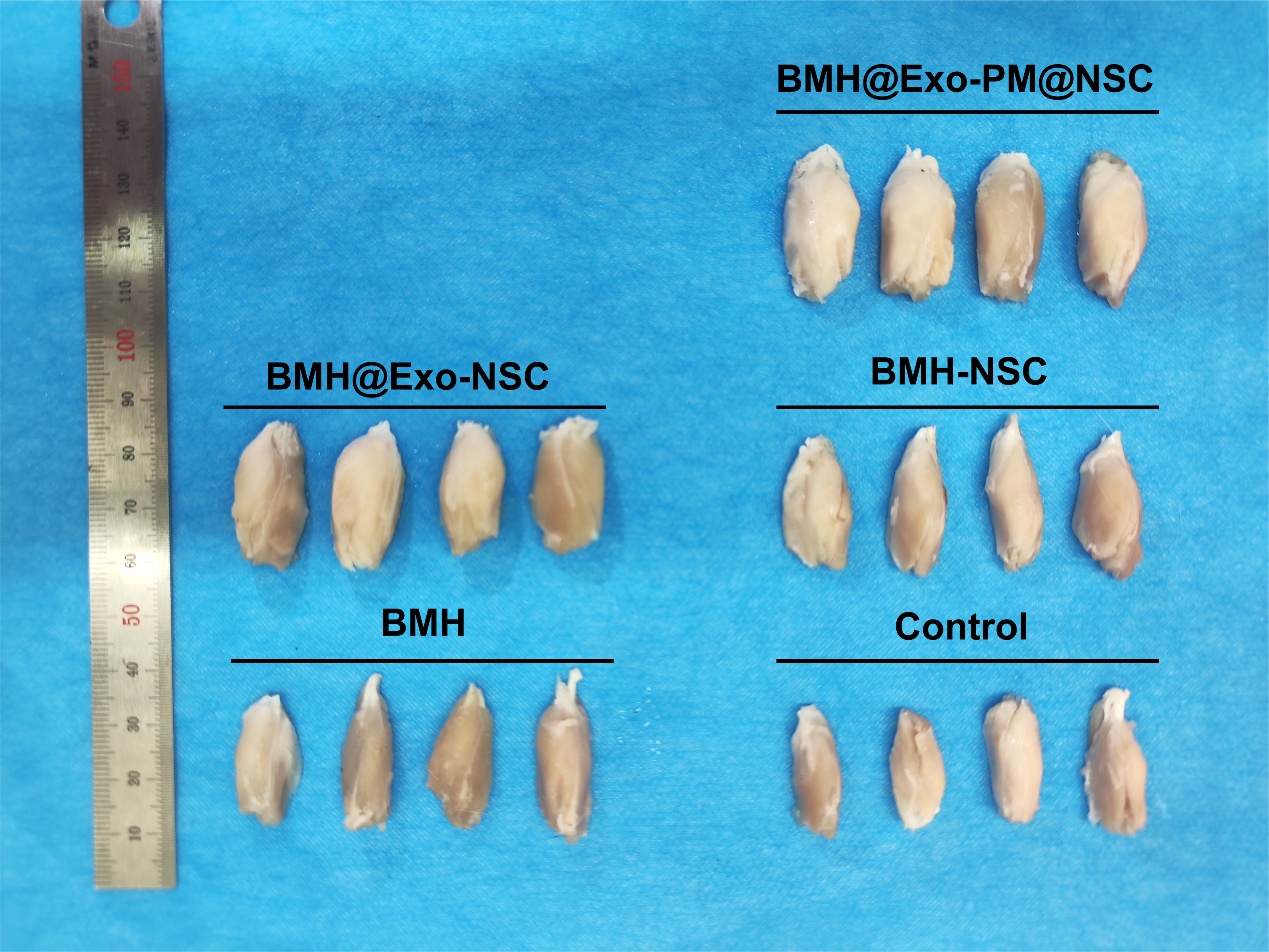
**

**Figure S23. Representative images of gastrocnemius muscles from different experimental groups.**

Representative images of hind limb muscles collected from different treatment groups, including Control, BMH, BMH-NSC, BMH@Exo-NSC, and BMH@Exo-PM@NSC, showing differences in muscle size and appearance (n = 4). A ruler is included for scale.


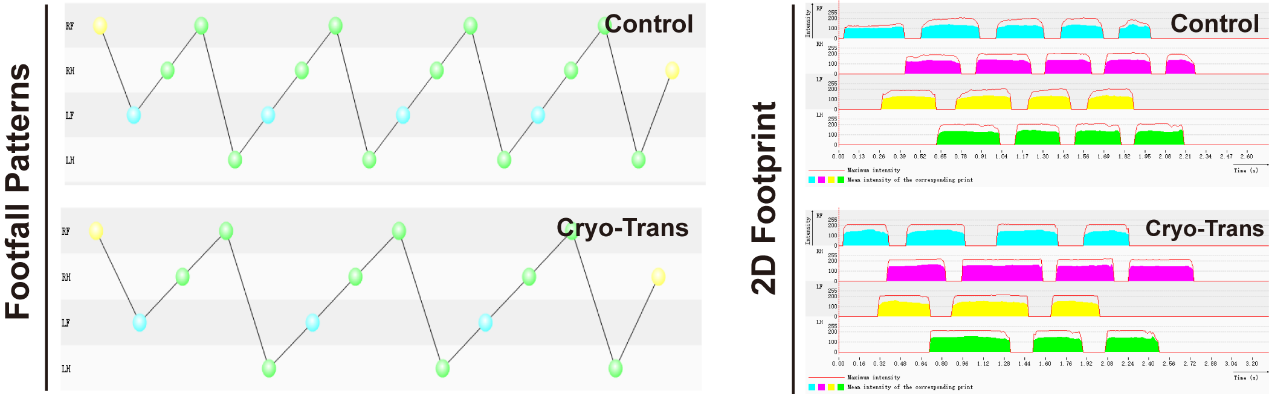


**Figure S24. Footprint analysis with footfall patterns and 2D footprints revealing differences in gait patterns among different groups.**

Left: Footfall Patterns shows no obvious differences in footfall timing trajectories between Control and Cryo-Trans groups. Right: 2D Footprint presents no significant differences in footprint morphology and distribution between the two groups (n = 5).

**
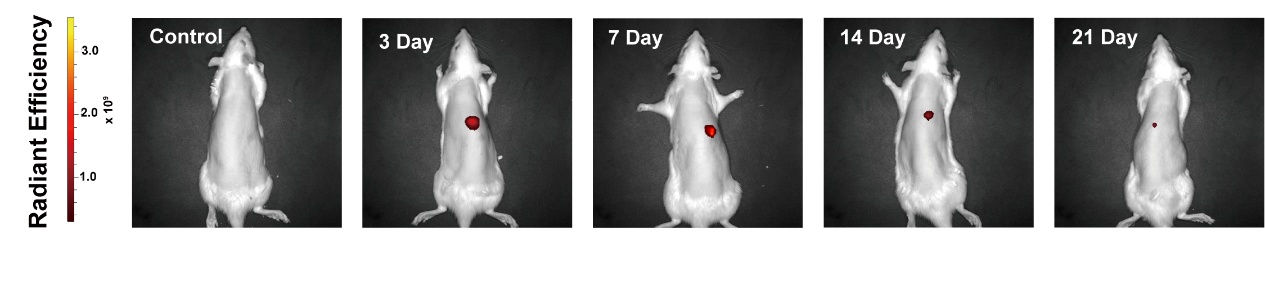
**

**Figure S25. In vivo degradability of the composite delivery system.**

In vivo degradation of Cy7-labeled porous microspheres implanted subcutaneously in rats. Fluorescence imaging at days 3, 7, 14, and 21 post-implantation reveals gradual degradation, with almost complete degradation by day 21.

**
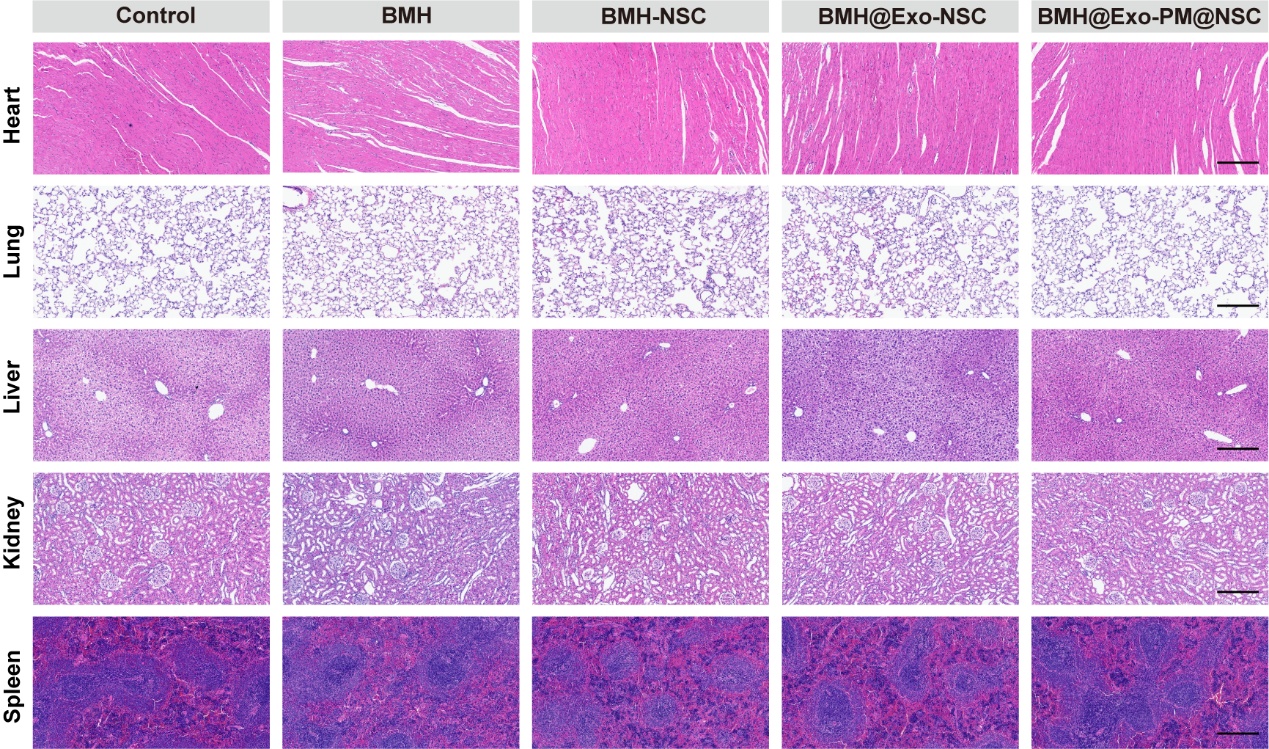
**

**Figure S26. In vivo biocompatibility of the composite delivery system.**

H&E staining images of the heart, lung, kidney, liver, and spleen after implantation in rat for eight weeks. Scale bar: 300 μm.

**
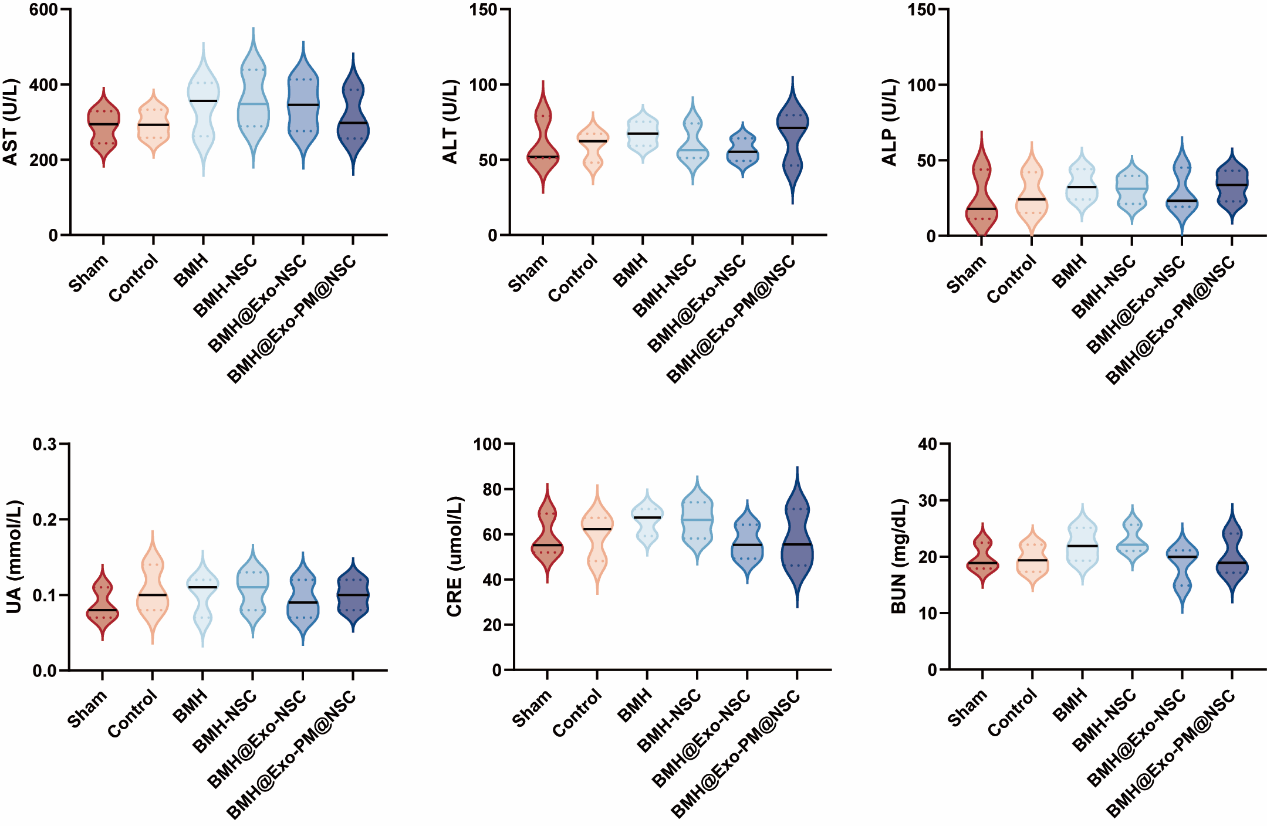
**

**Figure S27. Systemic toxicity evaluation.**

In vivo animal experiments demonstrated that the levels of key liver and kidney function markers including ALT, AST, ALP, CREA, BUN, and UA in SCI rats receiving different therapeutic regimens were within the normal range at 2 months after SCI, indicating that the CTT system (BMH@Exo-PM@NSC) proposed in this study did not cause systemic toxicity in SCI rats (n=3).

**
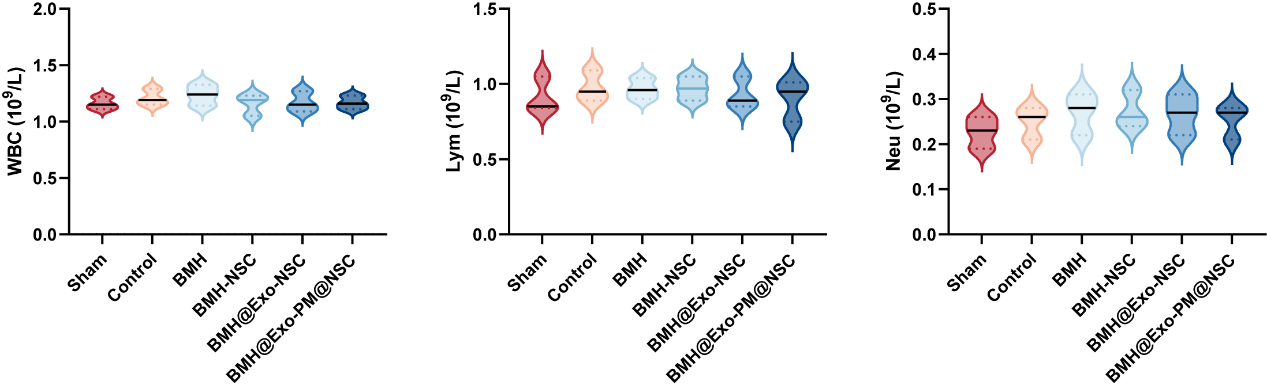
**

**Figure S28.** **Immune response assessment.**

In vivo animal experiments showed that the levels of key blood routine indicators such as WBC (white blood cell), Lym (lymphocyte), and Neu (neutrophil) in SCI rats treated with different therapeutic regimens were within the normal range at 2 months after SCI, indicating that the CTT system (BMH@Exo-PM@NSC) proposed in this study did not induce abnormal immune responses in SCI rats (n=3).
